# Phosphorescent Modulation of Metallophilic Clusters and Recognition of Solvents through a Flexible Host-Guest Assembly: A Theoretical Investigation

**DOI:** 10.3390/nano8090685

**Published:** 2018-09-02

**Authors:** Zhi-Feng Li, Xiao-Ping Yang, Hui-Xue Li, Guo-Fang Zuo

**Affiliations:** 1 College of Chemical Engineering and Technology, Key Laboratory for New Molecule Design and Function of Gansu Universities, Tianshui Normal University, Tianshui 741001, China; li_hx2001@126.com (H.-X.L.); zogofn@126.com (G.-F.Z.); 2 School of Electronic Information and Electrical Engineering, Tianshui Normal University, Tianshui 741001, China; xpyangtsnu@163.com

**Keywords:** TD-DFT, metallophilic, modulation, recognition, spectroscopic properties

## Abstract

MP2 (Second order approximation of Møller–Plesset perturbation theory) and DFT/TD-DFT (Density functional theory/Time-dependent_density_functional_theory) investigations have been performed on metallophilic nanomaterials of host clusters [Au(NHC)_2_]^+^⋅⋅⋅[M(CN)_2_]^−^⋅⋅⋅[Au(NHC)_2_]^+^ (NHC = N-heterocyclic carbene, M = Au, Ag) with high phosphorescence. The phosphorescence quantum yield order of clusters in the experiments was evidenced by their order of *μ*_S1_/Δ*E*_S1−T1_ values ($\mu_{S1}$: S_0_ → S_1_ transition dipole, $∆E_{S1-T1}$: splitting energy between the lowest-lying singlet S_1_ and the triplet excited state T_1_ states). The systematic variation of the guest solvents (**S1**: CH_3_OH, **S2**: CH_3_CH_2_OH, **S3**: H_2_O) are employed not only to illuminate their effect on the metallophilic interaction and phosphorescence but also as the probes to investigate the recognized capacity of the hosts. The simulations revealed that the metallophilic interactions are mainly electrostatic and the guests can subtly modulate the geometries, especially metallophilic Au⋅⋅⋅M distances of the hosts through mutual hydrogen bond interactions. The phosphorescence spectra of hosts are predicted to be blue-shifted under polar solvent and the excitation from HOMO (highest occupied molecular orbital) to LUMO (lowest unoccupied molecular orbital) was found to be responsible for the ^3^MLCT (triplet metal-to-ligand charge transfer) characters in the hosts and host-guest complexes. The results of investigation can be introduced as the clues for the design of promising blue-emitting phosphorescent and functional materials.

## 1. Introduction

The relationship between the luminescent property and metallophilic Au(I)⋅⋅⋅M bond distance (M = Au^I^, Ag^I^, Cu^I^, Tl^I^, Hg^II^, Bi^III^, etc.) has attracted a great deal of attention in the last few years [1,2,3,4,5]. Many experimental and theoretical studies indicated that Au(I) complexes luminesce strongly, especially when the metallophilic interaction is present [6,7,8,9]. The organometallic complexes were applied extensively as emitters in organic light-emitting diodes (OLEDs) and phosphorescent OLEDs with green and red spectral range, which have already been demonstrated to be high efficiency and stability [10,11,12]. However, the blue-emitting OLEDs, which are essential for the commercial launch of devices for lighting, still lack stability and efficiency. Indeed, designing new materials to show higher energy, such as deep-blue emission, encounters more obstacles than the progress made for obtaining green and red colors.

Considerable investigations have been carried out in developing blue OLEDs with high external quantum efficiency as well as a deeper blue color [13,14,15,16,17]. Recently, the highly phosphorescent organometallic nanomaterials of polymeric double salts [Au(NHC)_2_][M(CN)_2_] (NHC = N-heterocyclic carbene, M = Au or Ag) were prepared, which can provide a deep blue shifted phosphorescence spectrum with emission quantum yields of up to 90% [2]. It was found that the extended metallophilic d^10^⋅⋅⋅d^10^ interactions played significant role in the phosphorescence of quasi-2D polymeric nanostructures.

Metallophilic attractions behave similarly to hydrogen bonds [18] which can be easily disrupted/modulated by other crystal packing motifs [18,19,20,21]. Furthermore, the photoluminescent characters might be modulated as metallophilic interaction changed by the functional guest molecules/ions introduced into the M···M interaction systems. Some groups [4,21,22,23,24,25,26,27,28,29,30,31,32] have made significant progress in this area. For example, Catalano et al. introduced BF_4_^−^ and CH_3_OH through anion-cation/anion–π interaction to the hetero-metallic coordinated [AuCu-(PPh_2_py)_3_](BF_4_)_2_ Au(I)···Cu(I) complex [4]. López-de-Luzuriaga and coworkers [30] employed halogen bonding to bimetallic gold–silver clusters. Dichloromethane and tetrahydrofuran also can tune the phosphorescent properties of a Ag/Au metallophilic tetranuclear complex [31]. The coexisting cations [32] can considerably enhance the emission yields of [Au(CN)^2−^] oligomers in aqueous solutions. However, these investigations only observed red-shift phosphorescence and therefore exploration of the blue and highly efficient phosphorescent materials is essential [2].

In the present paper, the metallophilic characters and the phosphorescent properties of the host clusters were investigated by density functional theory (DFT). Introducing the guest solvents CH_3_OH (**S1**), CH_3_CH_2_OH (**S2**), and H_2_O (**S3**), we mainly focus on: (a) the nature of homo/hetero-metallophilic interactions; (b) how the guest molecule affects the metallophilic distance, and (c) how the metallophilic distance affects the photophysical properties of the complexes. Our calculated results reported herein are predicted to provide blue-shift phosphorescence, which is helpful for the further synthesis of the organometallic compounds for blue-emitting OLEDs.

## 2. Computational Methodology

**Figure 1:**
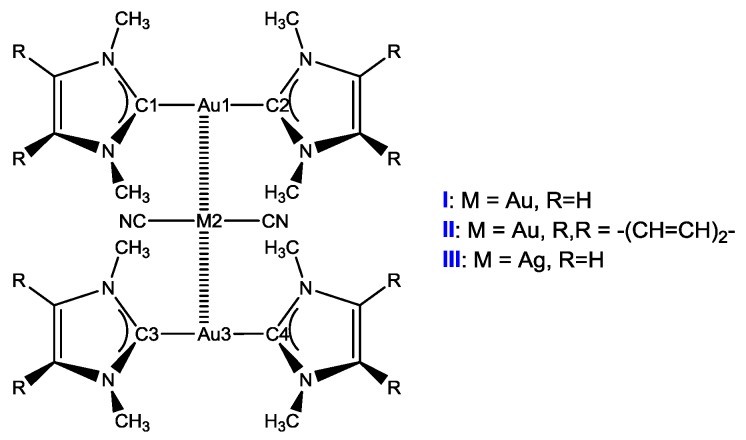
Schematic structure of metallophilic host clusters **X** (**X** = **I**, **II**, and **III**).

Although metallophilic interactions have been investigated with a series of quantum chemical methods, the size of the systems studied renders the use of more computationally expensive methods prohibitive and unfeasible [33,34,35,36,37,38,39,40,41,42,43,44]. The B3LYP [45,46,47], M06-2X [48], *ω*B97XD [49], B3LYP-D3 [50,51], PBE0 [52,53,], and MP2 [54,55] methods were employed respectively to optimize the polymeric ground-state geometries of clusters **X** (**X** = **I**, **II**, and **III**, see Figure 1) on the basis of the experimental X-ray structures. Two basis sets (**BS1**: SDD pseudopotential and basis set for Au and Ag, 6-31G(d) for other atoms; **BS2**: SDD pseudopotential and basis set for Au and Ag, 6-31+G(d,p) for other atoms) were used to obtain reliable geometries of complexes [56,57,58,59,60]. The ground state (S_0_), the lowest excited states (singlet: S_1_; triplet: T_1_) were fully optimized. Triplet states were calculated at the spin unrestricted UPBE0 level with a spin multiplicity of 3.

The MP2 methods [58,59,60,61,62] with **BS1**, **BS3** (aug-cc-pVDZ-PP [63] pseudopotential and basis set for Au and Ag, 6-311++G(d,p) [64,65] for other atoms), and **BS4** (aug-cc-pVTZ-PP pseudopotential and basis set for Au and Ag, 6-311++G(d,p) for other atoms) were also used to investigate the interaction energies with basis set superposition error (BSSE), *E*^CP^ [66]. The different contributions of the interaction energies were obtained by using the GKS-EDA (generalized Kohn−Sham based energy decomposition analysis) [67] methods on the GAMESS [68] platform using B3LYP-D3 with **BS5** (MCP-TZP [69,70,71,72,73,74] for Au and Ag atoms, MCP-DZP [74,75] for other atoms) and **BS6** (MCP-TZP [69,70,71,72,73,74] for all atoms) basis sets.

The geometries of all the structures were fully optimized using the GAUSSIAN09 program suite [76]. The orbital composition analysis is performed by the Multiwfn 3.3 suite of program [77]. The natural bond orbital (NBO) analysis is achieved by NBO 5.0 procedure [78].

## 3. Results and Discussion

We initially optimized the representative host cluster **I** which has seven oligomeric units (Figure S1, see SI) and then calculated **II** and **III** with a similar procedure. The geometrical parameters of the ground state (S_0_) for clusters **I**, **II**, and **III** at various calculated levels are in good agreement with their experimental X-ray structures, respectively. After further extensive testing, PBE0/BS1 was employed in the subsequent qualitative analysis as it was found to be time-economical and reliable to evaluate the geometrical, electronic, and spectral properties of weakly bound metal complexes (Table S1, see SI) [42,43,44,79,80].

### 3.1. Ground States Properties

#### 3.1.1. Structures of Clusters **X**

It should be noted that [Au(NHC)_2_]^+^ is approached vertically to [Au(CN)_2_]^−^ with metallophilic interaction Au···Au in **I**. The Au⋅⋅⋅Au distance of 3.11 Å between [Au(NHC)_2_]^+^- [Au(CN)_2_]^−^ reveals the aurophilic interaction in cluster **I [56]**.

Clusters **II** and **III** have similar geometries to **I**. That is, the anion [Au(CN)_2_]^−^ lies vertically between two cations [Au(NHC)_2_]^+^ and the distances of Au⋅⋅⋅Au and Au⋅⋅⋅Ag are 3.15 and 3.06 Å for **II** and **III**, respectively. Therefore, the metallophilic interaction is decreased in **II** while it is strengthened in **III**, as compared with **I**. Both ∠CMC of anion [M(CN)_2_]^−^ and ∠AuMAu (M = Au, Ag) are 180.0° in cluster **X**, which indicates that three metal atoms together with two CN^–^ ions are in the same plane. The Au⋅⋅⋅M distances in **X** are shorter at least by 0.2 Å than that reported for the tetrameric CF_3_Au⋅CO [5,81], which suggests stronger metallophilic interactions in **X** than that in [CF_3_Au⋅CO]_4_.

**Figure 2:**
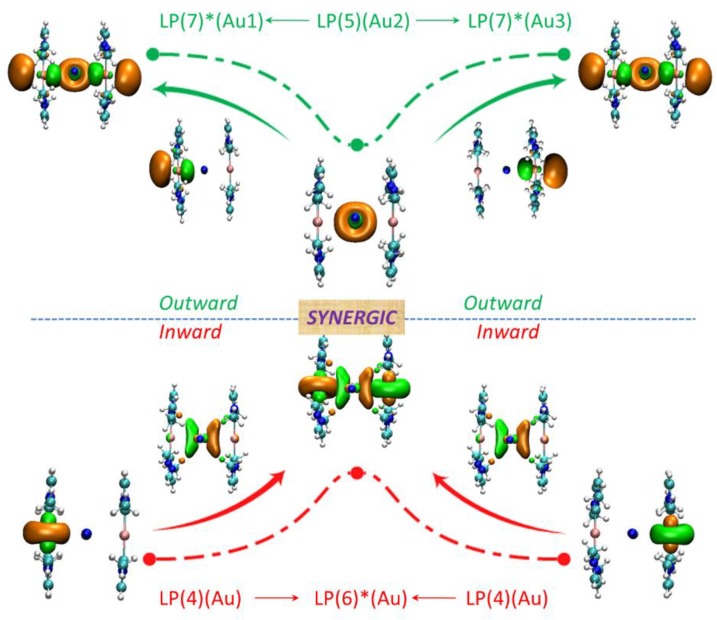
The orbital interaction of aurophilic interaction (outward and inward) in cluster **I**.

The NBO results show that the metallophilic interactions between two adjacent metal atoms are all synergistically bidirectional (outward and inward), which is displayed using cluster **I** as an example (Figure 2, Table S2 in SI). In outward aurophilic interactions, the electron is delocalized from the LP(5) orbital of central Au2 atom to the LP*(7) orbitals of two lateral Au1 and Au3 atoms $E_{ij}^{\left(2\right)}$: ~30 kcal/mol respectively), whereas the electron is donated back from the LP(4) orbitals of Au1 and Au3 atoms to the LP*(6) orbital of central Au1 ($E_{ij}^{\left(2\right)}$: ~28 kcal/mol respectively) in the inward aurophilic interactions. The $E_{ij}^{\left(2\right)}$ of outward (62.83 kcal/mol) is 7 kcal/mol higher than that of inward (55.83 kcal/mol), and also, NBO results show that whether in inward or outward modes, the d orbits of Au atoms act as the electron donors and the electron acceptors originate from the p orbits of Au atoms.

#### 3.1.2. Structures of **X**⋅⋅⋅(**S**)*_x_* Complexes

**Figure 3:**
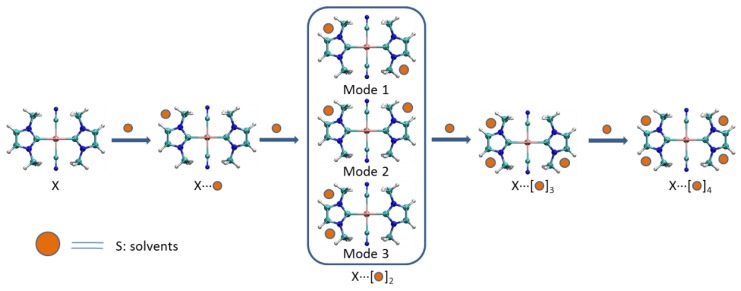
The model of complex formation between host clusters **X** (**X** = **I**, **II**, and **III**) and solvents (CH_3_OH, CH_3_CH_2_OH, and H_2_O).

In this section, the solvents CH_3_OH (**S1**), CH_3_CH_2_OH (**S2**), and H_2_O (**S3**) are introduced to explore how they affect the metallophilic interactions and the phosphorescent properties of **X**. Figure 3 shows the formation model of complexes **X⋅⋅⋅(S)*_x_*** (**X** = **I**–**III**; *x* = 1–4). The skeletal diagram and key structural parameters are also listed in Figure S2 and Table S3, respectively.

Compared to free **I**, the structure of segment **I** in complex **I**⋅⋅⋅**S1** is changed. There are mutual H⋅⋅⋅N and O⋅⋅⋅H hydrogen bonds in complex **I**⋅⋅⋅**S1** and which leads NHC ligands of [Au(NHC)_2_]^+^ to be torsional and the distances of Au1⋅⋅⋅M2 and M2⋅⋅⋅Au3 are not equal anymore.

**Figure 4:**
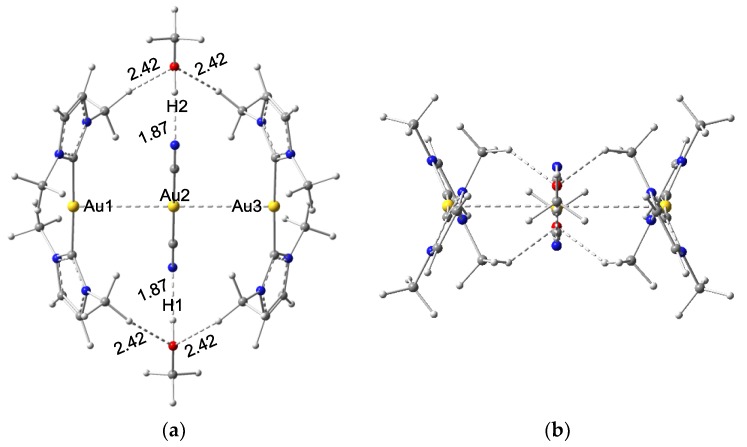
Top view (**a**) and side view (**b**) of the complex **I**⋅⋅⋅(**CH_3_OH**)_2_ (bond length: Å).

Figure 3 shows there are three complexation modes between **X** and two solvent molecules. The energy analysis revealed that mode 1 of **X**⋅⋅⋅(**S)**_2_ is ~ 3 kcal/mol lower than those in modes 2 and 3. Therefore, the following discussions focus on the mode 1. In **I**⋅⋅⋅(**S1)**_2_ (Figure 4), two Au⋅⋅⋅Au distances are elongated to 3.46 Å and the angles ∠H1Au2H2 and ∠Au1Au2Au3 both are 180°. Besides the aurophilic interaction, two types of hydrogen bonds are also involved: two N⋅⋅⋅H–O hydrogen bonds (1.87 Å) and four O⋅⋅⋅H–C bonds (2.42 Å). The **I**⋅⋅⋅(**S1)**_2_ is complexed together by hydrogen bond interactions. 

For **I**⋅⋅⋅(**S1)**_3_, both neighbor Au⋅⋅⋅Au bond distances are 3.40 Å and there are three N⋅⋅⋅H–O (~ 1.9 Å) and six O⋅⋅⋅H–C hydrogen bonds (2.3–2.5 Å). Different from the **I**⋅⋅⋅(**S1)**_2_, the angle ∠Au1Au2Au3 of **I**⋅⋅⋅(**S1)**_3_ is decreased to 158.5°, which reflects that the three gold atoms are not in the same plane anymore.

Complex **I**···(**S1)**_4_ possesses *D*_2h_ symmetry. The skeletons of [Au(CN)_2_]^−^ and **S1** lie in the same plane and they are encapsulated by two [Au(NHC)_2_]^+^ segments. The fourth **S1** for **I**⋅⋅⋅(**S1)**_3_, the Au⋅⋅⋅Au (3.25 Å) is decreased by 0.15 Å and the N⋅⋅⋅H and O⋅⋅⋅H bonds are 1.95 Å and 2.47 Å, respectively.

The structures for **I**⋅⋅⋅(**S2**)*_x_* and **I**⋅⋅⋅(**S3**)*_x_* are similar to those of corresponding **I**⋅⋅⋅(**S1**)*_x_* with the same *x*. For example, the Au⋅⋅⋅Au distances are 3.50, 3.45, 3.40, and 3.25Å for **I**⋅⋅⋅**S2**, **I**⋅⋅⋅(**S2**)_2_, **I**⋅⋅⋅(**S2**)_3_, and **I**⋅⋅⋅(**S2**)_4_, which are relatively close to those for **I**⋅⋅⋅(**S3**)*_x_*. It can be seen from Table S3, the other two metallophilic clusters **II** and **III** can be also adjusted by solvent and their structures are also similar to the those of **I**⋅⋅⋅(**S**)*_x_* respectively. Therefore, the Au⋅⋅⋅M (M = Au, Ag) distances are dramatically elongated with one **S** molecule inserted between two [Au(NHC)_2_]^+^. However, metallophilic Au⋅⋅⋅M distance is decreased gradually as the number of **S** *x* increased. The phosphorescence character then can be predicted to be modulated with the Au⋅⋅⋅M distance changed [1,2,3,4,5].

#### 3.1.3. Interaction Energies

##### Interaction energies of Au⋅⋅⋅M in clusters **X**

The BSSE-corrected interaction energy (obtained from the electronic energy) computations using the GAUSSIAN09 program will be denoted with the superscript CP. The $E_{\mathrm{add}}^{\mathrm{CP}}$ (added) and $E_{\mathrm{tot}}^{\mathrm{CP}}$ (total) interaction energies defined in Equations (1) and (2) [5] are investigated.

(1) $$E_{\mathrm{add}}^{\mathrm{CP}}[X]=E[X]-E[{\left(\mathrm{Au}(\mathrm{NHC}{)}_{2}\right)}^{+}-{{\left(M(\mathrm{CN}\right)}_{2})}^{-}]-E{\left[(\mathrm{Au}(\mathrm{NHC}\right)}_{2}{)}^{+}]+\mathrm{BSSE}$$

(2) $$E_{\mathrm{tot}}^{\mathrm{CP}}[X]=E[X]-2E[{\left(\mathrm{Au}(\mathrm{NHC}{)}_{2}\right)}^{+}-E{{\left(M(\mathrm{CN}\right)}_{2})}^{-}]+\mathrm{BSSE}$$

The calculated level test showed that the PBE0-D3/BS3 method is the most reliable and financial in time to estimate *E*^CP^. Table 1 shows that the interaction energies are very close for the isolated **I**, **II**, and **III**, in which $E_{\mathrm{add}}^{\mathrm{CP}}$ and $E_{\mathrm{tot}}^{\mathrm{CP}}$ are ~−30 and ~−102 kcal/mol, respectively. The clusters **X** $E_{\mathrm{tot}}^{\mathrm{CP}}$ values for two metallophilic bonds are 3–4 times of those of $E_{\mathrm{add}}^{\mathrm{CP}}$ of **X**, suggesting a degree of cooperativity [5,82,83,84].

The EDA interaction energies of clusters **I**–**III** are reported in Table 2, where the contributors *E*_es_, *E*_ex_, *E*_pol_, *E*_disp_, and *E*_corr_ are attractive and *E*_rep_ is repulsive. It should be noted that *E*_add_ and *E*_tot_ values are very close to their corresponding electrostatic energy (*E*_es_) term, indicating that the clusters are mainly stabilized by the electrostatic interactions [5,84].

##### Interaction energies of complexes **X**⋅⋅⋅(**S**)_*x*_

In this section, the total interaction energies *E*^CP^ and the *E*^CP*n*^ (*n* = 1, 2, 3, and 4) are considered. The *E*^CP1^, *E*^CP2^, *E*^CP3^, and *E*^CP4^ respectively correspond to the interaction energies between one, two, three, and four **S** molecules and the remainder parts in **X**⋅⋅⋅(**S)***_x_* (Table 3). For **I**⋅⋅⋅(**S1**)*_x_*, the *E*^CP1^ value is decreased from −16.6 to −11.8 kcal/mol as the number of **S1** increasing from one to four. The *E*^CP*n*^ value for **I**⋅⋅⋅(**S2**)*_x_* is very similar with the case in **I**⋅⋅⋅(**S1**)*_x_*, indicating that the solvents **S1** and **S2** behave similar in controlling the interaction energies for **I**⋅⋅⋅(**S)***_x_*. Furthermore, the solvents **S1** and **S2** also act very similarly to adjust the interaction energy for **II**⋅⋅⋅(**S)***_x_* or **III**⋅⋅⋅(**S)***_x_*. Therefore, the interaction energy might not be adjusted by the carbon chain growth. All *E*^CP*n*^ values for **X**⋅⋅⋅(**S)***_x_* are negative, which shows **S** can stabilize the **X**⋅⋅⋅(**S)***_x_* complexes.

The GKS-EDA results (Figure S3a–c) of complexes **X**⋅⋅⋅(**S2)***_x_* [85] further reveal that the solvents **S1** and **S2** contribute very similar behavior to change the interaction energy for **II**⋅⋅⋅(**S)***_x_* or **III**⋅⋅⋅(**S)***_x_*. In other words, the EDA values have small differences between **X**⋅⋅⋅(**S1**)*_x_* and **X**⋅⋅⋅(**S2**)*_x_* at the same *x* with the same **X**. The EDA results showed that *E*_ex_ is the most important energy component for all complexes except **X**⋅⋅⋅**S2**, in which electrostatic force is most important (Table S4).

Interestingly, the energy contributors of *E*_ex_, *E*_rep_, *E*_pol_, *E*_disp_, and *E*_corr_ are summed close to zero and an excellent correlation R = 1.00 between the *E*_tot_ and the *E*_es_ values was found for **I**⋅⋅⋅(**S2**)*_x_*, **II**⋅⋅⋅(**S2**)*_x_*, and **III**⋅⋅⋅(**S2**)*_x_* with the linear equation (Figure S3a′–c′). These results further reinforce our finding that the investigated interaction is governed by the electrostatic term (Figure S4). 

### 3.2. Excited State’s Properties

The M-related bond lengths, bond angles, and the major geometrical changes between the ground state S_0_ and lowest lying triplet excited state T_1_ are summarized in Table 4. The parameters reveal that the Au1⋅⋅⋅M2 and Au3⋅⋅⋅M2 bonds are shorter maximally by ~0.40 Å for T_1_ than those for the corresponding S_0_ of the cluster **X**. The metallophilic Au⋅⋅⋅M distance in S_0_ can be increased by introducing **S** into cluster **X**. However, the Au⋅⋅⋅M distances are shortened by 0.35 Å for triplet **III**⋅⋅⋅(**S1**)_4_ and even by 0.80 Å for triplet **III**⋅⋅⋅**S1** as compared with the corresponding S_0_ of **III**⋅⋅⋅(**S)***_x_*, respectively.

Table 5 shows the calculated emission energies, the electron transition assignments, and the experimental values of complexes **I**–**III**. The calculated emission wavelengths of 457, 483, and 417 nm are in good agreement with the experimental emission values of 448, 465, and 446 nm, which respectively correspond to the clusters **I**, **II,** and **III**. The electron transition from HOMO to LUMO is responsible for the emission at 457 nm for **I**. The HOMO of cluster **I** mainly consist of natural atomic orbital (NAO) of Au (77.23%, d: 45.29%), while LUMO has less NAO of Au (35.71%) and more significant NAO of the ligands (64.29%), which is MLCT character from the metal-centred to ligands excited states (Table S5). **II** and **III** also display similar MLCT phosphorescence nature to **I** (Table S6).

The phosphorescent emission wavelengths can be tuned as the Au–M distances change with the solvent effect (Table S3). The Au⋅⋅⋅M distances in S_0_ of **X**⋅⋅⋅(**S)***_x_* are lengthened compared to those in free **X**. However, the Au⋅⋅⋅M distances in **X**⋅⋅⋅(**S)***_x_* are dramatically shorter in the T_1_ state than those in S_0_. Actually, Au⋅⋅⋅M distances in **X**⋅⋅⋅(**S)***_x_* are changed little compared with those in free **X** no matter with the type and the number of **S** in the triplet state, which is significantly different from the case in S_0_. Therefore, the Au⋅⋅⋅M distance change in S_0_ as *x* increases can reflect the photoluminescence change since the phosphorescent emission involves electron transition from T_1_ to S_0_.

It can be seen obviously that the photoluminescence of **X** can be tuned (Δλ) by Au⋅⋅⋅M distance change (Δ*d*) through introducing **S** to **X**, and photoluminescence of the **X**⋅⋅⋅(**S)***_x_* complexes shows blue shift compared with free **X** (Table S3 and Figure S5). Especially, **X**⋅⋅⋅(**S)**_4_ (X = I, III) provide the largest blue-shifted photoluminescence, while **II**⋅⋅⋅(**S)**_4_ has the smallest blue-shifted emission wavelengths. The photoluminescent emission wavelength for **II**⋅⋅⋅**S** is significantly different from those for **II**⋅⋅⋅(**S)***_x_* (*x* = 2, 3, 4) because **I** is distorted in **I**⋅⋅⋅**S**. However, similar to the photoluminescence of **X**, the excitations from HOMO to LUMO of **X**⋅⋅⋅(**S**)*_x_* are still responsible for their emission at their maximum wavelength which mainly originates from MLCT character between metal-centred and ligands (Tables S5 and S6). The MLCT character becomes most obvious at *x* = 4 because of the charge transfer capacity from metal-centred to ligands of ~38%, ~44%, and ~31% for **I**⋅⋅⋅(**S)**_4_, **II**⋅⋅⋅(**S)**_4_, and **III**⋅⋅⋅(**S)**_4_, respectively. Moreover, different solvents, **S1** and **S2,** have little effect on MLCT character of **X** at specific *x*. For example, the charge transfer capacity for **I**⋅⋅⋅(**S1**)_4_ and **I**⋅⋅⋅(**S2**)_4_ are respectively 37.63% and 37.60% (Table S5) so that the maximum photoluminescent wavelengths of them are also the same with 412 nm.

The radiative (*k*_r_) and the non-radiative (*k*_nr_) rate constants are linked to the phosphorescence quantum yield (*Φ*_PL_) from an emissive excited state to the ground state by Equation (3).

*Φ*_PL_ = *k*_r_/(*k*_r_ + *k*_nr_) (3)

Theoretically, *k*_r_ is related to the mixing between S_1_ and T_1_, which is proportional to the spin-orbit coupling (SOC) rate constants. SOC rate constants are linked to the phosphorescence quantum efficiency between the two states, according to Equation (4).

(4) $$k_{r}=\gamma \frac{\langle \Psi_{S_{1}}\left|H_{S_{0}}\right|\Psi_{S_{0}}\rangle {}^{2}\cdot \mu_{S_{1}}{}^{2}}{{\left(\Delta E_{S_{1}-T_{1}}\right)}^{2}},\;\mathrm{with}\;\gamma =\frac{16\pi^{2}10^{6}n^{3}E_{\mathrm{em}}{}^{3}}{3h\varepsilon_{0}}$$

In general, smaller $\Delta E_{S_{1}-T_{1}}$ and larger $\mu_{S_{1}}$ or $\frac{\mu_{S_{1}}}{\Delta E_{S_{1}-T_{1}}}$ for the system could induce a higher *Φ*_PL_ [86]. The *μ*_S1_/Δ*E*_S1−T1_ values for complexes **X** are in the order: 0.99 (**I**) 0.91 (**II**) 0.83 (**III**), which is rationalized by the *Φ*_PL_ in experimental order: 90% (**I**) 67% (**II**) 11% (**III**). An excellent correlation (*R* = 0.943) was observed between the theoretical $\frac{\mu_{S_{1}}}{\Delta E_{S_{1}-T_{1}}}$ values and the experimental *Φ*_PL_ values with the linear equation *Φ*_PL_ = −393.3 − 493.8 × $\frac{\mu_{S_{1}}}{\Delta E_{S_{1}-T_{1}}}$ for free **X** (Figure S6). We further employ this equation to predict the *Φ*_PL_ values of **X**⋅⋅⋅(**S)***_x_*, which reveal that fourteen complexes have high phosphorescence efficiency with *Φ*_PL_ larger than 50% (Table S7, see SI).

## 4. Conclusions

In summary, a detailed study of metallophilic Au⋅⋅⋅M bonding in host clusters from self-assembled [Au(NHC)_2_]^+^ and [M(CN)_2_]^−^ (M = Au, Ag) and the characters of their host–guest complexes are presented by the second-order Møller−Plesset (MP2) method, density functional theory, and qualitative analysis via GKS-EDA and NBO methods with a series of basis sets. 

The largest and smallest *μ*_S1_/Δ*E*_S1−T1_ values for cluster **I** and **III,** respectively, were the highest and lowest *Φ*_PL_ among three experimental clusters **I**–**III**. Based on host–guest complexation, the phosphorescence of hosts can be modulated and the guest solvents can be recognized. The metallophilic interactions are mainly derived from electrostatic interaction. The solvents methanol, ethanol, and water can adjust the geometries of Au(I)⋅⋅⋅Au(I)/Ag(I) clusters with H-bond interaction between the cluster and the solvents, especially changing the distance between two neighbor metal centres, leading to blue-shift phosphorescence of the clusters. These results highlight that the metal⋅⋅⋅metal interaction and the photoluminescence characters of the clusters can be functionally controlled by solvent molecules. The linear relationship between Δλ and Δ*d* and the binding energies of host–guest complexes suggest that the phosphorescence wavelength shift can be predicted through the M⋅⋅⋅M distances in T_1_ states, and the interactions in clusters can offer potential applications in solvent and catalysis transport and recognition using synthetic functional materials in the future. This work will be expanded upon by employing related binuclear complexes under systematic variation of the metal ions and solvents.

## Figures and Tables

**Table 1:** Interaction energies (*E*^CP^_add_ and *E*^CP^_tot_) for clusters **I**–**III** at PBE0-D3/BS3// PBE0/BS1 level (kcal/mol).

I			II			III	
*E* ^CP^_add_	*E* ^CP^_tot_		*E* ^CP^_add_	*E* ^CP^_tot_		*E* ^CP^_add_	*E* ^CP^_tot_
−29.0	−102.5		−29.7	−101.2		−29.7	−103.6

**Table 2:** The GKS-EDA results of **I**–**III** at B3LYP-D3/BS5 level (kcal/mol).

	Mode	*E*_es_	*E*_ex_	*E*_rep_	*E*_pol_	*E*_disp_	*E*_corr_	*E*_tot_
**I**	*E*_add_	−23.3	−46.0	74.8	−15.6	−14.0	−5.6	−29.6
	*E*_tot_	−95.8	−99.5	159.8	−29.9	−26.2	−12.5	−104.0
**II**	*E*_add_	−22.5	−43.0	69.7	−15.1	−14.8	−5.1	−30.7
	*E*_tot_	−92.2	−94.0	150.7	−29.4	−27.0	−11.2	−103.0
III *	*E*_add_	−23.7	−36.7	60.3	−11.7	−16.9		−28.7
	*E*_tot_	−96.1	−78.9	127.8	−22.0	−31.6		−100.9

* Calculated with LMO-EDA (localized molecular orbital energy decomposition analysis) at MP2/BS6 level.

**Table 3:** Interaction energies (kcal/mol) of **X**⋅⋅⋅(**S**)*_x_*.

(S)*_x_*	I						II						III				
	*E* ^CP1^	*E* ^CP2^	*E* ^CP3^	*E* ^CP4^	*E* ^CP^		*E* ^CP1^	*E* ^CP2^	*E* ^CP3^	*E* ^CP4^	*E* ^CP^		*E* ^CP1^	*E* ^CP2^	*E* ^CP3^	*E* ^CP4^	*E* ^CP^
S1	−16.6				−111.6		−15.8				−109.8		−17.1				−111.5
(S1)_2_	−15.3	−30.7			−124.8		−15.4	−30.9			−123.9		−15.8	−31.5			−124.7
(S1)_3_	−12.5	−25.7	−42.0		−136.2		−13.7	−30.0	−44.0		−137.7		−12.9	−26.4	−43.1		−136.6
(S1)_4_	−11.8	−23.6	−37.1	−50.7	−146.3		−13.0	−26.0	−40.6	−55.2	−150.5		−12.2	−24.3	−38.2	−52.0	−147.5
S2	−16.7				−112.0		−16.4				−110.2		−17.3				−111.8
(S2)_2_	−15.6	−31.2			−125.4		−16.0	−32.1			−125.0		−16.0	−32.0			−125.2
(S2)_3_	−12.8	−26.2	−42.8		−179.8		−14.1	−28.3	−45.2		−139.1		−13.3	−30.2	−43.8		−137.3
(S2)_4_	−12.2	−25.4	−38.0	−51.6	−147.5		−13.4	−26.7	−41.6	−56.5	−152.0		−12.5	−25.0	−39.0	−53.0	−148.4
S3	−15.2				−110.5		−14.5				−108.4		−15.8				−110.4
(S3)_2_	−14.1	−28.3			−122.5		−14.0	−28.0			−121.4		−14.6	−29.2			−122.3
(S3)_3_	−10.6	−23.5	−38.8		−132.6		−10.9	−27.7	−41.4		−133.4		−10.9	−24.1	−39.8		−133.0
(S3)_4_	−10.5	−20.9	−33.3	−45.7	−141.8		−11.9	−23.9	−37.7	−51.5	−147.5		−10.8	−21.5	−34.3	−47.0	−142.3

**Table 4:** Selected optimized parameters for **I**–**III** (bond length: Å, bond angle: °) *^a^*.

State	Cplx *^b^*	Au-C1	Au-C2	M-C3	M-C4	Au-C5	Au-C6	Au1-M2	M2-Au3	Au1M2Au3
	**I**	2.04	2.04	2.00	2.00	2.04	2.04	3.12	3.11	179.98
**S_0_**	**II**	2.04	2.04	2.00	2.00	2.04	2.04	3.15	3.15	180.00
	**III**	2.04	2.04	2.05	2.05	2.04	2.04	3.06	3.06	180.00
	**I**	2.03	2.03	1.99	1.99	2.03	2.03	2.77	2.77	179.99
**T_1_**	**II**	2.03	2.03	1.99	1.99	2.03	2.03	2.76	2.75	179.98
	**III**	2.03	2.03	2.02	2.02	2.03	2.03	2.76	2.76	179.99
	**I**	−0.01	−0.01	−0.01	−0.01	−0.01	−0.01	−0.35	−0.34	0.01
**Δ**	**II**	−0.01	−0.01	−0.01	−0.01	−0.01	−0.01	−0.39	−0.40	−0.02
	**III**	−0.01	−0.01	−0.03	−0.03	−0.01	−0.01	−0.30	−0.01	−0.30

*^a^* the atom numbering scheme is given in Figure 1. *^b^* Cplx = Complex. Δ = the different between bond length/bond angle in T_1_ and S_0_ state.

**Table 5:** Calculated phosphorescent emission (in nm) of the studied clusters **I**–**III** with TD-DFT method, along with experimental values.

Complex	λ/*E*(eV)	Configuration	Character	Exp *^a^*	δ
**I**	457/2.72	HOMO-LUMO (98%)	LMCT	448	9
**II**	483/2.57	HOMO-LUMO (97%)	LMCT	465	18
**III**	417/2.98	HOMO-LUMO (97%)	LMCT	446	-29

*^a^* reference [11], δ = cal-exp.

## Supplementary Materials

The following are available online at http://www.mdpi.com/2079-4991/8/9/685/s1, Figure S1: Structure diagrams of clusters, Figure S2: Top view (a) and face view (b) of the representative cluster **X**⋅⋅⋅(S)_*x*_ (**X** = **I**–**III**; *x* = 1–4), Figure S3: Trends of interaction energies (a, b, c) and the relationships between the *E*_es_ and *E*_tot_ (a′, b′, c′), Figure S4, The relationships between the *E*_es_ and *E*_tot_ (a) **I**⋅⋅⋅(S2)*_x_*, (b) **II**⋅⋅⋅(S1)*_x_*, (c) **II**⋅⋅⋅(S2)*_x_* and (d) **III**⋅⋅⋅(S2)*_x_* for the *E*^CP1^ of clusters, Figure S5: The relationship between Δd and Δλ for complexes **X**⋅⋅⋅(**S**)*_x_* in T1 states, Figure S6: The relationship between μ_S1_/Δ*E*_S1−T1_ values in theory and the *Φ*_PL_ values in experiment, Table S1: Comparing the calculated and experimental parameters for the different oligomeric I (bond length: Ǻ, wavelength: nm), Table S2: The NBO results of the clusters, Table S3: The key parameters and the phosphorescent character of complexes **X**⋅⋅⋅(**S**)*_x_*, Table S4: Selected GKS-EDA results of **X**⋅⋅⋅(**S***n*)*_x_*, Table S5: Molecular Orbitals and their main consist of natural atomic orbital and the characters, Table S6: Calculated phosphorescent emission (in nm) of the studied complexes with the TDDFT method, along with experimental values, Table S7: The singlet-triplet splitting energy (Δ*E*_S1−T1_, in eV), transition electric dipole moment (*μ*_S1_, in atomics units), the *μ*_S1_/Δ*E*_S1−T1_, and the predicted *Φ*_PL_ ( 50%) for complexes **X**⋅⋅⋅(**S**)*_x_*, Table S8: The *E*^CP^ of cluster I at different calculated levels (kcal/mol), Table S9: The EDA results of cluster I at different calculated levels (kcal/mol).
