# Supplementary material for: Phosphorescent Modulation of Metallophilic Clusters and Recognition of Solvents through a Flexible Host-Guest Assembly: A Theoretical Investigation
Source: Nanomaterials (Basel). 2018 Sep 2;8(9):685. doi: 10.3390/nano8090685 (PMC6163230; doi:10.3390/nano8090685)
Supplement: Supplementary file 1 [file nanomaterials-08-00685-s001.pdf]

Phosphorescent Modulation of Metallophilic Cluster and Recognition of Solvents through Flexible Host-Guest Assembly: A Theoretical Investigation

Zhi-Feng Li [1,*], Xiao-Ping Yang [2], Hui-Xue Li [1] and Guo-Fang Zuo [1]

[1] College of Chemical Engineering and Technology, Key Laboratory for New Molecule Design and Function of Gansu Universities, Tianshui Normal University, Tianshui 741001, China

[2] School of Electronic Information and Electrical Engineering, Tianshui Normal University, Tianshui 741001, China

1. Figure 1S 2
2. Table 1S 3
3. Table 2S 4
4. Figure 2S 6
5. Table 3S 7
6. Table 4S 9
7. Figure 3S 11
8. Figure 4S 15
9. Table 5S 13
10. Table 6S 14
11. Figure 5S 16
12. Figure 6S 16
13. Table 7S 17
14. Table 8S 18
15. Table 9S 19
16. The S_0 .xyz files of I and its complexes 20
17. The S_0 .xyz files of II and its complexes 27
18. The S_0 .xyz files of III and its complexes 35
19. The T_1 .xyz files of I and its complexes 42
20. The T_1 .xyz files of II and its complexes 46
21. The T_1 .xyz files of III and its complexes 54

1. Figure 1S

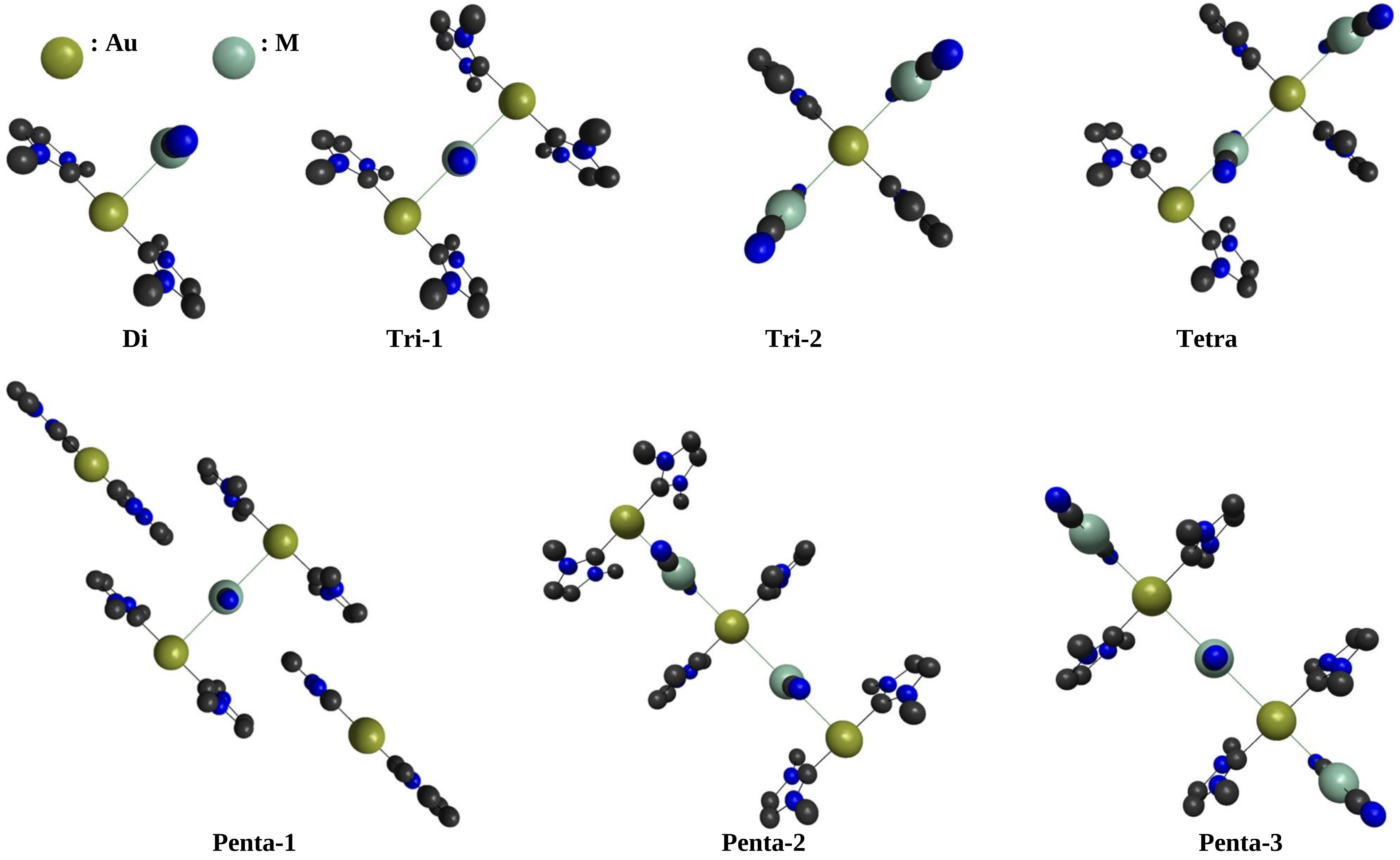

Figure 1S. Structure diagrams of clusters.

2. Table 1S

Table 1S. Comparing the calculated and experimental parameters for the different oligomeric I (bond length: Å, wavelength: nm).

Cluster	Methods	BS1			BS2	
		d^{cal} (Au···Au)	d^{cal}-d^{exp}*	$\lambda_{max}^{cal} - \lambda_{max}^{exp}$ **	d^{cal} (Au···Au)	d^{cal}-d^{exp}*
Di	B3LYP	3.21	0.06	−60.07	3.24	0.09
	B3LYP-D3	3.11	−0.04	57.91	3.12	−0.03
	PBE0	3.08	-0.07	−55.38	3.09	−0.06
	ωB97XD	3.21	0.06	−97.45	3.22	0.07
	M062X	3.20	0.05	−99.38	3.23	0.08
	MP2	2.99	−0.16		-	-
Tri-1	B3LYP	3.37	0.22	−11.39	-	-
	B3LYP-D3	3.06	−0.09		3.06	−0.09
	PBE0	3.12	−0.03	8.56	3.13	−0.02
	ωB97XD	3.18	0.03	−51.9	3.11	−0.04
	M062X	3.32	0.17		3.37	0.22
	MP2	2.94	−0.21		-	-
Tri-2	B3LYP	5.74	2.59		5.84	2.69
	B3LYP-D3	4.25	1.10		4.20	1.05
	PBE0	3.21	0.06		3.23	0.08
	ωB97XD	4.26	1.11		4.23	1.08
	M062X	4.29	1.14		4.20	1.05
Tetra	B3LYP	3.26	0.11	88.17		
	PBE0	3.11	−0.04	111.25		
Penta-1	ωB97XD	3.16	0.01	−57.19		
	M062X	3.23	0.08	−76.66		
	B3LYP	>4.0	-			
	B3LYP-D3	-	-			
	PBE0	-	-			
Penta-2	B3LYP	3.27	0.12			
	B3LYP-D3	-	-			
	PBE0	3.16	0.01	95.64		
	ωB97XD	-	-			
	M062X	3.54	0.39			
Penta-3	B3LYP	-	-			
	B3LYP-D3	>3.8	-			
	PBE0	>3.3	-			
	ωB97XD					
	M062X	>3.7				

*d^{exp} = 3.15 Å, **λ_{max}^{exp}= 448 nm.

It can be seen from Table 1S, the Au-M distances of **Penta-1** at *ω*B97XD/BS1 and **Penta-2** at PBE0/BS1 are only beyond the experimental data 0.01 Å respectively as well as the **Tri-1** at PBE0/BS1 is shorter by 0.03 Å than the experiment. These cases indicate that the PBE0/BS1 is reliable for the complexes and the **Penta-1**, **Penta-2**, and **Tri-1** can act as plausible cluster unit with the geometrical parameters as the reference. In order to further verify which cluster is the most represented mode for studying, the calculated phosphorescent character of them are listed in Table S1 (ESI†). It can be seen that the calculated phosphorescent wavelength of **Tri-1** is the most close to the experiment and the difference only is only about 9 nm.

3. Table 2S

complex					complex				
	donor	acceptor	Eij			donor	acceptor	Eij	
I	LP(5)(Au2)	LP(7)*(Au1)	32.41	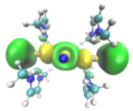	II-2S2	LP(3)(Au2)	LP(7)*(Au1)	15.64	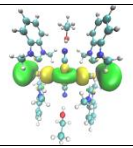
		LP(7)*(Au3)	30.42				LP(7)*(Au3)	15.64	
	LP(4)(Au1)	LP(6)*(Au2)	29.47	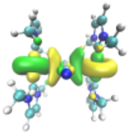		LP(3)(Au1)	LP(6)*(Au2)	14.18	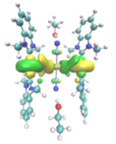
	LP(4)(Au3)		26.36			LP(3)(Au3)		14.18	
II	LP(5)(Au2)	LP(6)*(Au1)	31.78	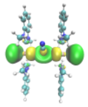	II-3S2	LP(4)(Au2)	LP(7)*(Au1)	16.43	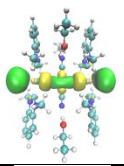
		LP(7)*(Au3)	31.84				LP(7)*(Au3)	16.43	
	LP(4)(Au1)	LP(6)*(Au2)	24.39	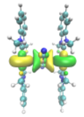		LP(4)(Au1)	LP(7)*(Au2)	16.08	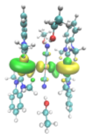
	LP(4)(Au3)		24.44			LP(4)(Au3)		16.08	
III	LP(5)(Ag2)	LP(7)*(Au1)	16.87	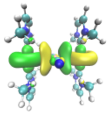	II-4S2	LP(5)(Au2)	LP(7)*(Au1)	25.07	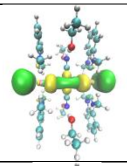
		LP(7)*(Au3)	16.87				LP(7)*(Au3)	25.07	
	LP(4)(Au1)	LP(6)*(Ag2)	26.76	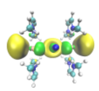		LP(4)(Au1)	LP(7)*(Au2)	21.21	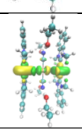
	LP(4)(Au3)		26.76			LP(4)(Au3)		21.21	
I-S2	LP(3)(Au2)	LP(7)*(Au1)	12.87	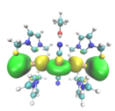	III-S2	LP(5)(Ag2)	LP(7)*(Au1)	4.49	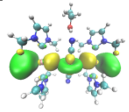
		LP(7)*(Au3)	12.87				LP(7)*(Au3)	4.78	
	LP(3)(Au1)	LP(6)*(Au2)	13.86	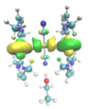		LP(3)(Au1)	LP(6)*(Ag2)	13.98	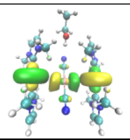
	LP(3)(Au3)		13.85			LP(3)(Au3)		13.14	

I-2S2	LP(3)(Au2)	LP(7)*(Au1)	14.8	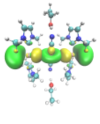	III-2S2	LP(5)(Ag2)	LP(7)*(Au1)	5.39	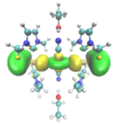
		LP(7)*(Au3)	14.81				LP(7)*(Au3)	5.39	
	LP(3)(Au1)	LP(6)*(Au2)	15.78	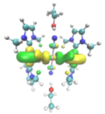		LP(3)(Au1)	LP(6)*(Ag2)	11.06	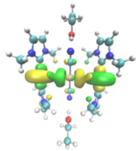
	LP(3)(Au3)		15.75			LP(3)(Au3)		11.06	
I-3S2	LP(3)(Au2)	LP(7)*(Au1)	13.21	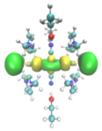	III-3S2	LP(5)(Ag2)	LP(7)*(Au1)	4.52	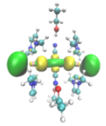
		LP(7)*(Au3)	13.72				LP(7)*(Au3)	4.64	
	LP(4)(Au1)	LP(7)*(Au2)	13.04	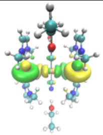		LP(4)(Au1)	LP(6)*(Ag2)	13.09	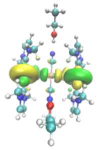
	LP(4)(Au3)		14.32			LP(4)(Au3)		14.9	
I-4S2	LP(4)(Au2)	LP(7)*(Au1)	24.43	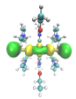	II-4S2	LP(5)(Ag2)	LP(7)*(Au1)	10.46	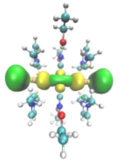
		LP(7)*(Au3)	24.41				LP(7)*(Au3)	10.46	
	LP(4)(Au1)	LP(7)*(Au2)	22.14	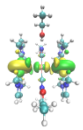		LP(4)(Au1)	LP(6)*(Ag2)	19.09	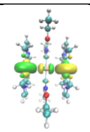
	LP(4)(Au3)		22.14			LP(4)(Au3)		19.09	
II-S2	LP(3)(Au2)	LP(7)*(Au1)	13.01	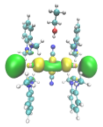					
		LP(7)*(Au3)	13.37						
	LP(3)(Au1)	LP(6)*(Au2)	12.42	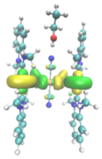					
	LP(3)(Au3)		12.28						

4. Figure 2S

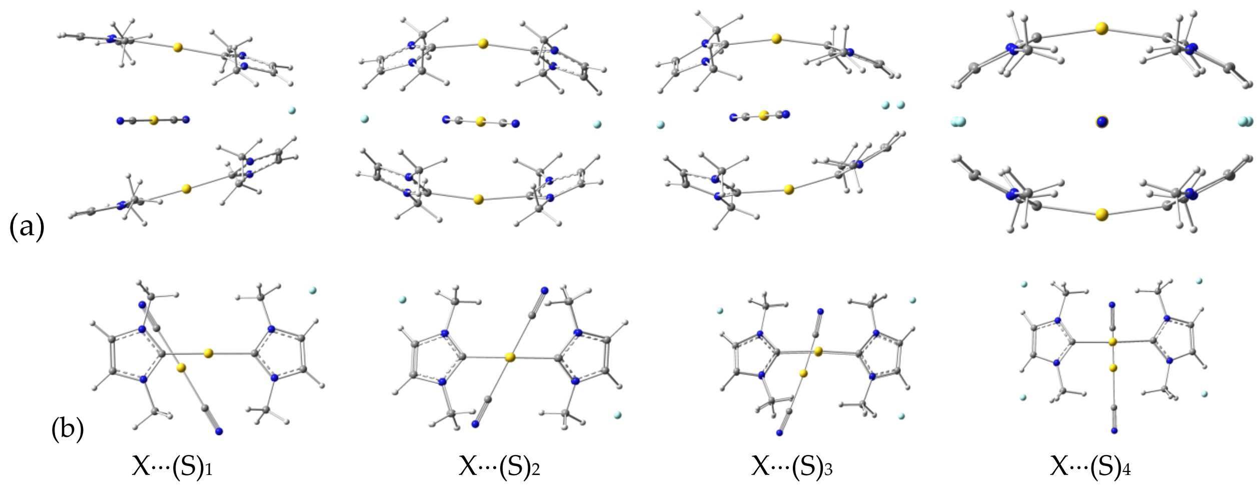

Figure 2S. Top view (**a**) and face view (**b**) of the representative cluster **X**···(**S**)$_x$ (X = I–III; x = 1–4)

5. Table 3S

Table 3S. The key parameters and the phosphorescent character of complexes **X**···(**S**)$_x$ (bond length: Å, bond angle:°, wavelength: nm).

	d0(S_0)														
	I	I-S1	I-2S1	I-3S1	I-4S1	II	II-S1	II-2S1	II-3S1	II-4S1	III	III-S1	III-2S1	III-3S1	III-4S1
Au1-M2	3.12	3.54	3.46	3.40	3.25	3.15	3.53	3.44	3.36	3.22	3.06	3.56	3.44	3.42	3.22
M2-Au3	3.11	3.54	3.46	3.40	3.25	3.15	3.51	3.44	3.36	3.22	3.06	3.56	3.44	3.39	3.22
Au-C1	2.04	2.04	2.04	2.04	2.04	2.04	2.04	2.04	2.04	2.04	2.04	2.04	2.04	2.04	2.04
Au-C2	2.04	2.04	2.04	2.04	2.04	2.04	2.04	2.04	2.04	2.04	2.04	2.04	2.04	2.04	2.04
M-C3	2.00	1.99	1.99	1.99	1.99	2.00	1.99	1.99	1.99	1.99	2.05	2.04	2.04	2.04	2.05
M-C4	2.00	1.99	1.99	1.99	1.99	2.00	1.99	1.99	1.99	1.99	2.05	2.04	2.04	2.04	2.05
Au-C5	2.04	2.04	2.04	2.04	2.04	2.04	2.04	2.04	2.04	2.04	2.04	2.04	2.04	2.04	2.04
Au-C6	2.04	2.04	2.04	2.04	2.04	2.04	2.04	2.04	2.04	2.04	2.04	2.04	2.04	2.04	2.04
Au1M2Au3	180.0	168.3	180.0	159.1	180.0	180.0	151.7	180.0	165.5	180.0	180.0	165.9	180.0	157.1	180.0
	d1(T_1)														
Au1-M2	2.77	2.77	2.84	2.85	2.86	2.76	2.76	2.86	2.86	2.85	2.76	2.76	2.85	2.86	2.87
M2-Au3	2.77	2.77	2.84	2.85	2.86	2.75	2.76	2.86	2.86	2.85	2.76	2.76	2.85	2.86	2.87
Au-C1	2.03	2.03	2.03	2.03	2.04	2.03	2.03	2.03	2.03	2.03	2.03	2.03	2.03	2.04	2.04
Au-C2	2.03	2.03	2.03	2.03	2.04	2.03	2.03	2.03	2.03	2.03	2.03	2.03	2.03	2.04	2.04
M-C3	1.99	1.99	1.99	1.99	1.98	1.99	1.99	1.99	1.99	1.99	2.02	2.02	2.02	2.02	2.01
M-C4	1.99	1.99	1.99	1.99	1.98	1.99	1.99	1.99	1.99	1.99	2.02	2.02	2.02	2.02	2.01
Au-C5	2.03	2.03	2.03	2.03	2.04	2.03	2.03	2.03	2.03	2.03	2.03	2.03	2.03	2.04	2.04
Au-C6	2.03	2.03	2.03	2.03	2.04	2.03	2.03	2.03	2.03	2.03	2.03	2.03	2.03	2.04	2.04
Au1M2Au3	180.0	176.8	180.0	173.1	180.0	180.0	178.2	180.0	175.8	180.0	180.0	176.3	180.0	172.4	179.9
λ	457	444	419	418	412	483	478	437	439	443	417	409	383	379	371
Δλ		-13	-38	-39	-45		-5	-46	-44	-40		-8	-34	-38	-46
	d0(S_0)														
	I	I-S2	I-2S2	I-3S2	I-4S2	II	II-S2	II-2S2	II-3S2	II-4S2	III	III-S2	III-2S2	III-3S2	III-4S2
Au1-M2	3.12	3.50	3.45	3.41	3.25	3.15	3.51	3.45	3.35	3.22	3.06	3.54	3.44	3.42	3.24
M2-Au3	3.11	3.50	3.45	3.41	3.25	3.15	3.54	3.45	3.35	3.22	3.06	3.55	3.44	3.39	3.24
Au-C1	2.04	2.04	2.04	2.04	2.04	2.04	2.04	2.04	2.04	2.04	2.04	2.04	2.04	2.04	2.04
Au-C2	2.04	2.04	2.04	2.04	2.04	2.04	2.04	2.04	2.04	2.04	2.04	2.04	2.04	2.04	2.04
M-C3	2.00	1.99	1.99	1.99	1.99	2.00	1.99	1.99	1.99	1.99	2.05	2.05	2.04	2.05	2.05
M-C4	2.00	1.99	1.99	1.99	1.99	2.00	1.99	1.99	1.99	1.99	2.05	2.05	2.04	2.05	2.05
Au-C5	2.04	2.04	2.04	2.04	2.04	2.04	2.04	2.04	2.04	2.04	2.04	2.04	2.04	2.04	2.04
Au-C6	2.04	2.04	2.04	2.04	2.04	2.04	2.04	2.04	2.04	2.04	2.04	2.04	2.04	2.04	2.04
Au1M2Au3	180.0	170.3	180.0	159.1	180.0	180.0	152.2	179.9	166.0	180.0	180.0	166.8	180.0	157.2	179.9

							d1(T_1)								
Au1-M2	2.77	2.77	2.84	2.85	2.85	2.76	2.76	2.86	2.85	2.85	2.76	2.76	2.85	2.86	2.87
M2-Au3	2.77	2.77	2.84	2.85	2.85	2.75	2.76	2.86	2.85	2.85	2.76	2.76	2.85	2.86	2.87
Au-C1	2.03	2.03	2.03	2.04	2.04	2.03	2.03	2.03	2.03	2.03	2.03	2.03	2.03	2.04	2.04
Au-C2	2.03	2.03	2.03	2.04	2.04	2.03	2.03	2.03	2.03	2.03	2.03	2.03	2.03	2.04	2.04
M-C3	1.99	1.99	1.99	1.99	1.98	1.99	1.99	1.99	1.99	1.99	2.02	2.02	2.02	2.02	2.01
M-C4	1.99	1.99	1.99	1.99	1.99	1.99	1.99	1.99	1.99	1.99	2.02	2.02	2.02	2.02	2.01
Au-C5	2.03	2.03	2.03	2.04	2.04	2.03	2.03	2.03	2.03	2.03	2.03	2.03	2.03	2.04	2.04
Au-C6	2.03	2.03	2.03	2.04	2.04	2.03	2.03	2.03	2.03	2.03	2.03	2.03	2.03	2.04	2.04
Au1M2Au3	180.0	175.1	180.0	172.4	180.0	180.0	170.9	180.0	175.8	180.0	180.0	174.2	180.0	171.0	180.0
λ	457	444.00	420.00	418.00	412.00	483	476	437	440	443	417	408	383	378	372
Δλ		-13	-37	-39	-45		-7	-46	-43	-40		-9	-34	-39	-45
							d0(S_0)								
	I	I-S3	I-2S3	I-3S3	I-4S3	II	II-S3	II-2S3	II-3S3	II-4S3	III	III-S3	III-2S3	III-3S3	III-4S3
Au1-M2	3.12	3.52	3.49	3.42	3.21	3.15	3.55	3.44	3.57	3.22	3.06	3.56	3.47	3.41	3.20
M2-Au3	3.11	3.52	3.49	3.42	3.21	3.15	3.55	3.44	3.57	3.22	3.06	3.56	3.47	3.41	3.20
Au-C1	2.04	2.04	2.04	2.04	2.04	2.04	2.04	2.04	2.04	2.05	2.04	2.04	2.04	2.04	2.04
Au-C2	2.04	2.04	2.04	2.04	2.04	2.04	2.04	2.04	2.04	2.05	2.04	2.04	2.04	2.04	2.04
M-C3	2.00	1.99	1.99	1.99	1.99	2.00	1.99	1.99	1.99	1.99	2.05	2.05	2.04	2.04	2.05
M-C4	2.00	1.99	1.99	1.99	1.99	2.00	1.99	1.99	1.99	1.99	2.05	2.04	2.04	2.04	2.05
Au-C5	2.04	2.04	2.04	2.04	2.04	2.04	2.04	2.04	2.04	2.05	2.04	2.04	2.04	2.04	2.04
Au-C6	2.04	2.04	2.04	2.04	2.04	2.04	2.04	2.04	2.04	2.05	2.04	2.04	2.04	2.04	2.04
Au1M2Au3	179.98	171.28	179.98	158.67	180.00	180.00	153.10	180.00	149.70	179.99	180.00	168.10	179.98	157.96	180.00
							d1(T_1)								
Au1-M2	2.77	2.77	2.84	2.85	2.80	2.76	2.76	2.84	2.86	2.86	2.76	2.77	2.85	2.86	2.80
M2-Au3	2.77	2.79	2.84	2.85	2.80	2.75	2.79	2.83	2.85	2.86	2.76	2.80	2.85	2.86	2.80
Au-C1	2.03	2.03	2.03	2.03	2.03	2.03	2.03	2.03	2.04	2.04	2.03	2.02	2.03	2.03	2.03
Au-C2	2.03	2.03	2.03	2.03	2.03	2.03	2.03	2.03	2.03	2.04	2.03	2.02	2.03	2.03	2.03
M-C3	1.99	1.99	1.99	1.99	1.99	1.99	1.99	1.99	1.99	1.98	2.02	2.03	2.02	2.02	2.02
M-C4	1.99	1.99	1.99	1.99	1.99	1.99	1.99	1.99	1.99	1.98	2.02	2.03	2.02	2.02	2.02
Au-C5	2.03	2.03	2.03	2.03	2.03	2.03	2.03	2.03	2.04	2.04	2.03	2.03	2.03	2.03	2.03
Au-C6	2.03	2.03	2.03	2.03	2.03	2.03	2.03	2.03	2.03	2.04	2.03	2.04	2.03	2.03	2.03
Au1M2Au3	179.99	171.00	179.99	174.26	180.00	179.98	169.80	180.00	173.45	179.99	179.99	163.84	179.99	174.03	180.00
λ	457	450	421	419	435	483	475	444	441	444	417	429	383	380	391
Δλ		-7	-36	-38	-22		-8	-39	-43	-39		12	-34	-37	-26

6. Table 4S

Table 4S. Selected GKS-EDA results of **X**···(**S*n***)$_x$ (kcal/mol).

		II								I				III			
		S1	(S1)2	(S1)3	(S1)4	S2	(S2)2	(S2)3	(S2)4	S2	(S2)2	(S2)3	(S2)4	S2	(S2)2	(S2)3	(S2)4
E^{CP1}	E_{es}	-17.1	-15.8	-12.4	-11.7	-17.0	-15.6	-12.1	-11.5	-16.5	-15.6	-11.9	-11.4	-17.5	-16.2	-12.4	-11.8
	E_{ex}	-36.1	-34.3	-26.2	-24.5	-36.9	-35.1	-26.3	-24.8	-32.9	-31.3	-24.7	-23.3	-34.9	-32.7	-25.8	-24.1
	E_{rep}	61.0	57.5	43.5	40.8	62.0	58.6	43.7	41.1	55.1	52.4	41.3	38.9	58.6	54.7	43.2	40.2
	E_{pol}	-11.2	-10.4	-7.5	-7.1	-11.2	-10.4	-7.4	-7.1	-11.0	-10.1	-7.5	-7.1	-12.0	-10.9	-8.0	-7.6
	E_{disp}	-7.1	-7.5	-7.2	-6.9	-8.1	-8.6	-8.0	-7.6	-7.2	-7.0	-6.6	-6.1	-7.2	-6.9	-6.6	-6.1
	E_{corr}	-4.7	-4.6	-3.9	-3.5	-4.7	-4.7	-3.8	-3.5	-3.9	-3.9	-3.6	-3.3	-3.9	-3.9	-3.6	-3.1
	E_{tot}	-15.3	-15.1	-13.7	-13.0	-15.9	-15.8	-14.1	-13.4	-16.5	-15.5	-12.9	-12.2	-16.9	-15.8	-13.3	-12.4
E^{CP2}	E_{es}		-31.8	-29.2	-23.6		-31.5	-24.6	-23.2		-31.3	-24.0	-23.7		-32.5	-30.0	-23.7
	E_{ex}		-68.7	-61.2	-49.1		-70.4	-52.8	-49.6		-62.8	-49.4	-47.5		-65.5	-60.2	-48.1
	E_{rep}		115.1	102.2	81.7		117.5	87.2	82.3		105.0	82.3	79.0		109.6	100.9	80.5
	E_{pol}		-20.6	-18.4	-14.1		-20.6	-15.1	-14.0		-20.2	-14.8	-14.6		-21.7	-19.8	-15.0
	E_{disp}		-15.0	-14.6	-13.7		-17.2	-15.5	-15.2		-13.9	-13.0	-12.0		-13.9	-13.3	-12.2
	E_{corr}		-9.2	-8.3	-7.1		-9.4	-7.6	-7.1		-7.9	-7.1	-6.5		-7.9	-7.5	-6.3
	E_{tot}		-30.2	-29.6	-25.9		-31.7	-28.3	-26.8		-31.1	-26.0	-25.4		-31.7	-29.8	-24.8
E^{CP3}	E_{es}			-42.1	-36.7			-41.3	-36.0			-41.0	-35.6			-42.8	-36.9
	E_{ex}			-87.4	-74.7			-88.7	-75.4			-82.0	-71.0			-85.9	-73.4
	E_{rep}			145.5	123.8			147.2	124.6			136.9	118.3			143.3	122.3
	E_{pol}			-26.0	-21.9			-25.8	-21.6			-25.4	-21.7			-27.8	-23.1
	E_{disp}			-21.4	-20.4			-24.1	-22.6			-19.7	-18.1			-19.5	-18.0
	E_{corr}			-12.1	-10.6			-12.2	-10.6			-11.0	-9.8			-10.7	-9.4
	E_{tot}			-43.5	-40.4			-44.9	-41.5			-42.2	-38.0			-43.3	-38.6
E^{CP4}	E_{es}				-49.9				-48.9				-48.5				-50.3
	E_{ex}				-100.3				-101.2				-95.6				-98.8
	E_{rep}				166.1				167.1				159.0				164.3
	E_{pol}				-29.5				-29.2				-29.2				-31.2
	E_{disp}				-27.0				-29.9				-24.0				-23.9
	E_{corr}				-14.2				-14.2				-13.2				-12.6
	E_{tot}				-54.8				-56.3				-51.5				-52.4

	E_{es}	-99.7	-112.8	-124.4	-135.5	-99.4	-112.3	-123.7	-134.5	-101.5	-114.8	-125.7	-	-101.7	-114.7	-126.0	-137.7
	E_{ex}	-93.7	-126.5	-154.5	-185.9	-93.9	-127.7	-156.5	-186.9	-30.3	-125.9	-151.4	-	-89.8	-123.8	-149.3	-180.7
E^{CP}	E_{rep}	149.8	203.3	248.9	300.2	150.0	204.8	251.7	301.5	100.6	201.4	243.5	-	142.6	197.9	239.7	290.9
	E_{pol}	-26.0	-34.3	-42.3	-51.7	-25.9	-34.3	-42.4	-51.5	-30.7	-32.7	-40.2	-	-22.7	-31.8	-39.2	-47.8
	E_{disp}	-33.3	-41.5	-48.7	-55.4	-34.3	-43.8	-51.4	-58.4	-34.6	-42.0	-47.7	-	-31.6	-38.9	-44.9	-49.4
	E_{corr}	-11.4	-16.2	-19.8	-24.0	-11.4	-16.4	-20.0	-24.1	-20.5	-16.3	-19.6	-	-11.4	-16.6	-19.3	-23.5
	E_{tot}	-114.3	-128.1	-140.8	-152.2	-114.8	-129.5	-142.3	-153.8	-117.1	-130.3	-141.1	-	-114.6	-127.9	-139.0	-148.3

.

7. Figure 3S

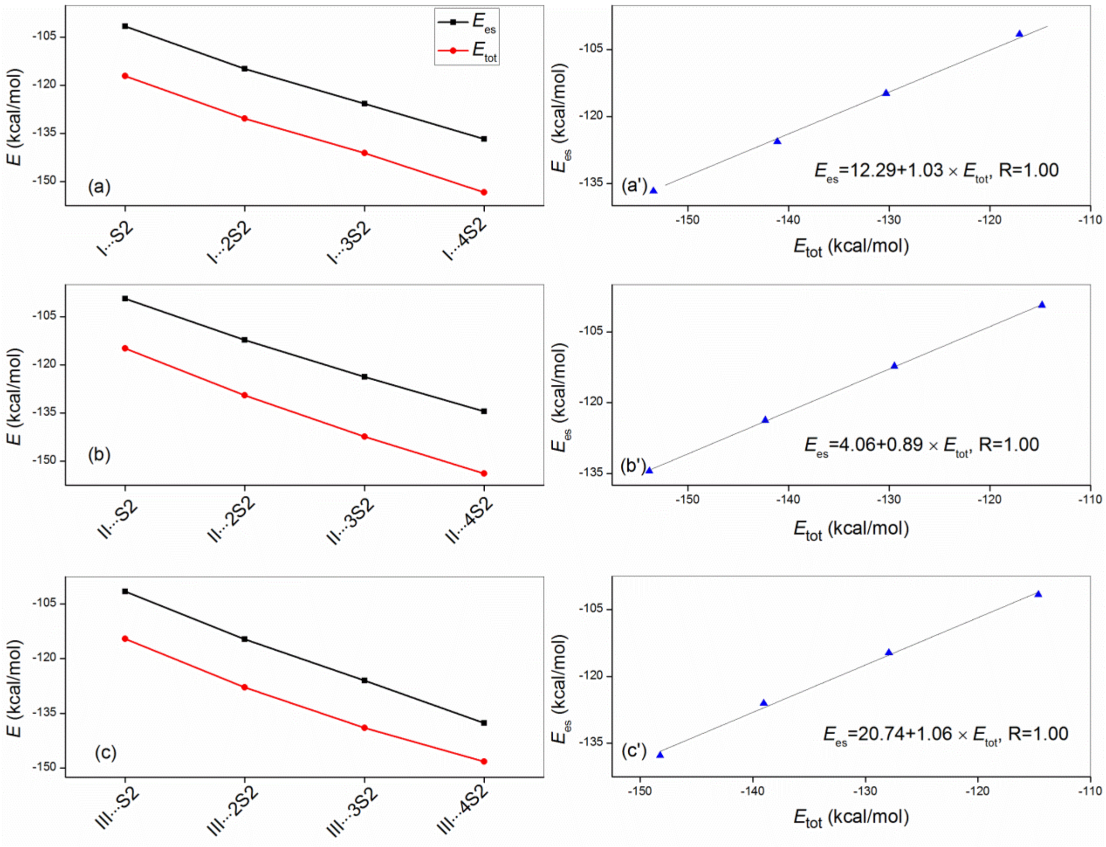

Figure 3S. Trends of interaction energies (**a**, **b**, **c**) and the relationships between the E_{es} and E_{tot} (**a'**, **b'**, **c'**).

8. Figure 4S

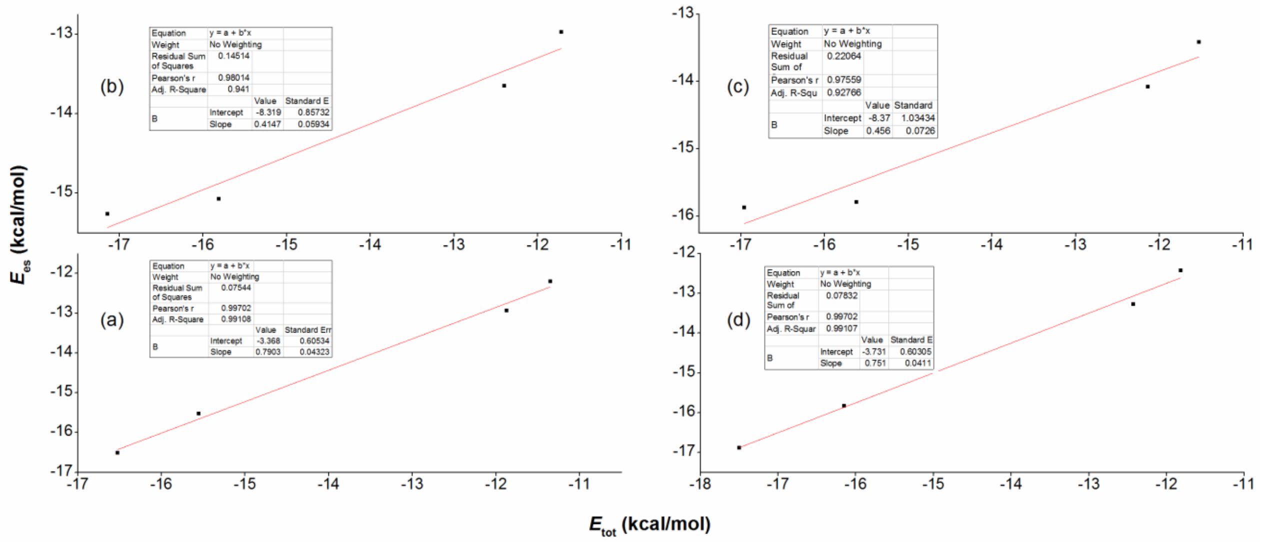

Figure 4S. The relationships between the E_{es} and E_{tot} (a) I···(**S2**)$_x$, (b) II···(**S1**)$_x$, (c) II···(**S2**)$_x$ and (d) III···(**S2**)$_x$ for the E^{CP1} of clusters.

9. Table 5S

Table 5S. Molecular Orbitals and their main consist of natural atomic orbital and the characters.

	HOMO (%)	d (%)	LUMO (%)	p (%)
I	77.23	45.29	35.71	35.34
II	74.71	42.78	26.25	26.03
III	69.50	42.74	34.30	34.10
I-S1	74.32	41.19	39.84	39.51
I-(S1)2	75.24	43.13	41.21	40.77
I-(S1)3	75.48	43.31	39.41	38.63
I-(S1)4	76.02	45.16	38.39	37.86
I-S2	75.44	42.33	39.94	38.30
I-(S2)2	75.21	42.42	41.14	40.68
I-(S2)3	75.54	43.64	39.45	38.46
I-(S2)4	76.05	45.14	38.45	37.92
II-S1	65.22	35.63	27.87	26.75
II-(S1)2	60.31	34.28	29.47	29.61
II-(S1)3	64.86	37.84	27.87	26.14
II-(S1)4	70.06	41.03	26.34	25.99
II-S2	65.05	34.85	27.93	24.02
II-(S2)2	59.99	34.61	29.41	29.03
II-(S2)3	65.38	37.80	27.82	27.43
II-(S2)4	70.12	41.63	26.31	25.96
III-S1	67.71	39.66	39.58	39.34
III-(S1)2	68.44	41.19	41.28	40.94
III-(S1)3	68.44	41.72	39.38	38.95
III-(S1)4	68.68	43.02	37.89	37.51
III-S2	67.97	40.20	39.69	39.24
III-(S2)2	68.47	41.17	41.24	40.91
III-(S2)3	68.44	41.69	39.43	39.17
III-(S2)4	68.62	43.07	38.41	38.11

10. Table 6S

Table 6S. Calculated phosphorescent emission (in nm) of the studied complexes with the TDDFT method, along with experimental values.

Complex	λ	E(eV)	Configuration		Character	Exp	
I	457	2.72	HOMO-LUMO	98%	MLCT	448	9*
II	483	2.57	HOMO-LUMO	97%	MLCT	465	18*
III	417	2.98	HOMO-LUMO	97%	MLCT	446	-29*
I-S1	450	2.76	HOMO-LUMO	98%	MLCT		-7
I-(S1)2	418.84	2.96	HOMO-LUMO	98%	MLCT		-38
I-(S1)3	417.98	2.97	HOMO-LUMO	98%	MLCT		-39
I-(S1)4	412.24	3.01	HOMO-LUMO	98%	MLCT		-45
I-S2	449.15	2.76	HOMO-LUMO	98%	MLCT		-8
I-(S2)2	420.38	2.95	HOMO-LUMO	98%	MLCT		-37
I-(S2)3	417.53	2.97	HOMO-LUMO	98%	MLCT		-39
I-(S2)4	412.2	3.01	HOMO-LUMO	98%	MLCT		-45
II-S1	478.46	2.59	HOMO-LUMO	97%	MLCT		-5
II-(S1)2	437.33	2.84	HOMO-LUMO	96%	MLCT		-46
II-(S1)3	439.21	2.82	HOMO-LUMO	96%	MLCT		-44
II-(S1)4	442.89	2.80	HOMO-LUMO	96%	MLCT		-40
II-S2	475.86	2.61	HOMO-LUMO	97%	MLCT		-7
II-(S2)2	436.66	2.84	HOMO-LUMO	96%	MLCT		-46
II-(S2)3	439.54	2.82	HOMO-LUMO	96%	MLCT		-43
II-(S2)4	443.04	2.80	HOMO-LUMO	96%	MLCT		-40
III-S1	408.98	3.03	HOMO-LUMO	97%	MLCT		-8
III-(S1)2	382.75	3.24	HOMO-LUMO	97%	MLCT		-34
III-(S1)3	379.18	3.27	HOMO-LUMO	97%	MLCT		-38
III-(S1)4	371.23	3.34	HOMO-LUMO	97%	MLCT		-46
III-S2	408.44	3.04	HOMO-LUMO	97%	MLCT		-9
III-(S2)2	383.08	3.24	HOMO-LUMO	97%	MLCT		-34
III-(S2)3	378.45	3.28	HOMO-LUMO	97%	MLCT		-39
III-(S2)4	371.68	3.34	HOMO-LUMO	97%	MLCT		-45

$$\delta^* = \lambda_{[X]}^{cal} - \lambda_{[X]}^{\exp}$$

$$\delta = \lambda_{[X\cdots(S)_n]}^{cal} - \lambda_{[X]}^{cal}$$

11. Figure 5S

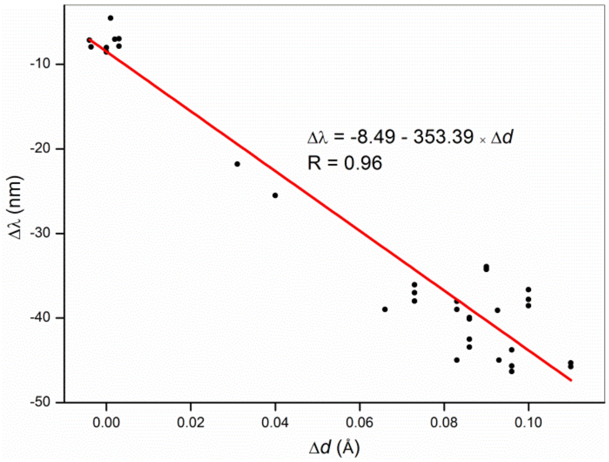

Figure 5S. The relationship between Δd and Δλ for complexes X···(S)$_x$ in T1 states.

12. Figure 6S

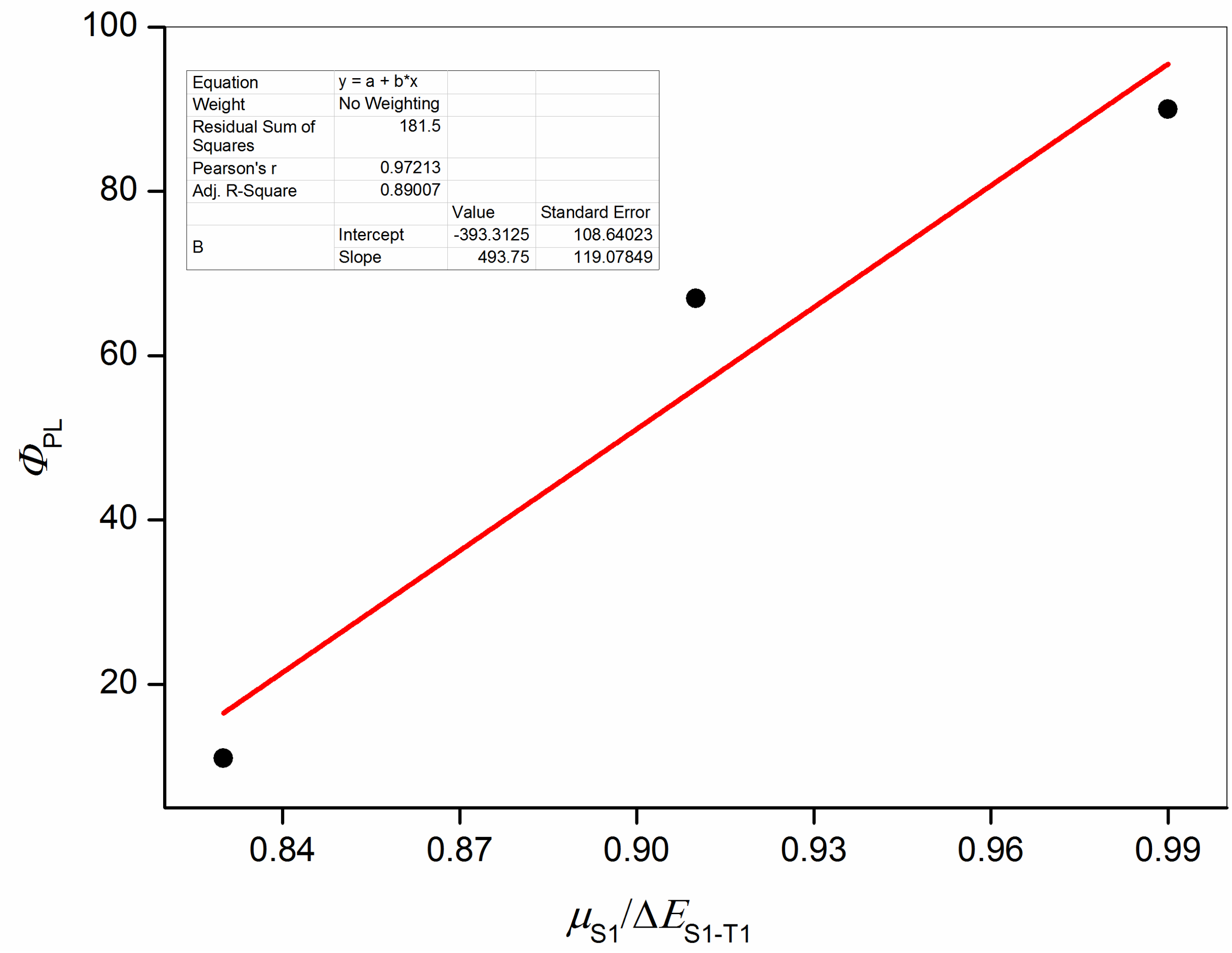

Figure 6S. The relationship betwwen $\mu_{S1}/\Delta E_{S1\text{-}T1}$ values in theory and the Φ_{PL} values in experiment.

13. Table 7S

Table 7S. The singlet-triplet splitting energy ($\Delta E_{S1\text{-}T1}$, in eV), transition electric dipole moment (μ_{S1}, in atomics units), the $\mu_{S1}/\Delta E_{S1\text{-}T1}$, and the predicted Φ_{PL} (> 50%) for complexes X···(S)$_x$.

complex	$\Delta E_{S1\text{-}T1}$	μ_{S1}	$\mu_{S1}/\Delta E_{S1\text{-}T1}$	Φ_{PL}(%)
I-S1	2.85	2.81	0.99	94.6
II-S1	2.73	2.53	0.92	63.0
II-2S1	3.01	2.82	0.94	69.2
II-3S1	3.00	2.77	0.92	62.9
I-4S1	2.96	2.77	0.94	68.4
II-4S1	2.97	2.72	0.92	59.1
I-S2	2.85	2.80	0.98	92.5
II-3S2	3.00	2.74	0.91	58.2
I-4S2	2.96	2.73	0.92	61.7
II-4S2	2.97	2.68	0.90	52.7
I-S3	2.95	2.79	0.94	73.2
II-S3	2.83	2.68	0.95	74.9
I-2S3	3.12	2.83	0.91	55.1
II-2S3	2.96	2.72	0.92	59.5

14. Table 8S

Table 9S. The E^{CP} of cluster **I** at different calculated levels (kcal/mol).

Mode	MP2			PBE0-D3		PBE0		M06-2X	
	BS1	BS3	BS4	BS3	BS4	BS3	BS4	BS3	BS4
$E^{CP}{}_{add}$	-22.5	-29.2	-30.7	-29.0	-29.0	-19.8	-19.8	-24.3	-24.3
$E^{CP}{}_{tot}$	-88.8	-102.3	-105.1	-102.5	-102.6	-85.9	-85.9	-94.9	-97.1

The binding energies with BSSE corrections of cluster **I** at different calculated levels show that the E^{CP} of the PBE0-D3/BS3 and PBE0-D3/BS4 are identical to each other, and they both close to the MP2/BS4 results and the PBE0-D3/BS3 are more financial than PBE0-D3/BS4 in time.

15. Table 9S

Table 10S. The EDA results of cluster I at different calculated levels (kcal/mol).

		E_{es}	E_{ex}	E_{rep}	E_{pol}	E_{disp}	E_{corr}	E_{tot}	$E^{CP a}$
E^{CP}_{add}	MP2/BS5[b]	-23.44	-39.6	64.73	-12.97	-15.34		-26.62	
	MP2/BS6[b]	-23.01	-39.09	64.16	-13.21	-18.1		-29.25	
	B3LYP-D3/BS5[c]	-23.29	-46.03	74.84	-15.55	-13.99	-5.56	-29.57	-30.65
	B3LYP-D3/BS6[c]	-22.87	-45.38	74.1	-15.78	-13.99	-5.46	-29.38	
E^{CP}_{tot}	MP2/BS5[b]	-95.48	-85.14	137.53	-25.19	-28.66		-96.94	
	MP2/BS6[b]	-95.11	-84.03	136.16	-25.34	-33.97		-102.29	
	B3LYP-D3/BS5[c]	-95.76	-99.47	159.8	-29.87	-26.23	-12.48	-104.0	-105.10
	B3LYP-D3/BS6[c]	-95.23	-97.97	157.93	-30.19	-26.23	-12.01	-103.69	

[a] Calculated at the MP2/BS4 with gaussian; [b]LMO-EDA; [c]GKS-EDA.

The LMO-EDA and GKS-EDA results at four calculated levels MP2/BS5, MP2/BS6, B3LYP-D3/BS5 and B3LYP-D3/BS6 show that the GKS-EDA total interaction energies (E_{tot}) at B3LYP-D3/BS5 and B3LYP-D3/BS6 levels are very close and they are also consistent with those in MP2/BS6 level with LMO-EDA scheme as well as the E^{CP} at MP2/BS4 level.

16. The S_0 .xyz files of I and its complexes

I

Au 3.113900 -0.000000 -0.000500
N 2.946500 -2.991000 0.564400
N 3.179300 -2.639800 -1.539700
C 3.107900 -2.006500 -0.348500
C 3.061100 -4.001000 -1.377600
H 3.091700 -4.686000 -2.210800
C 2.919100 -4.224100 -0.046300
H 2.814900 -5.142700 0.510300
C 2.834600 -2.769300 1.996300
H 2.125200 -1.961300 2.190100
H 2.469800 -3.684600 2.465600
C 3.270400 -1.969200 -2.826900
H 3.912100 -1.092700 -2.723200
H 3.715100 -2.654200 -3.551400
H 3.809700 -2.513200 2.419300
H 2.277500 -1.655900 -3.163300
N 2.946000 2.990900 -0.565200
N 3.180300 2.639700 1.538700
C 3.108100 2.006400 0.347500
C 3.061800 4.000800 1.376800
H 3.092800 4.685800 2.210000
C 2.918800 4.224000 0.045600
H 2.814000 5.142600 -0.510900
C 2.833200 2.769200 -1.997000
H 2.122700 1.962100 -2.190400
H 2.469400 3.684900 -2.466200
C 3.272600 1.969000 2.825900
H 3.915000 1.093100 2.721700
H 3.717100 2.654300 3.550100
H 3.807700 2.511800 -2.420300
H 2.280100 1.654900 3.162800
N -0.006900 0.384600 3.142500
C -0.003100 0.237500 1.982300
N -0.010000 -0.385700 -3.141400
C -0.004800 -0.238000 -1.981300
Au -0.002300 -0.000100 0.000500
Au -3.116700 0.000200 0.000200
N -3.114300 -2.574500 1.639600
N -3.003100 -3.012300 -0.458200
C -3.107600 -1.991200 0.421000
C -2.944400 -4.219400 0.199900
H -2.872300 -5.160100 -0.323800
C -3.012800 -3.942100 1.527200
H -3.008000 -4.593500 2.387500
C -3.160200 -1.843800 2.896600
H -2.221300 -1.306700 3.058600
H -3.988800 -1.133200 2.874000
C -2.938500 -2.844500 -1.901400
H -3.857200 -2.377100 -2.263200
H -2.079800 -2.223800 -2.170700
H -3.322300 -2.554300 3.708800
H -2.833300 -3.827800 -2.362700
N -3.115200 2.574600 -1.639300
N -3.001300 3.012800 0.458300
C -3.107100 1.991600 -0.420600
C -2.943300 4.219700 -0.200100
H -2.870400 5.160500 0.323400
C -3.013400 3.942300 -1.527300
H -3.009400 4.593500 -2.387700
C -3.162400 1.843600 -2.896200
H -2.224100 1.305700 -3.058500
H -3.991700 1.133900 -2.872900
C -2.935800 2.845200 1.901500
H -3.857400 2.384800 2.265100
H -2.081200 2.218400 2.169600
H -3.324300 2.554200 -3.708400
H -2.822500 3.827900 2.362000

I...S1

Au 3.521600 -0.131500 -0.027800
N 3.445700 -2.960900 1.122000
N 3.338300 -3.024400 -1.018200
C 3.440100 -2.170500 0.023200
C 3.263000 -4.325200 -0.581300
H 3.143600 -5.154300 -1.261000
C 3.339300 -4.287800 0.771300
H 3.323600 -5.079900 1.504000
C 3.558900 -2.491700 2.488500
H 3.417200 -1.410200 2.496900
H 2.783700 -2.957000 3.102400
C 3.191600 -2.637200 -2.408100
H 3.453300 -1.582500 -2.503900
H 3.865600 -3.233500 -3.028200
H 4.544300 -2.734800 2.897100
H 2.153900 -2.792000 -2.715000
N 2.977400 2.766200 -0.852400
N 4.335400 2.695200 0.803800
C 3.630100 1.906700 -0.040600
C 4.114900 4.026800 0.531000
H 4.576700 4.817000 1.102900
C 3.255000 4.070000 -0.516100
H 2.803100 4.899600 -1.036100
C 2.113900 2.391900 -1.956700
H 1.687600 1.408100 -1.753200
H 1.312500 3.130400 -2.024900
C 5.171500 2.221300 1.889100
H 5.344300 1.152900 1.752800
H 6.130800 2.744600 1.870300
H 2.683600 2.363400 -2.890600
H 4.680300 2.392100 2.851400
N -0.002500 2.533800 1.086100
C -0.001300 1.400900 0.802100
N 0.002900 -3.314400 -1.265400
C 0.002100 -2.281200 -0.718900
Au 0.000600 -0.467700 0.107800
Au -3.521500 -0.135500 -0.027600
N -3.439200 -2.963200 1.125800
N -3.333200 -3.029300 -1.014400
C -3.435900 -2.174300 0.025900
C -3.255100 -4.329400 -0.575900

H -3.134500 -5.159200 -1.254500
C -3.330500 -4.290400 0.776700
H -3.312800 -5.081500 1.510500
C -3.552300 -2.492400 2.491700
H -3.412000 -1.410700 2.498700
H -4.537000 -2.736400 2.901300
C -3.188500 -2.643800 -2.405000
H -3.451700 -1.589500 -2.501800
H -2.150800 -2.797500 -2.712700
H -2.776100 -2.955900 3.105800
H -3.862300 -3.241800 -3.023600
N -2.981600 2.762200 -0.854800
N -4.341800 2.690600 0.799500
C -3.633900 1.902500 -0.043000
C -4.123400 4.022400 0.525500
H -4.587500 4.812300 1.095700
C -3.262100 4.065900 -0.520500
H -2.811200 4.895700 -1.041000
C -2.115900 2.388100 -1.957500
H -1.688400 1.405200 -1.752500
H -2.684100 2.357900 -2.892300
C -5.178800 2.216600 1.884100
H -5.347800 1.147300 1.749800
H -4.690200 2.391000 2.847100
H -1.315500 3.127800 -2.025100
H -6.139800 2.736700 1.862000
O -0.003300 4.675200 -0.759600
H -0.003300 4.017000 -0.030200
C -0.005500 5.960600 -0.182300
H 0.882800 6.139900 0.441300
H -0.005700 6.692600 -0.995800
H -0.895500 6.137600 0.439700

I···2S1
Au -3.455700 -0.000900 0.000100
N -3.009100 2.942600 0.723300
N -3.982500 2.769100 -1.178600
C -3.487900 2.035100 -0.155000
C -3.804400 4.114300 -0.947900
H -4.118400 4.867300 -1.654200
C -3.188000 4.222200 0.254600
H -2.849000 5.083800 0.807700
C -2.372000 2.639100 1.991800
H -1.993800 1.616000 1.958500
H -1.540500 3.334000 2.132800
C -4.571900 2.227900 -2.387500
H -4.724700 1.156800 -2.248400
H -5.535700 2.707400 -2.577100
H -3.090400 2.738900 2.811200
H -3.905900 2.392400 -3.239300
N -3.006800 -2.944100 -0.723300
N -3.979800 -2.771300 1.178900
C -3.486000 -2.036900 0.155200
C -3.800800 -4.116300 0.948200
H -4.114100 -4.869600 1.654600
C -3.184600 -4.223900 -0.254500
H -2.845300 -5.085200 -0.807700
C -2.370300 -2.640200 -1.991900
H -1.993500 -1.616600 -1.959100
H -1.537800 -3.334000 -2.132700
C -4.569000 -2.230500 2.388100
H -4.723900 -1.159800 2.248500
H -5.531700 -2.711700 2.578900
H -3.088600 -2.741400 -2.811300
H -3.901900 -2.393200 3.239300
N 0.001600 -3.006900 0.967200
C 0.001000 -1.880300 0.658500
N -0.001600 3.006600 -0.967300
C -0.001000 1.880000 -0.658700
Au 0.000000 -0.000200 -0.000200
Au 3.455700 0.001000 0.000200
N 3.006400 2.944100 0.723600
N 3.981100 2.771600 -1.177800
C 3.486400 2.037000 -0.154600
C 3.801800 4.116600 -0.947000
H 4.115700 4.869900 -1.653000
C 3.184600 4.223900 0.255200
H 2.844700 5.085100 0.808200
C 2.369100 2.640000 1.991800
H 1.991300 1.616700 1.958200
H 3.087100 2.740000 2.811500
C 4.571200 2.230900 -2.386600
H 4.726600 1.160300 -2.246800
H 3.904600 2.393300 -3.238200
H 1.537200 3.334400 2.132700
H 5.533900 2.712500 -2.576800
N 3.009000 -2.942300 -0.723900
N 3.981700 -2.769400 1.178500
C 3.487500 -2.035000 0.154900
C 3.803700 -4.114500 0.947400
H 4.117400 -4.867700 1.653600
C 3.187700 -4.222100 -0.255400
H 2.848900 -5.083500 -0.808800
C 2.372300 -2.638600 -1.992500
H 1.995000 -1.615100 -1.959400
H 3.090700 -2.739100 -2.811800
C 4.570700 -2.228500 2.387800
H 4.722300 -1.157100 2.249400
H 3.905000 -2.394300 3.239500
H 1.540300 -3.332800 -2.133400
H 5.535100 -2.707100 2.576800
O 0.001900 -4.907600 -1.136500
H 0.001900 -4.364200 -0.320000
C 0.002000 -6.266800 -0.767700
H -0.885900 -6.543900 -0.179400
H 0.001700 -6.860900 -1.686100
H 0.890100 -6.543900 -0.179900
O -0.002100 4.908200 1.135600
H -0.002100 4.364200 0.319500
C -0.002200 6.267100 0.765800
H -0.001700 6.861800 1.683800
H 0.885500 6.543700 0.177100
H -0.890500 6.543800 0.178100

I-3S1
Au -3.331000 0.312000 0.043900

N	-3.419300	-2.620700	-0.840300
N	-3.562100	-2.468200	1.295100
C	-3.442400	-1.724400	0.172300
C	-3.604800	-3.808500	0.991200
H	-3.698100	-4.568300	1.751800
C	-3.515500	-3.904400	-0.358300
H	-3.517300	-4.764800	-1.009700
C	-3.205000	-2.308200	-2.241700
H	-3.072900	-1.229700	-2.337600
H	-2.300600	-2.816700	-2.590900
C	-3.524800	-1.956100	2.653800
H	-3.625200	-0.870400	2.615900
H	-4.356600	-2.373100	3.227600
H	-4.068100	-2.623900	-2.834800
H	-2.569900	-2.228800	3.115300
N	-2.697500	3.213800	0.777900
N	-3.800000	3.127200	-1.057900
C	-3.281000	2.349100	-0.080100
C	-3.533900	4.456600	-0.819600
H	-3.849300	5.238000	-1.493700
C	-2.835700	4.509600	0.341400
H	-2.409200	5.339800	0.881700
C	-2.011000	2.850000	2.003700
H	-1.605500	1.842600	1.893700
H	-1.195200	3.559500	2.161100
C	-4.490800	2.640100	-2.235700
H	-4.735700	1.588000	-2.083900
H	-5.414500	3.205700	-2.382400
H	-2.703400	2.877100	2.850700
H	-3.853700	2.742500	-3.118800
N	0.296300	3.374100	-0.985500
C	0.195600	2.215800	-0.878900
N	-0.243700	-2.811600	0.148400
C	-0.144800	-1.674900	-0.110400
Au	0.024600	0.262300	-0.528900
Au	3.341100	-0.149700	0.079700
N	2.956700	-2.985300	-1.006400
N	3.051500	-3.006500	1.136800
C	3.101700	-2.178400	0.069000
C	2.869800	-4.309300	0.736000
H	2.799300	-5.123700	1.440500
C	2.810800	-4.295600	-0.618700
H	2.677300	-5.093000	-1.333000
C	2.880300	-2.550400	-2.389500
H	2.811500	-1.461700	-2.406500
H	3.772000	-2.868900	-2.937700
C	3.048800	-2.586600	2.527100
H	3.361900	-1.542500	2.572600
H	2.039700	-2.690900	2.940400
H	1.982900	-2.981700	-2.842000
H	3.754800	-3.196800	3.096600
N	3.133100	2.778300	0.949400
N	4.348200	2.598100	-0.806800
C	3.634700	1.870900	0.083800
C	4.282500	3.940400	-0.508300
H	4.773200	4.689900	-1.110000
C	3.512900	4.052800	0.602100
H	3.186600	4.915600	1.161000
C	2.303900	2.476100	2.101400
H	1.765000	1.546000	1.912100
H	2.922300	2.367000	2.997600
C	5.044500	2.060000	-1.959000
H	5.091000	0.974700	-1.860100
H	4.512800	2.319700	-2.878900
H	1.591200	3.293600	2.232400
H	6.060600	2.460800	-1.997100
O	0.366600	5.095000	1.252200
H	0.357600	4.615800	0.395500
C	0.493700	6.473700	0.988400
H	-0.343600	6.866200	0.392200
H	1.425700	6.714800	0.455900
H	0.503500	6.997100	1.948800
O	-0.132100	-4.183600	-2.403200
H	-0.163300	-3.853800	-1.485300
C	-0.362400	-5.571800	-2.390900
H	0.398300	-6.123300	-1.817000
H	-1.348300	-5.834200	-1.977500
H	-0.326600	-5.921700	-3.426000
C	-0.415100	-4.705900	3.284700
O	-0.367100	-3.331700	2.984100
H	0.473100	-5.248600	2.925500
H	-1.303600	-5.200300	2.863100
H	-0.345200	-3.234900	2.012200
H	-0.454500	-4.805800	4.372800

I-4S1

Au	-3.251900	0.000000	0.000000
N	-3.281600	2.868300	1.072700
N	-3.281600	2.868300	-1.072700
C	-3.279600	2.044200	0.000000
C	-3.277300	4.185000	-0.677800
H	-3.277100	4.998300	-1.387100
C	-3.277300	4.185000	0.677800
H	-3.277100	4.998300	1.387100
C	-3.183900	2.452200	2.460400
H	-3.248000	1.364200	2.498400
H	-2.223400	2.780700	2.871500
C	-3.183900	2.452200	-2.460400
H	-3.248000	1.364200	-2.498400
H	-4.009000	2.880900	-3.035800
H	-4.009000	2.880900	3.035800
H	-2.223400	2.780700	-2.871500
N	-3.281600	-2.868300	-1.072700
N	-3.281600	-2.868300	1.072700
C	-3.279600	-2.044200	0.000000
C	-3.277300	-4.185000	0.677800
H	-3.277100	-4.998300	1.387100
C	-3.277300	-4.185000	-0.677800
H	-3.277100	-4.998300	-1.387100
C	-3.183900	-2.452200	-2.460400
H	-3.248000	-1.364200	-2.498400
H	-2.223400	-2.780700	-2.871500
C	-3.183900	-2.452200	2.460400
H	-3.248000	-1.364200	2.498400
H	-4.009000	-2.880900	3.035800

H	-4.009000	-2.880900	-3.035800
H	-2.223400	-2.780700	2.871500
N	0.000000	-3.160200	0.000000
C	0.000000	-1.990000	0.000000
N	0.000000	3.160200	0.000000
C	0.000000	1.990000	0.000000
Au	0.000000	0.000000	0.000000
Au	3.251900	0.000000	0.000000
N	3.281600	2.868300	1.072700
N	3.281600	2.868300	-1.072700
C	3.279600	2.044200	0.000000
C	3.277300	4.185000	-0.677800
H	3.277100	4.998300	-1.387100
C	3.277300	4.185000	0.677800
H	3.277100	4.998300	1.387100
C	3.183900	2.452200	2.460400
H	3.248000	1.364200	2.498400
H	4.009000	2.880900	3.035800
C	3.183900	2.452200	-2.460400
H	3.248000	1.364200	-2.498400
H	2.223400	2.780700	-2.871500
H	2.223400	2.780700	2.871500
H	4.009000	2.880900	-3.035800
N	3.281600	-2.868300	-1.072700
N	3.281600	-2.868300	1.072700
C	3.279600	-2.044200	0.000000
C	3.277300	-4.185000	0.677800
H	3.277100	-4.998300	1.387100
C	3.277300	-4.185000	-0.677800
H	3.277100	-4.998300	-1.387100
C	3.183900	-2.452200	-2.460400
H	3.248000	-1.364200	-2.498400
H	4.009000	-2.880900	-3.035800
C	3.183900	-2.452200	2.460400
H	3.248000	-1.364200	2.498400
H	2.223400	-2.780700	2.871500
H	2.223400	-2.780700	-2.871500
H	4.009000	-2.880900	3.035800
O	0.000000	-3.844500	-2.832900
H	0.000000	-3.724900	-1.864700
C	0.000000	-5.225600	-3.107800
H	-0.889600	-5.735700	-2.707700
H	0.889600	-5.735700	-2.707700
H	0.000000	-5.345700	-4.194300
O	0.000000	3.844500	2.832900
H	0.000000	3.724900	1.864700
C	0.000000	5.225600	3.107800
H	0.889600	5.735700	2.707700
H	-0.889600	5.735700	2.707700
H	0.000000	5.345700	4.194300
C	0.000000	5.225600	-3.107800
O	0.000000	3.844500	-2.832900
H	0.889600	5.735700	-2.707700
H	-0.889600	5.735700	-2.707700
H	0.000000	3.724900	-1.864700
H	0.000000	5.345700	-4.194300
C	0.000000	-5.225600	3.107800
O	0.000000	-3.844500	2.832900
H	0.889600	-5.735700	2.707700
H	-0.889600	-5.735700	2.707700
H	0.000000	-3.724900	1.864700
H	0.000000	-5.345700	4.194300

I-S2

N	-0.000200	-3.317400	-1.472900
C	-0.000100	-2.314700	-0.872100
N	0.000100	2.392700	1.198700
C	0.000000	1.276800	0.852900
Au	-0.000100	-0.550600	0.055900
Au	3.486600	-0.257000	0.009600
N	2.988800	2.671300	-0.736400
N	4.296700	2.533800	0.955700
C	3.609300	1.779900	0.066100
C	4.097400	3.875600	0.719200
C	3.269100	3.960700	-0.350500
C	2.148400	2.341500	-1.872600
C	5.094000	2.017100	2.050600
N	3.357600	-3.122500	1.063000
N	3.333900	-3.116200	-1.080700
C	3.397000	-2.296900	-0.009000
C	3.238000	-4.430100	-0.689600
C	3.261000	-4.436900	0.665500
C	3.410600	-2.697700	2.447400
C	3.245700	-2.683500	-2.462300
H	4.549600	4.642400	1.329200
H	2.843600	4.811700	-0.858000
H	1.722000	1.348800	-1.718900
H	1.345500	3.079800	-1.924900
H	2.736500	2.353400	-2.795400
H	5.254900	0.950400	1.888700
H	6.060900	2.525900	2.074800
H	4.576700	2.167900	3.002600
H	3.146000	-5.236600	-1.400200
H	3.215700	-5.252400	1.370900
H	3.326000	-1.610700	2.479200
H	2.576100	-3.136300	3.000600
H	4.355300	-3.004400	2.906000
H	3.514900	-1.627600	-2.512200
H	3.942500	-3.262600	-3.073400
H	2.220800	-2.822400	-2.816500
Au	-3.486600	-0.256700	0.009600
N	-2.988600	2.671500	-0.736200
N	-4.296400	2.534100	0.955900
C	-3.609100	1.780200	0.066200
C	-4.096900	3.875900	0.719500
C	-3.268700	3.960900	-0.350300
C	-2.148300	2.341800	-1.872600
C	-5.093600	2.017400	2.050800
N	-3.357900	-3.122300	1.062900
N	-3.334200	-3.115900	-1.080900
C	-3.397300	-2.296700	-0.009100
C	-3.238400	-4.429900	-0.689800
C	-3.261500	-4.436800	0.665300
C	-3.410900	-2.697600	2.447200
C	-3.245900	-2.683100	-2.462400

H	-4.549000	4.642700	1.329500
H	-2.843200	4.812000	-0.857700
H	-1.721900	1.348900	-1.718900
H	-2.736400	2.353700	-2.795300
H	-1.345300	3.079900	-1.924900
H	-5.254700	0.950700	1.888800
H	-4.576300	2.168100	3.002800
H	-6.060500	2.526400	2.075100
H	-3.146500	-5.236300	-1.400500
H	-3.216200	-5.252200	1.370600
H	-3.326200	-1.610600	2.479100
H	-4.355600	-3.004200	2.905900
H	-2.576400	-3.136200	3.000400
H	-3.515100	-1.627200	-2.512300
H	-2.221100	-2.822000	-2.816600
H	-3.942700	-3.262100	-3.073600
C	0.000300	5.851200	-0.052700
C	0.000400	6.883900	-1.159300
O	0.000200	4.561200	-0.632700
H	0.884200	5.988400	0.590400
H	-0.883600	5.988600	0.590400
H	-0.884100	6.772100	-1.796300
H	0.000500	7.897600	-0.746000
H	0.884800	6.771900	-1.796300
H	0.000200	3.898400	0.092400

I-2S2

Au	3.446300	0.206800	-0.001700
N	2.813200	3.161200	-0.513900
N	3.793700	2.912200	1.375900
C	3.347700	2.224400	0.299200
C	3.531400	4.256600	1.241700
C	2.910500	4.412200	0.046800
C	2.196000	2.910000	-1.803400
C	4.413700	2.323300	2.546600
N	3.183400	-2.800800	0.513600
N	4.153400	-2.437200	-1.363000
C	3.608100	-1.807200	-0.296800
C	4.059900	-3.803600	-1.225500
C	3.445500	-4.031700	-0.038800
C	2.523900	-2.624600	1.794600
C	4.714800	-1.778900	-2.526300
H	3.795400	4.974800	2.002600
H	2.516800	5.288100	-0.443800
H	1.863100	1.871200	-1.836400
H	1.334700	3.574600	-1.906000
H	2.912200	3.092500	-2.610400
H	4.627600	1.274900	2.334300
H	5.348500	2.842900	2.772600
H	3.739400	2.390000	3.405000
H	4.424300	-4.485600	-1.978200
H	3.159200	-4.948600	0.451400
H	2.063600	-1.635500	1.819300
H	1.750800	-3.391000	1.890000
H	3.247900	-2.713800	2.610400
H	4.798700	-0.712200	-2.313600
H	5.708300	-2.182800	-2.737600
H	4.066100	-1.926400	-3.394300
Au	-3.445900	-0.206500	0.001500
N	-3.183600	2.801200	-0.513900
N	-4.153300	2.437500	1.362800
C	-3.607900	1.807600	0.296600
C	-4.060100	3.803900	1.225300
C	-3.445800	4.032000	0.038500
C	-2.524100	2.625200	-1.794900
C	-4.714400	1.779000	2.526200
N	-2.813400	-3.161000	0.514000
N	-3.793300	-2.911800	-1.376100
C	-3.347400	-2.224100	-0.299300
C	-3.531200	-4.256300	-1.241900
C	-2.910700	-4.412000	-0.046700
C	-2.196500	-2.909900	1.803700
C	-4.412600	-2.322800	-2.547000
H	-4.424500	4.485900	1.978000
H	-3.159800	4.949000	-0.451700
H	-2.064100	1.635800	-1.819900
H	-3.247900	2.714900	-2.610700
H	-1.750600	3.391300	-1.890000
H	-4.798400	0.712400	2.313400
H	-4.065400	1.926500	3.394000
H	-5.707800	2.183000	2.737800
H	-3.795100	-4.974400	-2.002900
H	-2.517400	-5.288000	0.444000
H	-1.863800	-1.871100	1.837000
H	-2.912800	-3.092700	2.610400
H	-1.335100	-3.574400	1.906300
H	-4.627400	-1.274600	-2.334500
H	-3.737500	-2.388700	-3.404900
H	-5.346900	-2.842900	-2.774100
N	0.179700	-2.927400	-1.175100
C	0.112400	-1.827400	-0.787800
Au	-0.000100	-0.000600	-0.000300
N	-0.179700	2.926200	1.174800
C	-0.112500	1.826200	0.787300
C	0.493600	-7.227200	1.531900
C	0.395700	-6.308600	0.333200
O	0.305200	-4.973200	0.789700
H	0.562700	-8.273000	1.216100
H	1.378800	-6.988200	2.131200
H	-0.386000	-7.118200	2.175300
H	1.275900	-6.441400	-0.316400
H	-0.484200	-6.575500	-0.274600
H	0.265000	-4.376800	0.011700
C	-0.396300	6.308200	-0.330900
C	-0.495900	7.227800	-1.528700
O	-0.305800	4.973300	-0.788700
H	0.484100	6.574900	0.276200
H	-1.275900	6.440000	0.319700
H	-1.381700	6.989000	-2.127300
H	-0.565100	8.273400	-1.211900
H	0.383000	7.119800	-2.173300
H	-0.265300	4.376200	-0.011200

I-3S2

Au	-0.334500	3.336000	-0.025200
N	-3.254700	2.795800	-0.780700
N	-3.045200	2.988700	1.345800
C	-2.341300	3.018000	0.191700
C	-4.375700	2.743400	1.100800
H	-5.104800	2.679500	1.893800
C	-4.507800	2.622400	-0.243100
H	-5.375600	2.433400	-0.856100
C	-2.963600	2.639200	-2.194200
H	-1.886300	2.735100	-2.336600
H	-3.285400	1.645200	-2.521200
C	-2.479800	3.075700	2.680700
H	-1.439300	3.392300	2.594300
H	-3.031700	3.814900	3.267500
H	-3.477800	3.411900	-2.773000
H	-2.534000	2.092300	3.159400
N	2.664600	3.313500	0.575000
N	2.271600	4.379700	-1.242200
C	1.661100	3.709500	-0.237600
C	3.636800	4.390700	-1.065400
H	4.306000	4.860900	-1.769400
C	3.883700	3.715000	0.083800
H	4.807100	3.465200	0.581700
C	2.503900	2.565300	1.808100
H	1.606100	1.949200	1.731200
H	3.379400	1.924100	1.934500
C	1.600700	4.960800	-2.388400
H	0.528500	4.982200	-2.188600
H	1.956500	5.982100	-2.545900
H	2.409800	3.247800	2.658300
H	1.792900	4.363100	-3.284000
N	3.370400	0.400800	-1.184600
C	2.221900	0.262000	-1.027100
N	-2.738800	-0.335000	0.222100
C	-1.618300	-0.199700	-0.085800
Au	0.292200	0.030100	-0.590300
Au	0.601900	-3.295000	0.036500
N	-2.299700	-3.514800	-0.910900
N	-2.201300	-3.577500	1.231300
C	-1.431000	-3.475500	0.124700
C	-3.530900	-3.670500	0.893700
H	-4.309100	-3.753700	1.636700
C	-3.592700	-3.631600	-0.460400
H	-4.433400	-3.673100	-1.135200
C	-1.954200	-3.372800	-2.313900
H	-0.904500	-3.084100	-2.384000
H	-2.108900	-4.319100	-2.840800
C	-1.726400	-3.467900	2.599300
H	-0.638500	-3.549100	2.594000
H	-2.025700	-2.499500	3.014800
H	-2.579900	-2.588700	-2.749800
H	-2.142800	-4.281400	3.199400
N	3.462300	-2.479900	0.761000
N	3.452100	-3.720200	-0.986800
C	2.637300	-3.165800	-0.059500
C	4.764600	-3.375200	-0.755400
H	5.569000	-3.704300	-1.394900
C	4.769200	-2.589900	0.349700
H	5.571900	-2.087000	0.865400
C	3.051300	-1.721800	1.928200
H	2.024100	-1.382600	1.782900
H	3.109700	-2.344000	2.826500
C	3.014200	-4.521600	-2.112800
H	1.969200	-4.794100	-1.958900
H	3.109700	-3.953700	-3.042600
H	3.713300	-0.857900	2.024100
H	3.616800	-5.431400	-2.175300
O	5.171600	0.711000	0.975700
H	4.670200	0.612900	0.137300
C	6.543700	0.870900	0.667500
H	6.700300	1.756800	0.031400
H	6.919000	0.005100	0.098800
C	7.318800	1.017700	1.958900
H	7.188700	0.132900	2.591200
H	8.387500	1.143800	1.758200
H	6.968800	1.888700	2.523200
O	-4.158100	-0.748900	-2.281400
H	-3.812900	-0.642500	-1.374700
C	-5.568900	-0.814100	-2.230400
H	-5.902700	-1.652900	-1.597300
H	-5.989700	0.102200	-1.784100
C	-6.096100	-0.988400	-3.637700
H	-7.189600	-1.041300	-3.639900
H	-5.787600	-0.149400	-4.269700
H	-5.704400	-1.908600	-4.083600
C	-4.515800	-0.529800	3.450000
O	-3.172700	-0.293200	3.080000
H	-4.861800	-1.506300	3.071700
H	-5.184100	0.230800	3.013400
H	-3.116000	-0.303900	2.104400
C	-4.616400	-0.499700	4.959300
H	-4.298600	0.473900	5.347000
H	-5.645600	-0.682400	5.284700
H	-3.971600	-1.265400	5.402800

I-4S2

C	4.185000	3.265400	0.677800
C	2.451800	3.180100	2.460200
C	2.451800	3.180100	-2.460200
N	-2.868300	3.275500	-1.072700
N	-2.868300	3.275500	1.072700
C	-2.044200	3.276800	0.000000
C	-4.185000	3.265400	0.677800
C	-4.185000	3.265400	-0.677800
C	-2.451800	3.180100	-2.460200
C	-2.451800	3.180100	2.460200
Au	0.000000	3.251800	0.000000
N	2.868300	3.275500	1.072700
N	2.868300	3.275500	-1.072700
C	2.044200	3.276800	0.000000
C	4.185000	3.265400	-0.677800
H	4.998300	3.259900	1.387100
H	1.363700	3.243000	2.497600
H	2.781400	2.220800	2.873200

H	2.879100	4.007000	3.034200
H	1.363700	3.243000	-2.497600
H	2.879100	4.007000	-3.034200
H	2.781400	2.220800	-2.873200
H	-4.998300	3.259900	1.387100
H	-4.998300	3.259900	-1.387100
H	-1.363700	3.243000	-2.497600
H	-2.781400	2.220800	-2.873200
H	-2.879100	4.007000	-3.034200
H	-1.363700	3.243000	2.497600
H	-2.879100	4.007000	3.034200
H	-2.781400	2.220800	2.873200
H	4.998300	3.259900	-1.387100
N	-3.160300	0.000000	0.000000
C	-1.990200	0.000000	0.000000
N	3.160300	0.000000	0.000000
C	1.990200	0.000000	0.000000
Au	0.000000	0.000000	0.000000
Au	0.000000	-3.251800	0.000000
N	2.868300	-3.275500	1.072700
N	2.868300	-3.275500	-1.072700
C	2.044200	-3.276800	0.000000
C	4.185000	-3.265400	-0.677800
C	4.185000	-3.265400	0.677800
C	2.451800	-3.180100	2.460200
C	2.451800	-3.180100	-2.460200
N	-2.868300	-3.275500	-1.072700
N	-2.868300	-3.275500	1.072700
C	-2.044200	-3.276800	0.000000
C	-4.185000	-3.265400	0.677800
C	-4.185000	-3.265400	-0.677800
C	-2.451800	-3.180100	-2.460200
C	-2.451800	-3.180100	2.460200
H	4.998300	-3.259900	-1.387100
H	4.998300	-3.259900	1.387100
H	1.363700	-3.243000	2.497600
H	2.879100	-4.007000	3.034200
H	2.781400	-2.220800	2.873200
H	1.363700	-3.243000	-2.497600
H	2.781400	-2.220800	-2.873200
H	2.879100	-4.007000	-3.034200
H	-4.998300	-3.259900	1.387100
H	-4.998300	-3.259900	-1.387100
H	-1.363700	-3.243000	-2.497600
H	-2.879100	-4.007000	-3.034200
H	-2.781400	-2.220800	-2.873200
H	-1.363700	-3.243000	2.497600
H	-2.781400	-2.220800	2.873200
H	-2.879100	-4.007000	3.034200
O	-3.851600	0.000000	2.837700
C	-5.424300	0.000000	4.621800
C	-5.237100	0.000000	3.120700
H	-3.733100	0.000000	1.869200
H	-6.487900	0.000000	4.881100
H	-4.959200	-0.885600	5.067000
H	-4.959200	0.885600	5.067000
H	-5.727500	-0.884200	2.681200
H	-5.727500	0.884200	2.681200

C	-5.424300	0.000000	-4.621800
O	-3.851600	0.000000	-2.837700
C	-5.237100	0.000000	-3.120700
H	-4.959200	-0.885600	-5.067000
H	-6.487900	0.000000	-4.881100
H	-4.959200	0.885600	-5.067000
H	-3.733100	0.000000	-1.869200
H	-5.727500	0.884200	-2.681200
H	-5.727500	-0.884200	-2.681200
C	5.237100	0.000000	-3.120700
O	3.851600	0.000000	-2.837700
C	5.424300	0.000000	-4.621800
H	5.727500	-0.884200	-2.681200
H	5.727500	0.884200	-2.681200
H	3.733100	0.000000	-1.869200
H	4.959200	0.885600	-5.067000
H	6.487900	0.000000	-4.881100
H	4.959200	-0.885600	-5.067000
O	3.851600	0.000000	2.837700
C	5.237100	0.000000	3.120700
C	5.424300	0.000000	4.621800
H	3.733100	0.000000	1.869200
H	5.727500	0.884200	2.681200
H	5.727500	-0.884200	2.681200
H	6.487900	0.000000	4.881100
H	4.959200	0.885600	5.067000
H	4.959200	-0.885600	5.067000

17. The S_0 .xyz files of II and its complexes

II

Au 0.003100 -0.207200 0.009400
N 0.026700 -0.287900 3.056500
N 2.041400 -0.095600 2.274100
C 0.742300 -0.174200 1.912300
C 0.867900 -0.284700 4.156900
C 0.618300 -0.365000 5.524200
H -0.389600 -0.444100 5.919100
C 1.724500 -0.333400 6.364200
H 1.576600 -0.390600 7.438200
C 3.029500 -0.227100 5.857200
H 3.866100 -0.207800 6.548800
C 3.276500 -0.144600 4.492700
H 4.287000 -0.067600 4.105000
C 2.167200 -0.170700 3.651800
C 3.165300 -0.023200 1.360900
H 2.796800 0.308600 0.389200
H 3.628400 -1.008500 1.251800
H 3.888000 0.706600 1.735200
C -1.412800 -0.405300 3.140000
H -1.814800 -0.497500 2.130400
H -1.840300 0.476400 3.627600
H -1.679600 -1.301400 3.707900
N -0.020300 -0.351400 -3.035300
N -2.035600 -0.149600 -2.256900
C -0.736200 -0.216300 -1.893600
C -0.861500 -0.374000 -4.135500
C -0.611600 -0.481900 -5.500900
H 0.396600 -0.565700 -5.894100
C -1.717800 -0.471600 -6.341200
H -1.569800 -0.550600 -7.413800
C -3.023300 -0.359100 -5.836400
H -3.859900 -0.357000 -6.528200
C -3.270500 -0.249000 -4.473900
H -4.281300 -0.167400 -4.087800
C -2.161100 -0.253800 -3.632700
C -3.159700 -0.062000 -1.345300
H -2.792300 0.291200 -0.380800
H -3.884800 0.657300 -1.734800
H -3.619500 -1.046400 -1.215700
C 1.419600 -0.465700 -3.116400
H 1.821900 -0.535200 -2.105100
H 1.689500 -1.372500 -3.665400
H 1.844100 0.407000 -3.622500
N -3.128300 -3.370400 -0.376800
C -1.971600 -3.365400 -0.209100
N 3.145000 -3.351200 0.462100
C 1.988300 -3.353500 0.294300
Au 0.008300 -3.358600 0.042600
Au 0.013600 -6.507500 0.075500
N -0.000600 -6.359800 3.120000
N 2.024000 -6.564600 2.366700
C 0.729100 -6.497300 1.987500
C 0.827100 -6.336500 4.230400
C 0.560500 -6.226500 5.592500
H -0.452400 -6.141400 5.973100
C 1.656300 -6.236500 6.446400
H 1.495100 -6.156000 7.516900
C 2.967800 -6.350800 5.957800
H 3.795800 -6.352600 6.659900
C 3.231700 -6.463000 4.598600
H 4.247000 -6.546100 4.225200
C 2.132700 -6.458500 3.743800
C 3.158800 -6.654500 1.468600
H 2.803400 -7.013500 0.501800
H 3.880600 -7.370500 1.870200
H 3.618500 -5.670100 1.339200
C -1.441400 -6.243900 3.183100
H -1.830900 -6.172300 2.167000
H -1.716900 -5.337300 3.729800
H -1.873300 -7.116700 3.682700
N 0.027500 -6.424200 -2.971600
N -1.996500 -6.619600 -2.214000
C -0.701800 -6.540100 -1.836300
C -0.800300 -6.427100 -4.082100
C -0.534000 -6.345000 -5.446200
H 0.478600 -6.264700 -5.828700
C -1.629800 -6.376700 -6.299600
H -1.468900 -6.318300 -7.371700
C -2.940900 -6.484900 -5.808700
H -3.768900 -6.504300 -6.510500
C -3.204400 -6.569200 -4.447400
H -4.219500 -6.647700 -4.072200
C -2.105500 -6.543000 -3.593000
C -3.131000 -6.694200 -1.314100
H -2.774400 -7.031600 -0.339900
H -3.593900 -5.708800 -1.205300
H -3.850500 -7.420800 -1.700400
C 1.467800 -6.304900 -3.037200
H 1.857100 -6.210600 -2.022800
H 1.902600 -7.186700 -3.518200
H 1.740500 -5.409300 -3.602900

II-S1

97
Molecule Name
Au -0.304500 3.395200 -0.068800
N 2.615700 3.682500 0.785600
N 2.426800 3.733300 -1.373600
C 1.722100 3.606800 -0.223000
C 3.777100 3.881000 -1.102500
C 2.315300 3.569500 2.197400
H 1.245000 3.391700 2.308800
H 2.857700 2.717100 2.613200
C 1.872100 3.721900 -2.708500
H 0.788000 3.626200 -2.633000
H 2.115100 4.655400 -3.225300
H 2.591500 4.495000 2.712100
H 2.273100 2.877100 -3.277100
N -3.201200 2.919300 -0.910100
N -3.099600 3.777000 1.079200
C -2.343400 3.333500 0.046500
C -4.446700 3.647500 0.785100
C -2.839000 2.333500 -2.185300
H -1.773700 2.100100 -2.167300
H -3.409400 1.411000 -2.334500
C -2.597800 4.298700 2.331100
H -1.508800 4.333600 2.281100
H -2.982800 5.309300 2.497900
H -3.048500 3.038700 -2.996500
H -2.901800 3.650600 3.158600
C 3.898200 3.841600 0.289200

C	5.134200	3.959100	0.918000
C	4.884300	4.046300	-1.930100
C	6.240400	4.123900	0.094800
H	5.232400	3.916000	1.997600
C	6.118000	4.168800	-1.303500
H	4.793200	4.080400	-3.011100
H	7.224600	4.219200	0.542800
H	7.009200	4.301400	-1.909000
C	-4.510600	3.096700	-0.497700
C	-5.727300	2.842600	-1.124400
C	-5.596200	3.961400	1.505300
C	-6.875600	3.158600	-0.409000
H	-5.775800	2.413100	-2.119400
C	-6.811400	3.706900	0.882500
H	-5.550600	4.386100	2.503100
H	-7.847000	2.979500	-0.859700
H	-7.733800	3.938600	1.405900
N	-2.920900	-0.352000	0.110300
C	-1.793500	-0.214500	0.377700
N	3.178800	0.416300	1.559900
C	2.061200	0.270700	1.251200
Au	0.138300	0.025900	0.795900
Au	0.640400	-3.362000	-0.060800
N	3.507000	-2.890400	0.889300
N	3.411900	-3.019900	-1.272900
C	2.658900	-3.058600	-0.146900
C	4.745400	-2.816000	-0.958400
C	3.137800	-2.834200	2.288100
H	2.054200	-2.933300	2.362800
H	3.621900	-3.649800	2.834200
C	2.918100	-3.169300	-2.623400
H	1.844100	-3.356300	-2.581500
H	3.106400	-2.257000	-3.197800
H	3.431000	-1.865000	2.698200
H	3.411200	-4.014900	-3.112800
N	-2.267300	-3.547400	-0.973900
N	-1.994800	-4.501800	0.954400
C	-1.352200	-3.810300	-0.017100
C	-3.326400	-4.683800	0.620200
C	-2.028600	-2.810400	-2.199100
H	-1.083500	-2.274800	-2.101400
H	-1.979600	-3.498300	-3.049800
C	-1.403200	-4.978800	2.184900
H	-0.335700	-4.755400	2.166700
H	-1.863900	-4.480900	3.043500
H	-2.837600	-2.089200	-2.348800
H	-1.542000	-6.060600	2.272700
C	4.804400	-2.726400	0.435000
C	6.007500	-2.516600	1.102700
C	7.147000	-2.407600	0.316400
H	6.054200	-2.433600	2.183300
C	5.886100	-2.707500	-1.748900
C	7.088200	-2.503300	-1.083500
H	8.107200	-2.242900	0.795300
H	5.843800	-2.779800	-2.831100
H	8.004400	-2.414500	-1.659100
C	-3.500500	-4.070000	-0.623200
C	-4.731200	-4.072500	-1.273600
C	-5.778300	-4.713600	-0.623600
H	-4.866700	-3.586800	-2.233800
C	-4.375700	-5.322500	1.275600
C	-5.604500	-5.326700	0.628200
H	-6.755900	-4.742500	-1.095100
H	-4.244800	-5.796600	2.243100
H	-6.450700	-5.816000	1.100500
O	-4.652200	-0.581700	-2.075000
H	-4.182800	-0.526200	-1.214600
C	-6.029700	-0.736200	-1.820300
H	-6.445100	0.102700	-1.243800
H	-6.542900	-0.774600	-2.786600
H	-6.253100	-1.665000	-1.275800

II-2S1

103

Molecule Name

Au	-0.220000	-3.436200	-0.001800
N	2.733300	-3.393100	-0.798300
N	2.458800	-4.267100	1.167700
C	1.804200	-3.677000	0.138800
C	3.811700	-4.364700	0.889500
C	2.493200	-2.721700	-2.060400
H	1.486800	-2.300900	-2.043400
H	3.223100	-1.914600	-2.179400
C	1.857200	-4.726400	2.400300
H	0.778500	-4.576300	2.337700
H	2.064400	-5.791000	2.544900
H	2.582500	-3.432400	-2.888700
H	2.254600	-4.159000	3.247400
N	-3.148100	-3.062300	0.801600
N	-2.977400	-3.930200	-1.178800
C	-2.258400	-3.436100	-0.142300
C	-4.332300	-3.876100	-0.898800
C	-2.832300	-2.438200	2.071300
H	-1.780300	-2.149300	2.064000
H	-3.454600	-1.545900	2.194300
C	-2.433900	-4.435600	-2.420200
H	-1.345100	-4.403300	-2.361900
H	-2.755300	-5.469700	-2.577500
H	-3.016600	-3.139300	2.892100
H	-2.771000	-3.817200	-3.257700
C	3.987300	-3.802800	-0.377800
C	5.237800	-3.750600	-0.986700
C	4.878600	-4.892700	1.611600
C	6.303100	-4.282100	-0.270500
H	5.373800	-3.308100	-1.967500
C	6.127400	-4.841600	1.005800
H	4.745800	-5.326400	2.597600
H	7.296500	-4.266100	-0.708400
H	6.988100	-5.244800	1.530500
C	-4.441100	-3.318600	0.378000
C	-5.676800	-3.134300	0.991600
C	-5.453500	-4.266200	-1.626300
C	-6.796700	-3.527900	0.269800
H	-5.760600	-2.697200	1.980700
C	-6.687600	-4.082300	-1.016100
H	-5.372300	-4.696400	-2.619400
H	-7.781300	-3.405900	0.711000
H	-7.589300	-4.375500	-1.544700
N	-3.116400	0.169700	-0.484700
C	-1.958600	0.107400	-0.346400
N	3.116200	-0.169400	0.483500
C	1.958500	-0.107100	0.345100
Au	-0.000100	0.000200	-0.000700
Au	0.220300	3.436300	0.002400
N	3.148200	3.063500	-0.802000
N	2.978000	3.929500	1.179300
C	2.258800	3.436400	0.142500

C	4.332800	3.875800	0.898900
C	2.832200	2.440300	-2.072100
H	1.780600	2.149600	-2.064000
H	3.014400	3.142500	-2.892300
C	2.434800	4.433600	2.421400
H	1.346000	4.401500	2.363100
H	2.772000	3.814200	3.258000
H	3.455700	1.549100	-2.196600
H	2.756300	5.467500	2.579700
N	-2.733000	3.391900	0.799000
N	-2.458700	4.267600	-1.166300
C	-1.803900	3.676800	-0.137900
C	-3.811600	4.364600	-0.888100
C	-2.492600	2.719800	2.060700
H	-1.485500	2.300500	2.043900
H	-2.583400	3.429600	2.889500
C	-1.857300	4.728300	-2.398400
H	-0.778500	4.578400	-2.336000
H	-2.254500	4.161800	-3.246100
H	-3.221300	1.911600	2.178600
H	-2.064600	5.793100	-2.541800
C	4.441300	3.319600	-0.378500
C	5.676800	3.136000	-0.992600
C	6.797000	3.528800	-0.270800
H	5.760500	2.699900	-1.982200
C	5.454200	4.265300	1.626400
C	6.688200	4.081900	1.015700
H	7.781400	3.407200	-0.712400
H	5.373300	4.694500	2.619900
H	7.590000	4.374600	1.544400
C	-3.987000	3.801400	0.378800
C	-5.237600	3.748100	0.987500
C	-6.303000	4.279900	0.271700
H	-5.373500	3.304400	1.967800
C	-4.878700	4.892800	-1.609700
C	-6.127500	4.840700	-1.004000
H	-7.296500	4.263000	0.709600
H	-4.746000	5.327400	-2.595300
H	-6.988300	5.244100	-1.528400
O	-4.901700	0.287200	1.707100
H	-4.439300	0.256900	0.843800
C	-6.289100	0.361400	1.466200
H	-6.662400	-0.508300	0.907700
H	-6.791200	0.385500	2.438700
H	-6.568600	1.268600	0.912200
O	4.898300	-0.285900	-1.709900
H	4.436200	-0.255700	-0.846300
C	6.285700	-0.361700	-1.469600
H	6.787300	-0.387200	-2.442300
H	6.660400	0.508000	-0.912000
H	6.564300	-1.268700	-0.915000

II-3S1

109

Molecule Name

Au	0.005900	0.089300	3.329300
N	-0.975600	-2.799300	3.297900
N	1.192700	-2.715700	3.344600
C	0.078300	-1.952000	3.284900
C	0.856700	-4.057000	3.397300
C	-2.366900	-2.427200	3.137600
H	-2.427100	-1.340400	3.063400
H	-2.759000	-2.875600	2.217700
C	2.555700	-2.234100	3.239700
H	2.543200	-1.145500	3.312400
H	3.156300	-2.639600	4.059200
H	-2.948800	-2.765900	4.000500
H	2.974600	-2.537100	2.273300
N	0.865600	3.021000	3.186600
N	-1.006500	2.801800	4.259200
C	-0.053400	2.115600	3.583500
C	-0.698800	4.151400	4.295500
C	2.060500	2.739400	2.416100
H	1.977500	1.733200	2.002700
H	2.136500	3.464300	1.599800
C	-2.196800	2.234200	4.852900
H	-2.160800	1.150400	4.734800
H	-2.236200	2.477800	5.918900
H	2.946200	2.803000	3.056900
H	-3.089800	2.625100	4.355800
C	-0.539900	-4.110500	3.368600
C	-1.226400	-5.320400	3.431300
C	1.630100	-5.211600	3.488100
C	-0.456000	-6.473500	3.519200
H	-2.310700	-5.362700	3.418800
C	0.947000	-6.420100	3.547200
H	2.714300	-5.172200	3.519100
H	-0.950300	-7.438500	3.575500
H	1.510600	-7.344900	3.623800
C	0.507800	4.291700	3.604700
C	1.124400	5.530700	3.454700
C	-1.349500	5.243700	4.863400
C	0.479300	6.621000	4.024600
H	2.055100	5.639500	2.908800
C	-0.735400	6.480600	4.715800
H	-2.288700	5.138800	5.396900
H	0.925300	7.606800	3.934700
H	-1.205400	7.360200	5.144800
N	-0.625600	3.343300	0.000000
C	-0.597600	2.176700	0.000000
N	0.014300	-2.937800	0.000000
C	-0.149700	-1.779400	0.000000
Au	-0.404100	0.194700	0.000000
Au	0.005900	0.089300	-3.329300
N	-0.975600	-2.799300	-3.297900
N	1.192700	-2.715700	-3.344600
C	0.078300	-1.952000	-3.284900
C	0.856700	-4.057000	-3.397300
C	-2.366900	-2.427200	-3.137600
H	-2.427100	-1.340400	-3.063400
H	-2.948800	-2.765900	-4.000500
C	2.555700	-2.234100	-3.239700
H	2.543200	-1.145500	-3.312400
H	2.974600	-2.537100	-2.273300
H	-2.759000	-2.875600	-2.217700
H	3.156300	-2.639600	-4.059200
N	0.865600	3.021000	-3.186600
N	-1.006500	2.801800	-4.259200
C	-0.053400	2.115600	-3.583500
C	-0.698800	4.151400	-4.295500
C	2.060500	2.739400	-2.416100
H	1.977500	1.733200	-2.002700
H	2.946200	2.803000	-3.056900
C	-2.196800	2.234200	-4.852900
H	-2.160800	1.150400	-4.734800
H	-3.089800	2.625100	-4.355800
H	2.136500	3.464300	-1.599800

H -2.236200 2.477800 -5.918900
C -0.539900 -4.110500 -3.368600
C -1.226400 -5.320400 -3.431300
C -0.456000 -6.473500 -3.519200
H -2.310700 -5.362700 -3.418800
C 1.630100 -5.211600 -3.488100
C 0.947000 -6.420100 -3.547200
H -0.950300 -7.438500 -3.575500
H 2.714300 -5.172200 -3.519100
H 1.510600 -7.344900 -3.623800
C 0.507800 4.291700 -3.604700
C 1.124400 5.530700 -3.454700
C 0.479300 6.621000 -4.024600
H 2.055100 5.639500 -2.908800
C -1.349500 5.243700 -4.863400
C -0.735400 6.480600 -4.715800
H 0.925300 7.606800 -3.934700
H -2.288700 5.138800 -5.396900
H -1.205400 7.360200 -5.144800
O 1.591000 5.095000 0.000000
H 0.723500 4.638300 0.000000
C 1.357200 6.486000 0.000000
H 0.802900 6.814200 0.890600
H 2.331800 6.984400 0.000000
H 0.802900 6.814200 -0.890600
O -2.727000 -3.984500 0.000000
H -1.769800 -3.799900 0.000000
C -2.912100 -5.382700 0.000000
H -3.989800 -5.569200 0.000000
H -2.478100 -5.862200 -0.889100
H -2.478100 -5.862200 0.889100
H 1.916600 -3.416200 0.000000
O 2.890800 -3.473800 0.000000
C 3.249900 -4.837900 0.000000
H 4.342500 -4.888600 0.000000
H 2.879200 -5.368000 0.889100
H 2.879200 -5.368000 -0.889100

II-4S1

115
Molecule Name
Au 0.000000 3.219900 0.000000
N 2.850200 3.334300 1.085000
N 2.850200 3.334300 -1.085000
C 2.043800 3.286700 0.000000
C 4.176700 3.409800 -0.698800
C 2.428500 3.200500 2.464700
H 1.338300 3.206800 2.494400
H 2.802100 2.253500 2.871100
C 2.428500 3.200500 -2.464700
H 1.338300 3.206800 -2.494400
H 2.807700 4.041300 -3.053200
H 2.807700 4.041300 3.053200
H 2.802100 2.253500 -2.871100
N -2.850200 3.334300 -1.085000
N -2.850200 3.334300 1.085000
C -2.043800 3.286700 0.000000
C -4.176700 3.409800 0.698800
C -2.428500 3.200500 -2.464700
H -1.338300 3.206800 -2.494400
H -2.802100 2.253500 -2.871100
C -2.428500 3.200500 2.464700
H -1.338300 3.206800 2.494400
H -2.807700 4.041300 3.053200
H -2.807700 4.041300 -3.053200
H -2.802100 2.253500 2.871100
C 4.176700 3.409800 0.698800
C 5.359100 3.492700 1.429700
C 5.359100 3.492700 -1.429700
C 6.540100 3.572800 0.702200
H 5.360700 3.499900 2.514900
C 6.540100 3.572800 -0.702200
H 5.360700 3.499900 -2.514900
H 7.485400 3.643500 1.231500
H 7.485400 3.643500 -1.231500
C -4.176700 3.409800 -0.698800
C -5.359100 3.492700 -1.429700
C -5.359100 3.492700 1.429700
C -6.540100 3.572800 -0.702200
H -5.360700 3.499900 -2.514900
C -6.540100 3.572800 0.702200
H -5.360700 3.499900 2.514900
H -7.485400 3.643500 -1.231500
H -7.485400 3.643500 1.231500
N -3.160000 0.000000 0.000000
C -1.990300 0.000000 0.000000
N 3.160000 0.000000 0.000000
C 1.990300 0.000000 0.000000
Au 0.000000 0.000000 0.000000
Au 0.000000 -3.219900 0.000000
N 2.850200 -3.334300 1.085000
N 2.850200 -3.334300 -1.085000
C 2.043800 -3.286700 0.000000
C 4.176700 -3.409800 -0.698800
C 2.428500 -3.200500 2.464700
H 1.338300 -3.206800 2.494400
H 2.807700 -4.041300 3.053200
C 2.428500 -3.200500 -2.464700
H 1.338300 -3.206800 -2.494400
H 2.802100 -2.253500 -2.871100
H 2.802100 -2.253500 2.871100
H 2.807700 -4.041300 -3.053200
N -2.850200 -3.334300 -1.085000
N -2.850200 -3.334300 1.085000
C -2.043800 -3.286700 0.000000
C -4.176700 -3.409800 0.698800
C -2.428500 -3.200500 -2.464700
H -1.338300 -3.206800 -2.494400
H -2.807700 -4.041300 -3.053200
C -2.428500 -3.200500 2.464700
H -1.338300 -3.206800 2.494400
H -2.802100 -2.253500 2.871100
H -2.802100 -2.253500 -2.871100
H -2.807700 -4.041300 3.053200
C 4.176700 -3.409800 0.698800
C 5.359100 -3.492700 1.429700
C 6.540100 -3.572800 0.702200
H 5.360700 -3.499900 2.514900
C 5.359100 -3.492700 -1.429700
C 6.540100 -3.572800 -0.702200
H 7.485400 -3.643500 1.231500
H 5.360700 -3.499900 -2.514900
H 7.485400 -3.643500 -1.231500
C -4.176700 -3.409800 -0.698800
C -5.359100 -3.492700 -1.429700
C -6.540100 -3.572800 -0.702200
H -5.360700 -3.499900 -2.514900

C -5.359100 -3.492700 1.429700
C -6.540100 -3.572800 0.702200
H -7.485400 -3.643500 -1.231500
H -5.360700 -3.499900 2.514900
H -7.485400 -3.643500 1.231500
O -3.834500 0.000000 -2.858700
H -3.744700 0.000000 -1.888000
C -5.209900 0.000000 -3.171800
H -5.726800 0.889300 -2.783100
H -5.297300 0.000000 -4.262000
H -5.726800 -0.889300 -2.783100
O 3.834500 0.000000 2.858700
H 3.744700 0.000000 1.888000
C 5.209900 0.000000 3.171800
H 5.297300 0.000000 4.262000
H 5.726800 -0.889300 2.783100
H 5.726800 0.889300 2.783100
H 3.744700 0.000000 -1.888000
O 3.834500 0.000000 -2.858700
C 5.209900 0.000000 -3.171800
H 5.297300 0.000000 -4.262000
H 5.726800 0.889300 -2.783100
H 5.726800 -0.889300 -2.783100
H -3.744700 0.000000 1.888000
O -3.834500 0.000000 2.858700
C -5.209900 0.000000 3.171800
H -5.726800 -0.889300 2.783100
H -5.297300 0.000000 4.262000
H -5.726800 0.889300 2.783100

II-S2

100
Molecule Name
Au -0.620000 3.386100 0.019800
N -3.532800 3.002600 -0.824300
N -3.356500 3.146500 1.332000
C -2.643400 3.152200 0.179600
C -4.706000 2.981300 1.066900
C -3.216300 2.924800 -2.234900
H -2.134700 3.004300 -2.350600
H -3.541900 1.956900 -2.623500
C -2.810100 3.291400 2.662600
H -1.735200 3.458700 2.580000
H -3.269200 4.147900 3.165800
H -3.705800 3.743400 -2.771600
H -2.993500 2.385000 3.247900
N 2.320500 3.478000 0.834600
N 2.016100 4.434700 -1.087900
C 1.383700 3.766700 -0.093500
C 3.362700 4.576200 -0.797100
C 2.097800 2.752700 2.069600
H 1.134200 2.245900 2.003300
H 2.888800 2.007500 2.197100
C 1.400300 4.926000 -2.300800
H 0.328100 4.732300 -2.249300
H 1.566400 6.003400 -2.395600
H 2.096100 3.444600 2.918500
H 1.820500 4.413800 -3.171700
C -4.817500 2.882400 -0.322700
C -6.049500 2.704900 -0.945300
C -5.820700 2.914500 1.898200
C -7.163000 2.637500 -0.118300
H -6.137200 2.614500 -2.022800
C -7.051400 2.742500 1.277600
H -5.737800 2.993600 2.977500
H -8.144300 2.498600 -0.561500
H -7.948700 2.686600 1.886200
C 3.557500 3.960600 0.442400
C 4.808100 3.927000 1.052800
C 4.409600 5.180500 -1.488100
C 5.853000 4.534100 0.367500
H 4.959600 3.440400 2.010300
C 5.658000 5.148900 -0.880200
H 4.262200 5.656000 -2.452500
H 6.845700 4.533700 0.807300
H 6.503200 5.610600 -1.381200
N 2.814600 0.245600 -0.252300
C 1.673700 0.143000 -0.474400
N -3.355700 -0.322400 -1.477100
C -2.224000 -0.216000 -1.206000
Au -0.279100 -0.035900 -0.819300
Au 0.111900 -3.422000 0.034200
N -2.834800 -3.604200 -0.754500
N -2.599600 -3.670300 1.399800
C -1.916900 -3.563600 0.234000
C -3.959900 -3.769900 1.158700
C -2.562400 -3.495200 -2.172200
H -1.490200 -3.347500 -2.306900
H -2.876600 -4.410500 -2.683400
C -2.014800 -3.683500 2.721800
H -0.930200 -3.622300 2.622200
H -2.375500 -2.829200 3.302900
H -3.089700 -2.626600 -2.573600
H -2.276200 -4.611400 3.239900
N 3.043700 -3.023400 0.794200
N 2.863000 -3.927000 -1.169000
C 2.148500 -3.431700 -0.130000
C 4.220700 -3.836500 -0.911400
C 2.732900 -2.396600 2.063600
H 1.679500 -2.113800 2.059800
H 2.925000 -3.094800 2.885100
C 2.312300 -4.463100 -2.394100
H 1.224600 -4.465400 -2.314000
H 2.612000 -3.844000 -3.245200
H 3.347500 -1.498500 2.183100
H 2.663500 -5.488200 -2.546200
C -4.110600 -3.720500 -0.229800
C -5.363600 -3.792000 -0.831400
C -6.456300 -3.921400 0.015700
H -5.484000 -3.740500 -1.908300
C -5.053600 -3.900000 2.010200
C -6.304400 -3.976500 1.410700
H -7.453000 -3.980000 -0.410400
H -4.940000 -3.941900 3.088800
H -7.186000 -4.080500 2.035600
C 4.335700 -3.256400 0.354800
C 5.576200 -3.029500 0.944400
C 6.694200 -3.403000 0.209300
H 5.665200 -2.577800 1.926800
C 5.339800 -4.207900 -1.651700
C 6.578600 -3.980400 -1.065800
H 7.682300 -3.246200 0.631100
H 5.254000 -4.655300 -2.636700
H 7.479000 -4.256600 -1.605700
O 4.639300 0.444900 1.861300
H 4.137700 0.396500 1.018400
C 6.017800 0.548700 1.562100

H	6.224500	1.463200	0.984800
H	6.347700	-0.299900	0.943400
C	6.793600	0.572900	2.861900
H	6.611900	-0.340700	3.438900
H	7.869200	0.654900	2.673500
H	6.485900	1.422500	3.481600

II-2S2

109

Molecule Name

N	3.087400	0.002200	0.652800
C	1.937100	0.001400	0.452600
Au	-0.002500	-0.000000	-0.000100
C	-1.942200	-0.001400	-0.452500
N	-3.092700	-0.002300	-0.652100
Au	0.003600	-3.448500	-0.000200
N	-2.976200	-3.235300	0.659000
N	-2.661900	-4.123800	-1.294400
C	-2.022700	-3.571500	-0.235300
C	-4.029400	-4.144500	-1.078800
C	-2.756200	-2.577600	1.932000
C	-2.031600	-4.616400	-2.499500
N	2.982900	-3.230900	-0.659800
N	2.670200	-4.120500	1.293400
C	2.030000	-3.568700	0.234600
C	4.037600	-4.139600	1.077400
C	2.761900	-2.572800	-1.932400
C	2.040800	-4.614200	2.498500
C	-4.230800	-3.573600	0.180800
C	-5.503000	-3.451900	0.732000
C	-5.090200	-4.611300	-1.850300
C	-6.562700	-3.922400	-0.033400
C	-6.360600	-4.490400	-1.302000
C	4.238000	-3.568000	-0.182000
C	5.509900	-3.444800	-0.733700
C	5.099200	-4.605500	1.848400
C	6.570300	-3.914500	0.031200
C	6.369200	-4.483100	1.299700
H	-1.728000	-2.213800	1.961200
H	-3.444500	-1.730900	2.019300
H	-2.922000	-3.281800	2.754100
H	-0.950200	-4.526800	-2.387800
H	-2.291200	-5.667800	-2.655800
H	-2.357800	-4.027800	-3.362500
H	1.732900	-2.211400	-1.961800
H	3.448300	-1.724500	-2.018700
H	2.929800	-3.276000	-2.754900
H	0.959200	-4.526500	2.386800
H	2.302100	-5.665200	2.654700
H	2.365900	-4.025200	3.361500
H	-5.658800	-3.003900	1.707600
H	-4.937000	-5.051600	-2.830400
H	-7.572500	-3.850300	0.358900
H	-7.217600	-4.844300	-1.866600
H	5.665000	-2.996300	-1.709100
H	4.946800	-5.046300	2.828400
H	7.580000	-3.841300	-0.361400
H	7.226900	-4.836200	1.863900
Au	-0.002500	3.448600	-0.000300
N	-2.982100	3.231000	0.658500
N	-2.668800	4.120000	-1.294900
C	-2.028900	3.568700	-0.235700
C	-4.036400	4.138700	-1.079400
C	-2.761200	2.573700	1.931500
C	-2.039100	4.613500	-2.499900
N	2.977300	3.235100	-0.659300
N	2.662900	4.124500	1.293800
C	2.023700	3.571700	0.234900
C	4.030400	4.145400	1.078000
C	2.757400	2.576700	-1.931900
C	2.032600	4.617600	2.498600
C	-4.237100	3.567500	0.180100
C	-5.509200	3.443900	0.731200
C	-6.569500	3.912800	-0.034400
C	-5.097800	4.603900	-1.851100
C	-6.368000	4.481000	-1.302900
C	4.231800	3.574000	-0.181300
C	5.504000	3.452300	-0.732600
C	6.563600	3.923400	0.032500
C	5.091200	4.612800	1.849200
C	6.361500	4.491900	1.300800
H	-1.732500	2.211600	1.961000
H	-2.928500	3.277500	2.753600
H	-3.448100	1.725700	2.018600
H	-0.957500	4.525900	-2.387900
H	-2.364000	4.024400	-3.362800
H	-2.300500	5.664500	-2.656400
H	1.729000	2.213700	-1.961400
H	2.924300	3.280200	-2.754400
H	3.445100	1.729400	-2.018200
H	0.951100	4.528200	2.386800
H	2.358400	4.029200	3.361800
H	2.292400	5.669000	2.654700
H	-5.664500	2.995800	1.706800
H	-7.579300	3.839100	0.357800
H	-4.945100	5.044400	-2.831200
H	-7.225600	4.833600	-1.867700
H	5.659800	3.003800	-1.707900
H	7.573500	3.851500	-0.359800
H	4.937900	5.053500	2.829200
H	7.218500	4.846200	1.865200
O	4.983200	0.003700	-1.452300
C	6.366400	0.004700	-1.152500
C	7.141000	0.005000	-2.453000
H	4.480500	0.003300	-0.610900
H	6.635500	-0.879400	-0.555100
H	6.634200	0.889500	-0.555400
H	8.219600	0.005900	-2.264500
H	6.895800	-0.879300	-3.051800
H	6.894500	0.888700	-3.052200
O	-4.983200	-0.004300	1.457700
C	-6.367400	-0.004800	1.162800
C	-7.137400	-0.004800	2.466000
H	-4.483200	-0.003800	0.614600
H	-6.638300	0.879400	0.566300
H	-6.637700	-0.889400	0.566600
H	-8.216700	-0.005300	2.281300
H	-6.889100	-0.888600	3.064300
H	-6.889800	0.879400	3.064000

II-3S2

118

Molecule Name

Au	-0.132400	-3.324300	-0.041700
N	2.781800	-3.295200	-0.944100

N	2.639700	-3.344100	1.221000
C	1.906400	-3.282700	0.086600
C	3.989600	-3.397300	0.921400
C	2.447100	-3.132700	-2.344600
H	1.362700	-3.053000	-2.433200
H	2.909900	-2.214500	-2.723900
C	2.121600	-3.239900	2.570500
H	1.033400	-3.308200	2.528300
H	2.507300	-4.062100	3.180500
H	2.796900	-3.996900	-2.917900
H	2.416900	-2.275500	2.999200
N	-3.085000	-3.189300	0.744000
N	-2.818900	-4.238400	-1.135300
C	-2.156800	-3.575300	-0.156800
C	-4.175900	-4.277600	-0.862400
C	-2.833000	-2.432300	1.954100
H	-1.824600	-2.019300	1.900800
H	-3.558500	-1.615700	2.021100
C	-2.221800	-4.816900	-2.318700
H	-1.139000	-4.703300	-2.252300
H	-2.467600	-5.881300	-2.379700
H	-2.918900	-3.082900	2.830800
H	-2.587300	-4.305600	-3.214500
C	4.080700	-3.367200	-0.473300
C	5.308600	-3.430300	-1.126800
C	5.122800	-3.489500	1.725500
C	6.440400	-3.520300	-0.325800
H	5.380400	-3.416400	-2.209600
C	6.349200	-3.549300	1.075100
H	5.054500	-3.520600	2.808200
H	7.418400	-3.576600	-0.794000
H	7.258300	-3.626800	1.663500
C	-4.346300	-3.602300	0.349200
C	-5.600500	-3.461200	0.936400
C	-5.251300	-4.836800	-1.547500
C	-6.674200	-4.022600	0.257100
H	-5.732700	-2.929300	1.872400
C	-6.503300	-4.697400	-0.962900
H	-5.122700	-5.358200	-2.490700
H	-7.670900	-3.938600	0.679600
H	-7.370700	-5.120200	-1.460500
N	-3.364700	0.000100	-0.741900
C	-2.199200	0.000000	-0.683400
N	2.898300	0.000000	0.056100
C	1.744300	0.000000	-0.135800
Au	-0.222900	0.000000	-0.439200
Au	-0.131800	3.324300	-0.041900
N	2.782400	3.294900	-0.944100
N	2.640300	3.343900	1.221000
C	1.907000	3.282600	0.086500
C	3.990100	3.396800	0.921400
C	2.447800	3.132300	-2.344600
H	1.363300	3.052900	-2.433300
H	2.797900	3.996300	-2.918100
C	2.122000	3.239900	2.570400
H	1.033900	3.308200	2.528100
H	2.417400	2.275500	2.999300
H	2.910400	2.213900	-2.723800
H	2.507700	4.062100	3.180400
N	-3.084300	3.189700	0.743900
N	-2.818200	4.238600	-1.135600
C	-2.156100	3.575500	-0.157000
C	-4.175200	4.278000	-0.862500
C	-2.832400	2.432900	1.954100
H	-1.824000	2.019900	1.901000
H	-2.918500	3.083600	2.830700
C	-2.221100	4.816800	-2.319000
H	-1.138300	4.703100	-2.252700
H	-2.586800	4.305500	-3.214700
H	-3.557900	1.616200	2.021100
H	-2.466800	5.881300	-2.380200
C	4.081300	3.366800	-0.473200
C	5.309200	3.429600	-1.126700
C	6.441100	3.519400	-0.325700
H	5.381100	3.415700	-2.209500
C	5.123300	3.488900	1.725600
C	6.349800	3.548400	1.075200
H	7.419100	3.575500	-0.793800
H	5.055000	3.520000	2.808300
H	7.258900	3.625800	1.663700
C	-4.345700	3.602900	0.349100
C	-5.599800	3.461900	0.936400
C	-6.673600	4.023400	0.257000
H	-5.732100	2.930200	1.872400
C	-5.250600	4.837200	-1.547700
C	-6.502600	4.698000	-0.963000
H	-7.670200	3.939500	0.679600
H	-5.122000	5.358400	-2.491000
H	-7.369900	5.120800	-1.460600
O	-5.158800	0.000500	1.446000
H	-4.687900	0.000500	0.585800
C	-6.551800	0.000600	1.193900
H	-6.839200	-0.884500	0.606100
H	-6.839100	0.885700	0.606100
C	-7.283300	0.000500	2.518900
H	-7.019200	0.884600	3.110100
H	-8.367400	0.000600	2.365100
H	-7.019200	-0.883500	3.110100
O	4.025400	-0.000300	-2.657000
H	3.820000	-0.000200	-1.703600
C	5.432000	-0.000400	-2.819300
H	5.879800	0.883500	-2.339500
H	5.879700	-0.884100	-2.339000
C	5.743100	-0.000900	-4.300100
H	5.316600	0.884100	-4.784400
H	6.824700	-0.000900	-4.470300
H	5.316600	-0.886100	-4.783900
H	3.345300	-0.000000	1.971200
O	3.376400	-0.000100	2.947000
C	4.733600	-0.000300	3.350800
H	5.257700	-0.884200	2.955500
H	5.258100	0.883300	2.955200
C	4.784800	-0.000000	4.863100
H	5.821000	-0.000200	5.217200
H	4.281800	0.885100	5.266900
H	4.281400	-0.884800	5.267200

II-4S2

127

Molecule Name

Au	0.000000	0.000000	-3.219300
N	-1.084900	2.850200	-3.333000
N	1.084900	2.850200	-3.333000
C	0.000000	2.043700	-3.285800
C	0.698800	4.176800	-3.407400

C	-2.464500	2.428000	-3.199800
H	-2.493900	1.337800	-3.206600
H	-2.871400	2.801100	-2.252800
C	2.464500	2.428000	-3.199800
H	2.493900	1.337800	-3.206600
H	3.052700	2.807300	-4.040800
H	-3.052700	2.807300	-4.040800
H	2.871400	2.801100	-2.252800
N	1.084900	-2.850200	-3.333000
N	-1.084900	-2.850200	-3.333000
C	0.000000	-2.043700	-3.285800
C	-0.698800	-4.176800	-3.407400
C	2.464500	-2.428000	-3.199800
H	2.493900	-1.337800	-3.206600
H	2.871400	-2.801100	-2.252800
C	-2.464500	-2.428000	-3.199800
H	-2.493900	-1.337800	-3.206600
H	-3.052700	-2.807300	-4.040800
H	3.052700	-2.807300	-4.040800
H	-2.871400	-2.801100	-2.252800
C	-0.698800	4.176800	-3.407400
C	-1.429700	5.359200	-3.489100
C	1.429700	5.359200	-3.489100
C	-0.702200	6.540300	-3.568300
H	-2.514900	5.361100	-3.495700
C	0.702200	6.540300	-3.568300
H	2.514900	5.361100	-3.495700
H	-1.231500	7.485600	-3.637600
H	1.231500	7.485600	-3.637600
C	0.698800	-4.176800	-3.407400
C	1.429700	-5.359200	-3.489100
C	-1.429700	-5.359200	-3.489100
C	0.702200	-6.540300	-3.568300
H	2.514900	-5.361100	-3.495700
C	-0.702200	-6.540300	-3.568300
H	-2.514900	-5.361100	-3.495700
H	1.231500	-7.485600	-3.637600
H	-1.231500	-7.485600	-3.637600
N	0.000000	-3.160200	-0.000000
C	0.000000	-1.990500	-0.000000
N	0.000000	3.160200	-0.000000
C	0.000000	1.990500	-0.000000
Au	0.000000	0.000000	-0.000000
Au	0.000000	0.000000	3.219300
N	-1.084900	2.850200	3.332900
N	1.084900	2.850200	3.332900
C	0.000000	2.043700	3.285800
C	0.698800	4.176800	3.407400
C	-2.464500	2.428000	3.199800
H	-2.493900	1.337800	3.206600
H	-3.052700	2.807300	4.040800
C	2.464500	2.428000	3.199800
H	2.493900	1.337800	3.206600
H	2.871500	2.801100	2.252800
H	-2.871500	2.801100	2.252800
H	3.052700	2.807300	4.040800
N	1.084900	-2.850200	3.332900
N	-1.084900	-2.850200	3.332900
C	0.000000	-2.043700	3.285800
C	-0.698800	-4.176800	3.407400
C	2.464500	-2.428000	3.199800
H	2.493900	-1.337800	3.206600
H	3.052700	-2.807300	4.040800
C	-2.464500	-2.428000	3.199800
H	-2.493900	-1.337800	3.206600
H	-2.871500	-2.801100	2.252800
H	2.871500	-2.801100	2.252800
H	-3.052700	-2.807300	4.040800
C	-0.698800	4.176800	3.407400
C	-1.429700	5.359200	3.489100
C	-0.702200	6.540300	3.568300
H	-2.514900	5.361100	3.495600
C	1.429700	5.359200	3.489100
C	0.702200	6.540300	3.568300
H	-1.231500	7.485600	3.637600
H	2.514900	5.361100	3.495600
H	1.231500	7.485600	3.637600
C	0.698800	-4.176800	3.407400
C	1.429700	-5.359200	3.489100
C	0.702200	-6.540300	3.568300
H	2.514900	-5.361100	3.495600
C	-1.429700	-5.359200	3.489100
C	-0.702200	-6.540300	3.568300
H	1.231500	-7.485600	3.637600
H	-2.514900	-5.361100	3.495600
H	-1.231500	-7.485600	3.637600
O	2.863900	-3.839800	0.000000
H	1.892500	-3.753700	0.000000
C	3.189700	-5.217900	0.000000
H	2.764400	-5.718200	-0.883900
H	2.764400	-5.718200	0.883900
C	4.696500	-5.354700	0.000000
H	5.127600	-4.874900	0.885200
H	4.991500	-6.409200	0.000000
H	5.127600	-4.874900	-0.885100
O	-2.863900	3.839800	0.000000
H	-1.892500	3.753700	0.000000
C	-3.189700	5.217900	0.000000
H	-2.764400	5.718200	0.883900
H	-2.764400	5.718200	-0.883900
C	-4.696500	5.354700	0.000000
H	-5.127600	4.874900	0.885200
H	-4.991500	6.409200	0.000000
H	-5.127600	4.874900	-0.885100
H	1.892500	3.753700	0.000000
O	2.863900	3.839800	0.000000
C	3.189700	5.217900	0.000000
H	2.764400	5.718200	-0.883900
H	2.764400	5.718200	0.883900
C	4.696500	5.354700	0.000000
H	4.991500	6.409200	0.000000
H	5.127600	4.874900	0.885200
H	5.127600	4.874900	-0.885100
H	-1.892500	-3.753700	0.000000
O	-2.863900	-3.839800	0.000000
C	-3.189700	-5.217900	0.000000
H	-2.764400	-5.718200	0.883900
H	-2.764400	-5.718200	-0.883900
C	-4.696500	-5.354700	0.000000
H	-4.991500	-6.409200	0.000000
H	-5.127600	-4.874900	0.885200
H	-5.127600	-4.874900	-0.885100

18. The S_0 .xyz files of III and its complexes

III

Au	0.004700	-0.078400	0.002000
N	0.035400	-0.188900	3.050400
N	2.037000	-0.185800	2.274700
C	0.740200	-0.129700	1.898700
C	2.144500	-0.278800	3.643000
H	3.098200	-0.326400	4.145600
C	0.878300	-0.280200	4.133800
H	0.512600	-0.328000	5.147900
C	-1.417800	-0.179500	3.122800
H	-1.829200	-1.030400	2.573100
H	-1.714700	-0.247300	4.170600
C	3.159200	-0.183600	1.348400
H	3.126100	0.717400	0.732000
H	4.086100	-0.186900	1.924200
H	-1.803900	0.752000	2.702600
H	3.123900	-1.069300	0.707900
N	-0.027900	-0.101000	-3.048400
N	-2.028900	-0.059700	-2.272100
C	-0.731100	-0.053400	-1.895200
C	-2.138500	-0.110400	-3.642400
H	-3.093000	-0.115000	-4.145700
C	-0.872700	-0.136000	-4.133500
H	-0.508200	-0.165800	-5.148700
C	1.424900	-0.133300	-3.121000
H	1.810400	-1.011500	-2.596000
H	1.720100	-0.180100	-4.170500
C	-3.150900	-0.050200	-1.345600
H	-3.091000	0.831400	-0.703700
H	-4.077200	-0.009300	-1.920800
H	1.838700	0.773800	-2.674600
H	-3.142500	-0.954400	-0.730600
N	-3.065600	-3.109100	1.062700
C	-1.966300	-3.119900	0.661300
N	2.983000	-3.168700	-1.146000
C	1.883700	-3.157800	-0.744600
Ag	-0.041300	-3.138900	-0.041700
Au	-0.087400	-6.199400	-0.085300
N	-0.177300	-6.197100	2.964100
N	1.853500	-6.195500	2.268300
C	0.572000	-6.223600	1.839800
C	1.907400	-6.152800	3.642200
H	2.840800	-6.135700	4.183500
C	0.622700	-6.153200	4.082400
H	0.217400	-6.135300	5.082300
C	-1.632200	-6.181300	2.978700
H	-2.006400	-5.273500	2.497300
H	-2.015300	-7.059800	2.454800
C	3.011600	-6.189000	1.387500
H	3.024600	-7.100300	0.785200
H	2.983300	-5.314300	0.732100
H	-1.970500	-6.209800	4.015700
H	3.914300	-6.154000	1.999500
N	0.003800	-6.112900	-3.133500
N	-2.026200	-6.070000	-2.437000
C	-0.746400	-6.148900	-2.010000
C	-2.078300	-5.986600	-3.809100
H	-3.010400	-5.926000	-4.349300
C	-0.794000	-6.013100	-4.249700
H	-0.387900	-5.978800	-5.248800
C	1.458600	-6.140400	-3.148200
H	1.859700	-5.258400	-2.641200
H	1.814900	-7.044600	-2.649700
C	-3.184100	-6.053800	-1.555900
H	-3.224700	-6.981000	-0.979800
H	-3.129700	-5.199300	-0.875800
H	1.796300	-6.149500	-4.185700
H	-4.085000	-5.974200	-2.166300

III-S1

73

Molecule Name

N	0.007000	2.541200	1.024800
C	0.003200	1.389800	0.825400
N	-0.009700	-3.414000	-1.272400
C	-0.007500	-2.399900	-0.689600
Ag	-0.002900	-0.549800	0.176300
Au	3.535300	-0.166100	-0.023800
N	3.464500	-2.996500	1.122800
N	3.324700	-3.056400	-1.015900
C	3.444100	-2.204500	0.025000
C	3.251400	-4.357200	-0.579800
H	3.116500	-5.184500	-1.258700
C	3.349000	-4.322400	0.771600
H	3.341600	-5.115600	1.503200
C	3.603000	-2.530500	2.487900
H	3.458200	-1.449400	2.502600
H	2.841700	-3.000500	3.115600
C	3.155100	-2.668200	-2.403100
H	3.421500	-1.615200	-2.504100
H	3.814600	-3.268600	-3.034700
H	4.596900	-2.771300	2.876800
H	2.110700	-2.818400	-2.689300
N	2.990600	2.730800	-0.849600
N	4.340200	2.661800	0.813500
C	3.640800	1.872500	-0.034900
C	4.118000	3.993100	0.540400
H	4.574800	4.783900	1.115300
C	3.263200	4.034900	-0.511000
H	2.811300	4.863400	-1.032600
C	2.130300	2.356000	-1.956100
H	1.705200	1.371800	-1.753700
H	1.328200	3.093500	-2.026100
C	5.170900	2.189600	1.903600
H	5.342900	1.120600	1.771200
H	6.131200	2.711200	1.887700
H	2.702500	2.327900	-2.888500
H	4.675600	2.363700	2.863200
Au	-3.535700	-0.153000	-0.024500
N	-3.484700	-2.988200	1.111500
N	-3.342000	-3.040900	-1.027200
C	-3.457600	-2.192200	0.016800
C	-3.277500	-4.343700	-0.595900
H	-3.146800	-5.169300	-1.277800
C	-3.376900	-4.313500	0.755400
H	-3.375600	-5.109500	1.484000
C	-3.622200	-2.526700	2.478200
H	-3.473400	-1.446100	2.496700
H	-4.617200	-2.765100	2.865700
C	-3.167200	-2.648400	-2.412500
H	-3.429600	-1.594000	-2.510600

H -2.122700 -2.801300 -2.696600
H -2.863000 -3.001600 3.104600
H -3.827200 -3.244200 -3.048000
N -2.976700 2.743800 -0.841600
N -4.319400 2.676400 0.827000
C -3.628600 1.886200 -0.027600
C -4.090100 4.007400 0.558300
H -4.539400 4.798600 1.138400
C -3.239600 4.048100 -0.496700
H -2.784600 4.875900 -1.016800
C -2.123600 2.368100 -1.953400
H -1.702100 1.381300 -1.755800
H -2.700300 2.345200 -2.883100
C -5.147300 2.204800 1.919500
H -5.333900 1.139200 1.779100
H -4.642300 2.364100 2.876700
H -1.318400 3.102100 -2.025300
H -6.100900 2.738600 1.915800
O 0.011000 4.663000 -0.813500
H 0.009800 4.006700 -0.080200
C 0.017500 5.950900 -0.242700
H 0.907800 6.128300 0.378900
H 0.019900 6.679700 -1.059100
H -0.869900 6.136500 0.380700

III-2S1

79
Molecule Name
Au -3.435200 -0.000200 0.000000
N -3.009800 2.949400 0.714700
N -3.925400 2.759900 -1.214100
C -3.461100 2.034600 -0.170400
C -3.754100 4.106700 -0.989400
H -4.046000 4.853800 -1.711300
C -3.174200 4.224800 0.230200
H -2.852300 5.091100 0.785800
C -2.402600 2.657900 2.000100
H -2.050400 1.625100 1.994300
H -1.557100 3.335700 2.142400
C -4.471800 2.208800 -2.438700
H -4.651100 1.143100 -2.290200
H -5.417300 2.702500 -2.677200
H -3.132600 2.790900 2.804400
H -3.766700 2.347400 -3.263100
N -3.009200 -2.949800 -0.714700
N -3.924800 -2.760500 1.214200
C -3.460700 -2.035100 0.170400
C -3.753100 -4.107200 0.989500
H -4.044900 -4.854400 1.711300
C -3.173300 -4.225200 -0.230100
H -2.851200 -5.091500 -0.785800
C -2.402100 -2.658200 -2.000100
H -2.050200 -1.625300 -1.994400
H -1.556500 -3.335800 -2.142500
C -4.471400 -2.209500 2.438700
H -4.651000 -1.143800 2.290200
H -5.416900 -2.703300 2.677200
H -3.132200 -2.791400 -2.804400
H -3.766300 -2.348000 3.263200
N 0.000300 -3.064100 0.955300
C 0.000200 -1.928100 0.679800
N -0.000400 3.064100 -0.955300
C -0.000200 1.928100 -0.679800
Ag 0.000000 -0.000000 -0.000000
Au 3.435200 0.000200 0.000000
N 3.009200 2.949800 0.714700
N 3.924800 2.760400 -1.214100
C 3.460700 2.035100 -0.170400
C 3.753200 4.107200 -0.989400
H 4.044900 4.854400 -1.711300
C 3.173300 4.225200 0.230200
H 2.851200 5.091500 0.785800
C 2.402000 2.658200 2.000100
H 2.050000 1.625400 1.994300
H 3.132000 2.791300 2.804400
C 4.471500 2.209500 -2.438600
H 4.651000 1.143800 -2.290100
H 3.766400 2.348000 -3.263100
H 1.556400 3.335900 2.142400
H 5.417000 2.703300 -2.677000
N 3.009800 -2.949400 -0.714800
N 3.925300 -2.759900 1.214200
C 3.461100 -2.034600 0.170400
C 3.753900 -4.106700 0.989400
H 4.045800 -4.853800 1.711300
C 3.174100 -4.224800 -0.230200
H 2.852200 -5.091100 -0.785800
C 2.402700 -2.657900 -2.000200
H 2.050600 -1.625100 -1.994500
H 3.132800 -2.791000 -2.804500
C 4.471700 -2.208800 2.438700
H 4.651200 -1.143100 2.290200
H 3.766600 -2.347300 3.263100
H 1.557200 -3.335600 -2.142600
H 5.417200 -2.702600 2.677300
O 0.000500 -4.900000 -1.184400
H 0.000400 -4.370900 -0.356900
C 0.000500 -6.265400 -0.840800
H -0.887200 -6.553600 -0.257700
H 0.000600 -6.843400 -1.769500
H 0.888200 -6.553600 -0.257600
O -0.000400 4.900100 1.184300
H -0.000400 4.370900 0.356900
C -0.000500 6.265400 0.840600
H -0.000500 6.843600 1.769100
H 0.887100 6.553600 0.257200
H -0.888300 6.553500 0.257300

III-3S1

85
Molecule Name
Au -3.313800 0.413400 0.007600
N -3.518700 -2.526500 -0.837600
N -3.637700 -2.338200 1.296400
C -3.498100 -1.615900 0.162100
C -3.734200 -3.679900 1.012400
H -3.851000 -4.424700 1.784500
C -3.659400 -3.798400 -0.336300
H -3.699000 -4.667400 -0.974900
C -3.295700 -2.243300 -2.243700
H -3.165100 -1.166800 -2.361800
H -2.389000 -2.757900 -2.578200
C -3.567000 -1.809500 2.647400
H -3.628100 -0.721500 2.595800

H -4.407000 -2.188000 3.235700
H -4.155400 -2.572100 -2.834400
H -2.617300 -2.110800 3.101700
N -2.583200 3.296700 0.721500
N -3.660700 3.231400 -1.130100
C -3.182200 2.444900 -0.138400
C -3.353900 4.553000 -0.898200
H -3.631700 5.338700 -1.583800
C -2.671200 4.592200 0.272400
H -2.224600 5.412400 0.811800
C -1.923000 2.921300 1.958300
H -1.557000 1.897300 1.864400
H -1.081100 3.600000 2.115400
C -4.351000 2.758600 -2.313900
H -4.625300 1.713900 -2.162000
H -5.258000 3.347400 -2.472800
H -2.622100 2.985100 2.797700
H -3.701500 2.841600 -3.190000
N 0.442600 3.460100 -0.992800
C 0.297900 2.301600 -0.945900
N -0.342100 -2.807800 0.110900
C -0.204600 -1.678600 -0.165600
Ag 0.046300 0.301000 -0.613200
Au 3.327900 -0.228800 0.050400
N 2.876800 -3.062800 -1.014600
N 2.944500 -3.065600 1.129800
C 3.031900 -2.250000 0.055000
C 2.726600 -4.365700 0.739100
H 2.622300 -5.170500 1.450500
C 2.684400 -4.363700 -0.616300
H 2.534600 -5.163700 -1.324400
C 2.831800 -2.640300 -2.402700
H 2.822000 -1.549800 -2.431400
H 3.709300 -3.011200 -2.940500
C 2.941200 -2.633900 2.516500
H 3.294200 -1.602500 2.558200
H 1.924000 -2.697700 2.918300
H 1.916300 -3.029700 -2.857400
H 3.617100 -3.265900 3.098700
N 3.238300 2.706500 0.923100
N 4.350100 2.490000 -0.896300
C 3.667200 1.784300 0.034600
C 4.336000 3.834000 -0.600000
H 4.811300 4.568900 -1.231100
C 3.631800 3.969400 0.550500
H 3.359800 4.842000 1.122800
C 2.455000 2.433900 2.113600
H 1.919800 1.493200 1.973600
H 3.106400 2.357100 2.989500
C 4.954000 1.933100 -2.090800
H 5.026400 0.851100 -1.972500
H 4.342300 2.163900 -2.967700
H 1.740000 3.249500 2.246300
H 5.956600 2.346900 -2.223500
O 0.544200 5.076300 1.300200
H 0.533300 4.626400 0.425900
C 0.695500 6.460900 1.087200
H -0.125600 6.887000 0.491400
H 1.639500 6.707100 0.578200
H 0.697100 6.951000 2.065000
O -0.229200 -4.160600 -2.447500
H -0.261000 -3.827300 -1.530400
C -0.511800 -5.539000 -2.434100
H 0.222000 -6.117300 -1.851500
H -1.510800 -5.762500 -2.029300
H -0.479700 -5.893200 -3.467900
C -0.558400 -4.650400 3.261700
O -0.456100 -3.282400 2.947800
H 0.309700 -5.230700 2.912400
H -1.463000 -5.114100 2.839800
H -0.433800 -3.195800 1.974000
H -0.607200 -4.738400 4.350500

III-4S1

91
Molecule Name
Au -3.224800 -0.021700 -0.000000
N -3.392700 2.973200 0.596700
N -3.095200 2.644700 -1.502400
C -3.248500 1.995200 -0.326400
C -3.132400 4.005900 -1.317800
H -3.022500 4.699100 -2.137100
C -3.319000 4.213700 0.009100
H -3.402100 5.126700 0.578300
C -3.463000 2.768400 2.032300
H -3.721500 1.725500 2.221300
H -2.494300 2.998900 2.489300
C -2.835300 2.023800 -2.788700
H -2.705200 0.951800 -2.634700
H -3.676100 2.193600 -3.467600
H -4.240100 3.409900 2.455600
H -1.917700 2.450200 -3.205300
N -3.351800 -3.018800 -0.596700
N -3.058900 -2.686100 1.502400
C -3.221000 -2.038900 0.326400
C -3.077600 -4.047700 1.317900
H -2.958300 -4.739400 2.137300
C -3.261300 -4.258100 -0.009000
H -3.332000 -5.172200 -0.578200
C -3.424700 -2.815000 -2.032300
H -3.697000 -1.775700 -2.221400
H -2.452900 -3.032600 -2.489200
C -2.807600 -2.061800 2.788700
H -2.692000 -0.988100 2.634600
H -3.646000 -2.242900 3.467600
H -4.193100 -3.466900 -2.455600
H -1.884200 -2.475600 3.205300
N 0.021900 -3.218600 0.000000
C 0.014000 -2.047500 -0.000000
N -0.021900 3.218700 0.000000
C -0.013900 2.047600 -0.000100
Ag 0.000000 0.000000 -0.000100
Au 3.224800 0.021700 0.000000
N 3.058900 2.686100 1.502400
N 3.351800 3.018800 -0.596700
C 3.221000 2.038900 0.326400
C 3.261300 4.258100 -0.009000
H 3.332100 5.172100 -0.578200
C 3.077700 4.047700 1.317900
H 2.958400 4.739400 2.137300
C 2.807500 2.061800 2.788800
H 2.691900 0.988100 2.634700
H 3.645900 2.242900 3.467700
C 3.424700 2.815000 -2.032200
H 3.697100 1.775700 -2.221400
H 2.453000 3.032600 -2.489100
H 1.884200 2.475600 3.205300

H 4.193200 3.466900 -2.455600
N 3.095200 -2.644600 -1.502400
N 3.392700 -2.973300 0.596700
C 3.248500 -1.995200 -0.326400
C 3.319000 -4.213700 0.009000
H 3.402200 -5.126700 0.578300
C 3.132400 -4.005900 -1.317800
H 3.022500 -4.699100 -2.137200
C 2.835300 -2.023800 -2.788700
H 2.705200 -0.951700 -2.634600
H 3.676100 -2.193600 -3.467600
C 3.462900 -2.768500 2.032300
H 3.721400 -1.725600 2.221400
H 2.494200 -2.999000 2.489300
H 1.917700 -2.450100 -3.205300
H 4.240100 -3.409900 2.455600
O -0.067700 -3.848100 -2.843800
H -0.019500 -3.736800 -1.875500
C -0.178600 -5.223500 -3.124800
H -1.092600 -5.668000 -2.702000
H 0.680700 -5.801900 -2.752400
H -0.219600 -5.336700 -4.211400
O -0.120100 3.846700 2.843900
H -0.070400 3.736100 1.875600
C -0.249500 5.220500 3.124900
H 0.601800 5.810500 2.752400
H -1.169500 5.652500 2.702300
H -0.292000 5.333100 4.211500
C 0.178600 5.223500 -3.124900
O 0.067700 3.848100 -2.843800
H 1.092600 5.668000 -2.702100
H -0.680700 5.801900 -2.752600
H 0.019500 3.736800 -1.875500
H 0.219700 5.336600 -4.211500
C 0.249400 -5.220600 3.124800
O 0.119900 -3.846800 2.843800
H 1.169400 -5.652600 2.702300
H -0.601900 -5.810600 2.752300
H 0.070300 -3.736200 1.875500
H 0.291700 -5.333200 4.211400

III-S2

76
Molecule Name
N -0.215400 -3.462600 -1.411700
C -0.158300 -2.473200 -0.790400
N 0.169800 2.383900 1.125000
C 0.089800 1.244800 0.876700
Ag -0.042700 -0.662600 0.149000
Au 3.499100 -0.448900 0.008000
N 3.152100 2.482600 -0.808700
N 4.504100 2.313100 0.845500
C 3.744300 1.578000 0.000000
C 4.377300 3.657400 0.575800
C 3.520800 3.762800 -0.469700
C 2.254300 2.174800 -1.906300
C 5.305500 1.779800 1.929300
N 3.214500 -3.266000 1.155000
N 3.132000 -3.323500 -0.986900
C 3.275000 -2.477600 0.056000
C 2.965100 -4.615900 -0.551300
C 3.025600 -4.583100 0.802400
C 3.334700 -2.804700 2.523500
C 3.035500 -2.931000 -2.379900
H 4.894400 4.412100 1.148300
H 3.131300 4.623700 -0.989300
H 1.789600 1.205900 -1.718400
H 1.484700 2.948800 -1.941500
H 2.806900 2.143600 -2.850400
H 5.387800 0.699700 1.800800
H 6.305000 2.221000 1.901000
H 4.835800 1.997900 2.892800
H 2.800200 -5.435400 -1.233100
H 2.948000 -5.372100 1.534500
H 3.321600 -1.713900 2.523900
H 2.490600 -3.171700 3.112900
H 4.270800 -3.158400 2.965700
H 3.344200 -1.888400 -2.467800
H 3.698300 -3.555500 -2.984100
H 1.999000 -3.041500 -2.709600
Au -3.540700 -0.116700 0.001400
N -2.828500 2.790200 -0.639800
N -4.087500 2.687100 1.091100
C -3.494200 1.918900 0.147900
C -3.784600 4.017000 0.902000
C -2.987200 4.080100 -0.192400
C -2.053500 2.441500 -1.815900
C -4.886600 2.190600 2.193700
N -3.709200 -3.028500 0.915800
N -3.520900 -2.926300 -1.217300
C -3.602900 -2.154300 -0.112400
C -3.554200 -4.259800 -0.888300
C -3.682200 -4.326800 0.459200
C -3.840000 -2.666100 2.312500
C -3.292300 -2.441300 -2.564900
H -4.150000 4.790900 1.559300
H -2.504400 4.913000 -0.678200
H -1.674800 1.424800 -1.699100
H -2.676400 2.503800 -2.713400
H -1.215300 3.137600 -1.887500
H -5.146800 1.150400 1.992900
H -4.324100 2.252200 3.129700
H -5.803900 2.778800 2.278300
H -3.465900 -5.037600 -1.630600
H -3.751300 -5.174600 1.123200
H -3.678300 -1.591600 2.408000
H -4.838300 -2.918800 2.681900
H -3.087300 -3.194200 2.903500
H -3.470600 -1.365200 -2.580700
H -2.257600 -2.651300 -2.848300
H -3.984900 -2.931500 -3.253600
C 0.395800 5.825700 -0.101000
C 0.441200 6.857700 -1.207700
O 0.262700 4.542000 -0.678500
H 1.312100 5.885600 0.507700
H -0.447200 6.037900 0.576200
H -0.473500 6.822800 -1.809700
H 0.543600 7.866700 -0.795700
H 1.287500 6.672100 -1.878500
H 0.240000 3.877400 0.047000

III-2S2

85
Molecule Name
Au 3.446000 0.514200 -0.030200

N	2.542400	3.426000	-0.337600
N	3.510100	3.128300	1.551700
C	3.150400	2.485100	0.416600
C	3.119600	4.447800	1.511900
H	3.300100	5.128100	2.330000
C	2.506400	4.633800	0.317000
H	2.036200	5.502400	-0.116500
C	1.975600	3.216900	-1.657200
H	1.730700	2.159700	-1.769900
H	1.064600	3.815000	-1.737600
C	4.164500	2.513700	2.690300
H	4.506100	1.519200	2.399400
H	5.025900	3.115600	2.991600
H	2.691600	3.513100	-2.430000
H	3.465700	2.427700	3.527600
N	3.577100	-2.533700	0.291100
N	4.330600	-1.934100	-1.625100
C	3.802900	-1.446400	-0.477800
C	4.422400	-3.306000	-1.577800
H	4.804000	-3.888700	-2.402100
C	3.945000	-3.682900	-0.366600
H	3.829600	-4.659900	0.076000
C	2.982700	-2.526000	1.615600
H	2.555600	-1.538400	1.796900
H	2.189400	-3.278700	1.657400
C	4.705100	-1.139900	-2.778400
H	4.660400	-0.085200	-2.502100
H	5.724400	-1.388100	-3.085700
H	3.744300	-2.738000	2.372100
H	4.015500	-1.328500	-3.606300
N	0.543300	-3.195800	-1.122200
C	0.360600	-2.079100	-0.827400
N	-0.460900	2.759800	1.050000
C	-0.272400	1.663100	0.693400
Ag	0.049300	-0.200200	-0.083500
Au	-3.463600	-0.446300	-0.064900
N	-3.432600	2.577100	-0.568700
N	-4.459000	2.122700	1.257600
C	-3.812200	1.546700	0.217400
C	-4.471900	3.493200	1.128500
H	-4.928900	4.137800	1.863600
C	-3.820600	3.778800	-0.026000
H	-3.584800	4.719000	-0.498500
C	-2.697800	2.465800	-1.815300
H	-2.173800	1.509100	-1.828300
H	-3.383700	2.521500	-2.666400
C	-5.025800	1.413300	2.388100
H	-4.972500	0.342600	2.186600
H	-4.461900	1.639700	3.297600
H	-1.972600	3.281900	-1.861200
H	-6.071700	1.702600	2.522600
N	-2.565300	-3.315000	0.512000
N	-3.475100	-3.184500	-1.425100
C	-3.152500	-2.445000	-0.338700
C	-3.083700	-4.493700	-1.259500
H	-3.232500	-5.240900	-2.023800
C	-2.507800	-4.574400	-0.034500
H	-2.028200	-5.392200	0.479600
C	-2.050200	-2.992200	1.830600
H	-1.823700	-1.925200	1.868300
H	-2.792700	-3.234400	2.597200
C	-4.099400	-2.674600	-2.630200
H	-4.415500	-1.646300	-2.448600
H	-3.388600	-2.694900	-3.461500
H	-1.138000	-3.570400	1.995900
H	-4.974200	-3.281100	-2.880400
O	0.534300	-4.956900	1.096800
H	0.565800	-4.492300	0.232800
C	1.032600	-6.271100	0.943000
H	2.101100	-6.258000	0.668100
H	0.505200	-6.799500	0.132600
C	0.854200	-7.012500	2.249900
H	1.255000	-8.029000	2.179900
H	1.373400	-6.489700	3.060600
H	-0.206800	-7.077700	2.514900
O	-0.720300	4.979500	-0.646900
H	-0.653000	4.298000	0.058800
C	-0.947500	6.236500	-0.042000
H	-1.868400	6.219100	0.563600
H	-0.126900	6.495100	0.647700
C	-1.061000	7.287800	-1.125500
H	-1.891100	7.057200	-1.803000
H	-1.234700	8.277500	-0.689700
H	-0.143100	7.329300	-1.722900

III-3S2

94

Molecule Name

Au	-0.402400	3.318700	-0.078200
N	-3.324200	2.730700	-0.790800
N	-3.089500	2.948300	1.330800
C	-2.401600	2.975800	0.167200
C	-4.419100	2.679800	1.106000
H	-5.136600	2.613000	1.909300
C	-4.566900	2.542800	-0.234800
H	-5.439900	2.334200	-0.833900
C	-3.049100	2.560800	-2.205900
H	-1.980100	2.704700	-2.368300
H	-3.329300	1.547300	-2.511200
C	-2.507500	3.054000	2.657200
H	-1.478400	3.401700	2.556000
H	-3.072900	3.777400	3.250700
H	-3.607700	3.298900	-2.788700
H	-2.526700	2.069900	3.137000
N	2.600600	3.365700	0.503000
N	2.178600	4.349600	-1.353600
C	1.586100	3.708700	-0.319800
C	3.543900	4.394200	-1.184700
H	4.200800	4.846900	-1.911200
C	3.809100	3.770200	-0.010700
H	4.739700	3.556700	0.490600
C	2.463000	2.660100	1.763700
H	1.565500	2.040200	1.723500
H	3.342800	2.026100	1.897800
C	1.492500	4.868000	-2.520700
H	0.421600	4.889400	-2.313900
H	1.836800	5.883900	-2.729900
H	2.379900	3.371300	2.591200
H	1.684000	4.229800	-3.388100
N	3.467600	0.463800	-1.212400
C	2.314900	0.301800	-1.110400
N	-2.728300	-0.388000	0.203300
C	-1.616600	-0.234500	-0.129700
Ag	0.335700	0.028900	-0.679000
Au	0.690600	-3.273400	0.009700
N	-2.216000	-3.594400	-0.895000

N	-2.088800	-3.593900	1.246700
C	-1.335700	-3.503400	0.127300
C	-3.419700	-3.728100	0.929600
H	-4.186000	-3.809200	1.685000
C	-3.499800	-3.728900	-0.424100
H	-4.348000	-3.810600	-1.085700
C	-1.894000	-3.480100	-2.306000
H	-0.840700	-3.211800	-2.399400
H	-2.073100	-4.432500	-2.813700
C	-1.600800	-3.434600	2.605100
H	-0.511400	-3.491200	2.589400
H	-1.918900	-2.462200	2.996800
H	-2.515200	-2.693500	-2.744600
H	-1.991100	-4.240300	3.232500
N	3.540200	-2.406500	0.717800
N	3.533300	-3.589600	-1.069200
C	2.719600	-3.084500	-0.113300
C	4.840300	-3.221300	-0.844800
H	5.642600	-3.509300	-1.506500
C	4.842700	-2.472100	0.284900
H	5.641400	-1.967200	0.804800
C	3.129700	-1.688300	1.910000
H	2.084800	-1.393500	1.799400
H	3.239200	-2.324000	2.794100
C	3.098300	-4.361400	-2.216600
H	2.063500	-4.667500	-2.057400
H	3.164300	-3.757800	-3.126200
H	3.755900	-0.797900	2.004900
H	3.723300	-5.252000	-2.321000
O	5.157900	0.830900	1.008100
H	4.689600	0.709900	0.151900
C	6.536000	1.021300	0.750800
H	6.692900	1.881900	0.080900
H	6.963100	0.143800	0.238800
C	7.250600	1.251000	2.064900
H	7.117100	0.393000	2.732300
H	8.323200	1.398500	1.903400
H	6.853100	2.136900	2.571600
O	-4.099400	-0.863600	-2.323100
H	-3.754300	-0.738900	-1.418400
C	-5.508500	-0.953500	-2.265400
H	-5.824400	-1.792200	-1.623100
H	-5.943300	-0.040600	-1.825600
C	-6.039300	-1.150200	-3.668400
H	-7.131700	-1.221700	-3.664700
H	-5.748200	-0.312100	-4.309800
H	-5.634200	-2.067800	-4.107600
C	-4.461300	-0.580000	3.438200
O	-3.127900	-0.310100	3.056900
H	-4.780400	-1.572300	3.077700
H	-5.154200	0.152800	2.992800
H	-3.077600	-0.331800	2.080300
C	-4.555800	-0.528600	4.947400
H	-4.268200	0.460800	5.318200
H	-5.577000	-0.739400	5.281100
H	-3.884400	-1.265800	5.399800

III-4S2

103

Molecule Name

Au	0.000700	3.239700	-0.000600
N	2.867600	3.265700	1.073700
N	2.868700	3.266600	-1.071800
C	2.044400	3.262700	0.000500
C	4.184900	3.261800	-0.676200
H	4.998800	3.258900	-1.384900
C	4.184200	3.261300	0.679500
H	4.997300	3.257800	1.389000
C	2.451200	3.164700	2.460800
H	1.363000	3.227000	2.498800
H	2.781600	2.203900	2.870000
C	2.453800	3.166300	-2.459500
H	1.365700	3.229900	-2.498600
H	2.882200	3.990900	-3.035900
H	2.878000	3.989700	3.037900
H	2.783500	2.205200	-2.868400
N	-2.866300	3.265000	-1.074800
N	-2.867300	3.267200	1.070700
C	-2.043100	3.262700	-0.001700
C	-4.183500	3.262200	0.675100
H	-4.997400	3.259500	1.383800
C	-4.182900	3.260900	-0.680600
H	-4.996000	3.256800	-1.390100
C	-2.449900	3.163900	-2.462000
H	-1.361600	3.225200	-2.499800
H	-2.781100	2.203500	-2.871300
C	-2.452300	3.168200	2.458400
H	-1.364200	3.230200	2.497300
H	-2.879400	3.994200	3.033800
H	-2.875900	3.989400	-3.038900
H	-2.783300	2.208100	2.868800
N	-3.221800	0.000100	0.002900
C	-2.050700	0.000100	0.001900
N	3.215500	-0.000100	-0.001400
C	2.044400	-0.000100	-0.000500
Ag	-0.003200	0.000000	0.000700
Au	0.000300	-3.239700	-0.000600
N	2.867300	-3.265900	1.073700
N	2.868400	-3.266700	-1.071800
C	2.044100	-3.262800	0.000500
C	4.184600	-3.262100	-0.676200
H	4.998400	-3.259200	-1.384900
C	4.183900	-3.261600	0.679500
H	4.997000	-3.258200	1.389000
C	2.450900	-3.164900	2.460800
H	1.362700	-3.227100	2.498800
H	2.877600	-3.989900	3.037900
C	2.453400	-3.166500	-2.459400
H	1.365400	-3.230000	-2.498600
H	2.783300	-2.205400	-2.868400
H	2.781400	-2.204100	2.870000
H	2.881800	-3.991100	-3.035900
N	-2.866600	-3.264800	-1.074800
N	-2.867700	-3.267000	1.070700
C	-2.043400	-3.262600	-0.001700
C	-4.183900	-3.261900	0.675100
H	-4.997700	-3.259100	1.383800
C	-4.183200	-3.260600	-0.680600
H	-4.996300	-3.256500	-1.390100
C	-2.450100	-3.163800	-2.461900
H	-1.361900	-3.225200	-2.499800
H	-2.876200	-3.989200	-3.038900
C	-2.452700	-3.168000	2.458400
H	-1.364500	-3.230100	2.497300
H	-2.783600	-2.207900	2.868800
H	-2.781300	-2.203300	-2.871300

H	-2.879800	-3.994000	3.033800
O	-3.853000	0.000100	-2.848800
H	-3.741400	0.000100	-1.878800
C	-5.236600	0.000200	-3.140200
H	-5.729500	0.884300	-2.703400
H	-5.729600	-0.884000	-2.703500
C	-5.415500	0.000200	-4.642300
H	-4.948000	-0.885400	-5.085000
H	-6.477700	0.000200	-4.907700
H	-4.947900	0.885800	-5.085000
O	3.850300	-0.000200	2.849400
H	3.737600	-0.000200	1.879600
C	5.234300	-0.000200	3.139300
H	5.726600	-0.884400	2.702000
H	5.726700	0.883900	2.702000
C	5.414800	-0.000200	4.641200
H	6.477300	-0.000200	4.905400
H	4.947800	0.885400	5.084400
H	4.947700	-0.885800	5.084400
C	5.232100	-0.000300	-3.143300
O	3.848600	-0.000200	-2.851600
H	5.725000	-0.884400	-2.706500
H	5.725200	0.883900	-2.706500
H	3.737000	-0.000200	-1.881600
C	5.410900	-0.000300	-4.645400
H	4.943300	0.885300	-5.088000
H	6.473000	-0.000300	-4.910900
H	4.943200	-0.885800	-5.088000
C	-5.229100	0.000300	3.150400
O	-3.846000	0.000100	2.856700
H	-5.722800	-0.883800	2.714400
H	-5.722600	0.884500	2.714300
H	-3.736000	0.000100	1.886500
C	-5.405600	0.000400	4.652700
H	-6.467300	0.000600	4.919800
H	-4.937400	-0.885200	5.094700
H	-4.937200	0.886000	5.094600

19. The T_1 .xyz files of I and its complexes

I

67
Molecule Name

Au	2.767000	0.000000	0.000000
N	2.863400	-2.868900	1.031100
N	2.883200	-2.832400	-1.122500
C	2.856900	-2.025900	-0.031900
C	2.921200	-4.155900	-0.746000
H	2.957300	-4.958700	-1.466200
C	2.909100	-4.178900	0.610800
H	2.933200	-5.005600	1.303900
C	2.866700	-2.435200	2.416300
H	2.008800	-1.786800	2.612100
H	2.806500	-3.314800	3.059300
C	2.907400	-2.351500	-2.491900
H	3.821700	-1.777500	-2.669300
H	2.889800	-3.209800	-3.165300
N	0.001000	-0.006500	3.161400
C	0.000700	-0.003900	1.991000
H	3.791900	-1.894500	2.637100
H	2.036000	-1.721300	-2.687800
N	2.862900	2.868900	-1.031200
N	2.882800	2.832500	1.122400
C	2.856600	2.026000	0.031900
C	2.920500	4.156000	0.745900
H	2.956500	4.958800	1.466000
C	2.908300	4.178900	-0.610900
H	2.932300	5.005600	-1.304000
C	2.866100	2.435100	-2.416300
H	2.008000	1.786900	-2.612100
H	2.806100	3.314700	-3.059300
C	2.907200	2.351700	2.491900
H	3.821600	1.777800	2.669200
H	2.889800	3.210100	3.165300
N	0.000500	0.006400	-3.161600
C	0.000400	0.003700	-1.991200
H	3.791100	1.894200	-2.637000
H	2.035900	1.721600	2.688100
Au	-2.766100	0.000000	0.000100
N	-2.887600	-2.832500	1.121400
N	-2.859600	-2.868900	-1.032100
C	-2.856500	-2.025900	0.031000
C	-2.908900	-4.178900	-0.611900
H	-2.931400	-5.005600	-1.305200
C	-2.925800	-4.155900	0.744700
H	-2.964300	-4.958700	1.464800
C	-2.914700	-2.351700	2.490800
H	-2.041900	-1.724600	2.690000
H	-3.827800	-1.774700	2.665200
C	-2.862800	-2.435600	-2.417300
H	-3.805800	-1.932400	-2.651400
H	-2.028500	-1.754100	-2.601000
H	-2.902500	-3.210300	3.164100
H	-2.757600	-3.311700	-3.059300
N	-2.887200	2.832500	-1.121300
N	-2.858900	2.869000	1.032200
C	-2.856100	2.026000	-0.030800
C	-2.908000	4.179000	0.612000
H	-2.930200	5.005700	1.305300
C	-2.925100	4.155900	-0.744600
H	-2.963500	4.958700	-1.464700
C	-2.914800	2.351700	-2.490700
H	-2.042200	1.724300	-2.689900
H	-3.828000	1.775100	-2.664900
C	-2.862100	2.435800	2.417400
H	-3.805500	1.933400	2.651900
H	-2.028300	1.753500	2.601000
H	-2.902300	3.210300	-3.164000
H	-2.755900	3.311900	3.059400
Au	0.000500	-0.000100	-0.000100

I-S1

73
Molecule Name

Au	2.767500	-0.138400	-0.150900
N	2.962300	-2.639100	1.587600
N	3.004400	-3.157900	-0.502400
C	2.935100	-2.098900	0.342900
C	3.090300	-4.337000	0.202700
H	3.162000	-5.296400	-0.286000
C	3.065000	-4.010600	1.519400
H	3.112800	-4.629500	2.402100
C	2.944500	-1.863600	2.813000
H	2.123400	-1.142800	2.786000
H	2.796800	-2.540300	3.656400
C	3.025000	-3.045400	-1.949300
H	3.931400	-2.523300	-2.270400
H	3.020400	-4.048300	-2.379200
N	0.000200	0.774900	2.901800
C	0.000100	0.385200	1.798200
H	3.893600	-1.334000	2.940800
H	2.144000	-2.500900	-2.298100
N	2.758300	2.342200	-1.915500
N	2.776400	2.907700	0.162600
C	2.787700	1.829900	-0.659100
C	2.751400	4.073900	-0.567300
H	2.740900	5.045100	-0.097000
C	2.743300	3.718200	-1.876600
H	2.731600	4.319400	-2.772800
C	2.795400	1.541900	-3.125700
H	2.013500	0.779100	-3.096900
H	2.624300	2.194700	-3.983300
C	2.818700	2.858300	1.613200
H	3.753300	3.301800	1.970200
H	1.958800	3.384700	2.039900
N	-0.000300	-1.099400	-3.136200
C	-0.000100	-0.769500	-2.013100
H	3.773300	1.062500	-3.231500
H	2.775400	1.813600	1.922900
Au	-2.767500	-0.137800	-0.150800
N	-2.963000	-2.638300	1.588000
N	-3.005400	-3.157200	-0.501900
C	-2.935700	-2.098200	0.343200
C	-3.091700	-4.336200	0.203300
H	-3.163800	-5.295700	-0.285300
C	-3.066300	-4.009700	1.519900
H	-3.114300	-4.628600	2.402700
C	-2.944900	-1.862700	2.813300
H	-2.124100	-1.141700	2.785900
H	-3.894100	-1.333400	2.941600
C	-3.026100	-3.044900	-1.948900

H -3.932300 -2.522600 -2.270000
H -2.144900 -2.500600 -2.297700
H -2.796600 -2.539300 3.656700
H -3.021600 -4.047800 -2.378700
N -2.757800 2.342500 -1.915800
N -2.775500 2.908400 0.162200
C -2.787200 1.830400 -0.659300
C -2.750400 4.074400 -0.567900
H -2.739700 5.045700 -0.097800
C -2.742500 3.718500 -1.877100
H -2.730800 4.319500 -2.773400
C -2.795500 1.542100 -3.125800
H -3.774000 1.063900 -3.232000
H -2.623200 2.194500 -3.983500
C -2.817500 2.859200 1.612900
H -3.751700 3.303400 1.970000
H -2.774800 1.814600 1.922800
H -2.014500 0.778300 -3.096600
H -1.957200 3.385300 2.039300
Au 0.000000 -0.201600 -0.105400
O 0.000700 3.651200 3.178700
H 0.000400 2.677100 3.253200
C 0.000700 4.194400 4.482000
H 0.891500 3.901400 5.055500
H 0.001500 5.283300 4.383300
H -0.890900 3.902600 5.055000

I-2S1

79
Molecule Name
Au -2.847800 0.003400 -0.000200
N -2.774300 -2.934100 -0.902400
N -3.437100 -2.751500 1.133500
C -3.033500 -2.022800 0.064700
C -3.430700 -4.095400 0.837700
H -3.714100 -4.845600 1.559500
C -3.017800 -4.209700 -0.448300
H -2.871500 -5.076400 -1.072700
C -2.269800 -2.631900 -2.225600
H -1.834100 -1.630000 -2.207800
H -1.493400 -3.360500 -2.470700
C -3.726100 -2.198200 2.439400
H -4.205300 -1.223500 2.316800
H -4.408800 -2.865200 2.970000
H -3.074500 -2.666900 -2.966700
H -2.802800 -2.085300 3.015500
N -2.767000 2.940300 0.902700
N -3.430900 2.760100 -1.133000
C -3.028500 2.030000 -0.064700
C -3.421400 4.103800 -0.836600
H -3.703600 4.854900 -1.558000
C -3.007800 4.216600 0.449300
H -2.859400 5.082700 1.073900
C -2.262500 2.636500 2.225500
H -1.829500 1.633400 2.207300
H -1.483900 3.362900 2.470400
C -3.721700 2.207900 -2.439100
H -4.202400 1.233900 -2.316600
H -4.403700 2.876300 -2.968800
H -3.066600 2.673500 2.967100
H -2.799000 2.093800 -3.015800
N -0.017500 2.922000 -1.202500
C -0.010300 1.820700 -0.804700
N -0.025100 -2.922300 1.202800
C -0.015200 -1.820900 0.805100
Au -0.004700 -0.000100 0.000200
Au 2.841200 -0.003300 0.000100
N 2.873400 -2.987500 -0.720800
N 3.318500 -2.677400 1.358800
C 3.024000 -2.017800 0.212200
C 3.353200 -4.036000 1.144300
H 3.564000 -4.738800 1.935400
C 3.077900 -4.231100 -0.168600
H 3.006200 -5.134900 -0.752200
C 2.515200 -2.769100 -2.106500
H 2.098300 -1.764000 -2.201100
H 3.392800 -2.861800 -2.754000
C 3.458100 -2.040600 2.650700
H 4.028700 -1.115000 2.538600
H 2.471700 -1.818800 3.069400
H 1.755000 -3.503000 -2.384400
H 3.997200 -2.710000 3.323800
N 2.881100 2.981100 0.720200
N 3.324500 2.669500 -1.359400
C 3.029000 2.010900 -0.212600
C 3.362100 4.028100 -1.145400
H 3.574100 4.730200 -1.936800
C 3.088000 4.224100 0.167600
H 3.018700 5.128200 0.751100
C 2.523200 2.764000 2.106200
H 2.104300 1.759800 2.201500
H 3.401400 2.855100 2.753200
C 3.461200 2.032300 -2.651500
H 4.025200 1.102700 -2.538800
H 2.473900 1.817500 -3.071500
H 1.764700 3.499600 2.384300
H 4.006000 2.698100 -3.323600
O 0.065100 4.778300 1.080400
H 0.039800 4.234400 0.268700
C 0.044800 6.133300 0.694400
H -0.867100 6.396800 0.138600
H 0.077600 6.737700 1.605500
H 0.909000 6.403900 0.070100
O 0.053200 -4.778800 -1.080300
H 0.029100 -4.235100 -0.268500
C 0.030500 -6.133900 -0.694700
H 0.061600 -6.738100 -1.606100
H 0.894500 -6.406400 -0.071000
H -0.881600 -6.395800 -0.138500

I-3S1

85
Molecule Name
Au -0.119800 2.843900 0.090800
N 2.772700 2.922400 1.108900
N 2.689100 3.251200 -1.014700
C 1.909400 3.024000 0.070000
C 4.017000 3.292700 -0.657400
H 4.801800 3.463100 -1.378300
C 4.069300 3.089300 0.682300
H 4.905300 3.052300 1.363000
C 2.404500 2.625800 2.478300
H 1.385800 2.231200 2.482200
H 3.086600 1.863400 2.863500
C 2.208000 3.316700 -2.381700
H 1.174000 3.669200 -2.372600

H	2.816700	4.029700	-2.943200
H	2.448700	3.527900	3.096300
H	2.262100	2.328900	-2.852900
N	-3.009800	2.794300	-0.937600
N	-2.922500	3.425000	1.115000
C	-2.145400	3.033000	0.076400
C	-4.250500	3.432400	0.753800
H	-5.033500	3.709300	1.442600
C	-4.304300	3.039400	-0.542500
H	-5.140300	2.906700	-1.210200
C	-2.644200	2.318200	-2.255800
H	-1.657100	1.854100	-2.195600
H	-3.377500	1.569700	-2.565100
C	-2.428100	3.694300	2.448400
H	-1.482100	4.237500	2.380400
H	-3.153400	4.311800	2.981800
H	-2.614700	3.144400	-2.973100
H	-2.276700	2.757600	2.993400
N	-3.215500	-0.000500	0.980600
C	-2.066800	-0.000300	0.756400
N	2.923400	0.000300	-0.503500
C	1.783000	0.000100	-0.231500
Au	-0.140500	-0.000200	0.261800
Au	-0.119100	-2.844200	0.090600
N	2.773100	-2.921300	1.109700
N	2.690400	-3.251000	-1.013800
C	1.910200	-3.023700	0.070500
C	4.018200	-3.291900	-0.656000
H	4.803200	-3.462200	-1.376600
C	4.069900	-3.088000	0.683700
H	4.905700	-3.050600	1.364600
C	2.404200	-2.624500	2.478900
H	1.385900	-2.228900	2.482000
H	2.447300	-3.526600	3.096900
C	2.209700	-3.317400	-2.380900
H	1.176300	-3.671700	-2.372100
H	2.262200	-2.329500	-2.852200
H	3.086700	-1.862600	2.864600
H	2.819900	-4.029200	-2.942200
N	-3.008900	-2.794300	-0.938600
N	-2.922000	-3.426100	1.113800
C	-2.144700	-3.033500	0.075500
C	-4.249800	-3.433300	0.752300
H	-5.033000	-3.710500	1.440900
C	-4.303500	-3.039500	-0.543700
H	-5.139400	-2.906500	-1.211600
C	-2.643000	-2.317900	-2.256500
H	-1.655900	-1.853900	-2.196000
H	-2.613400	-3.143900	-2.974100
C	-2.427800	-3.695900	2.447100
H	-1.482200	-4.239800	2.379100
H	-2.275700	-2.759400	2.992200
H	-3.376200	-1.569200	-2.565800
H	-3.153600	-4.313000	2.980400
O	-4.885600	0.000100	-1.393500
H	-4.409900	0.000100	-0.538500
C	-6.268000	-0.000100	-1.120900
H	-6.583600	0.888600	-0.554600
H	-6.583200	-0.888600	-0.554200
H	-6.795200	-0.000400	-2.079200
O	4.493500	0.000700	1.959400
H	4.071200	0.000400	1.079300
C	5.890400	0.000800	1.777500
H	6.244000	-0.888000	1.234000
H	6.243900	0.889400	1.233500
H	6.353000	0.001100	2.768400
C	4.159100	0.001700	-4.046900
O	2.908000	0.001400	-3.398000
H	4.758100	-0.889600	-3.806500
H	4.758700	0.892100	-3.804800
H	3.061500	0.001300	-2.435200
H	3.970900	0.002800	-5.123700

I-4S1

91

Molecule Name

Au	0.000000	2.853600	0.000000
N	2.853600	3.106000	1.075200
N	2.853600	3.106000	-1.075200
C	2.031800	3.036300	0.000000
C	4.165300	3.222200	-0.677800
H	4.975500	3.297400	-1.386700
C	4.165300	3.222200	0.677800
H	4.975500	3.297400	1.386700
C	2.441800	2.985800	2.460400
H	1.356700	2.876300	2.489000
H	2.909900	2.099700	2.901400
C	2.441800	2.985800	-2.460400
H	1.356700	2.876300	-2.489000
H	2.724900	3.884100	-3.016800
H	2.724900	3.884100	3.016800
H	2.909900	2.099700	-2.901400
N	-2.853600	3.106000	-1.075200
N	-2.853600	3.106000	1.075200
C	-2.031800	3.036300	0.000000
C	-4.165300	3.222200	0.677800
H	-4.975500	3.297400	1.386700
C	-4.165300	3.222200	-0.677800
H	-4.975500	3.297400	-1.386700
C	-2.441800	2.985800	-2.460400
H	-1.356700	2.876300	-2.489000
H	-2.909900	2.099700	-2.901400
C	-2.441800	2.985800	2.460400
H	-1.356700	2.876300	2.489000
H	-2.724900	3.884100	3.016800
H	-2.724900	3.884100	-3.016800
H	-2.909900	2.099700	2.901400
N	-3.153900	0.000000	0.000000
C	-1.981900	0.000000	0.000000
N	3.153900	0.000000	0.000000
C	1.981900	0.000000	0.000000
Au	0.000000	0.000000	0.000000
Au	0.000000	-2.853600	0.000000
N	2.853600	-3.106000	1.075200
N	2.853600	-3.106000	-1.075200
C	2.031800	-3.036300	0.000000
C	4.165300	-3.222200	-0.677800
H	4.975500	-3.297400	-1.386700
C	4.165300	-3.222200	0.677800
H	4.975500	-3.297400	1.386700
C	2.441800	-2.985800	2.460400
H	1.356700	-2.876300	2.489000
H	2.724900	-3.884100	3.016800
C	2.441800	-2.985800	-2.460400
H	1.356700	-2.876300	-2.489000
H	2.909900	-2.099700	-2.901400
H	2.909900	-2.099700	2.901400

H	2.724900	-3.884100	-3.016800
N	-2.853600	-3.106000	-1.075200
N	-2.853600	-3.106000	1.075200
C	-2.031800	-3.036300	0.000000
C	-4.165300	-3.222200	0.677800
H	-4.975500	-3.297400	1.386700
C	-4.165300	-3.222200	-0.677800
H	-4.975500	-3.297400	-1.386700
C	-2.441800	-2.985800	-2.460400
H	-1.356700	-2.876300	-2.489000
H	-2.724900	-3.884100	-3.016800
C	-2.441800	-2.985800	2.460400
H	-1.356700	-2.876300	2.489000
H	-2.909900	-2.099700	2.901400
H	-2.909900	-2.099700	-2.901400
H	-2.724900	-3.884100	3.016800
O	-4.108600	0.000000	-2.725700
H	-3.906700	0.000000	-1.770600
C	-5.508600	0.000000	-2.881000
H	-5.981400	0.889800	-2.438300
H	-5.981400	-0.889800	-2.438300
H	-5.721800	0.000000	-3.953200
O	4.108600	0.000000	2.725700
H	3.906700	0.000000	1.770600
C	5.508600	0.000000	2.881000
H	5.981400	-0.889800	2.438300
H	5.981400	0.889800	2.438300
H	5.721800	0.000000	3.953200
C	5.508600	0.000000	-2.881000
O	4.108600	0.000000	-2.725700
H	5.981400	-0.889800	-2.438300
H	5.981400	0.889800	-2.438300
H	3.906700	0.000000	-1.770600
H	5.721800	0.000000	-3.953200
C	-5.508600	0.000000	2.881000
O	-4.108600	0.000000	2.725700
H	-5.981400	-0.889800	2.438300
H	-5.981400	0.889800	2.438300
H	-3.906700	0.000000	1.770600
H	-5.721800	0.000000	3.953200

20. The T1 .xyz files of II and its complexes

II

91
Molecule Name
Au 0.000000 2.768000 -0.000600
N 2.983000 2.610500 0.611500
N 2.630300 3.009100 -1.499400
C 2.006500 2.826800 -0.308700
C 4.230500 2.668500 0.013900
C 5.517900 2.540400 0.527200
H 5.699100 2.365300 1.582700
C 6.567900 2.653000 -0.377900
H 7.588400 2.560700 -0.019200
C 6.339700 2.887100 -1.741900
H 7.187500 2.968600 -2.415000
C 5.053100 3.020300 -2.253200
H 4.881600 3.198400 -3.309800
C 4.002500 2.911300 -1.346700
C 1.963200 3.241600 -2.764300
H 0.960500 3.624000 -2.562800
H 1.884200 2.312600 -3.335900
H 2.518300 3.994000 -3.330700
C 2.767200 2.391300 2.024600
H 1.724000 2.115200 2.188900
H 3.004400 3.293700 2.597700
H 3.401900 1.568800 2.366200
N -2.983100 2.607700 -0.611900
N -2.630000 3.008400 1.498500
C -2.006500 2.825700 0.307800
C -4.230400 2.665000 -0.013900
C -5.517900 2.535200 -0.526600
H -5.699300 2.359100 -1.582000
C -6.567700 2.647500 0.378700
H -7.588200 2.553900 0.020500
C -6.339200 2.882900 1.742400
H -7.186800 2.964000 2.415800
C -5.052600 3.017800 2.253200
H -4.880900 3.196900 3.309500
C -4.002100 2.909100 1.346400
C -1.962400 3.242400 2.762900
H -0.961300 3.628600 2.560600
H -2.519700 3.992600 3.330300
H -1.879700 2.313400 3.333900
C -2.767700 2.387600 -2.025000
H -1.724100 2.113000 -2.189500
H -3.401200 1.563800 -2.365500
H -3.006600 3.289100 -2.598700
N -0.661900 0.016500 3.093200
C -0.412900 0.012800 1.951100
N 0.663200 0.015600 -3.092700
C 0.413600 0.012200 -1.950800
Au -0.000300 -2.750700 0.001500
N 2.608400 -2.756900 1.576500
N 2.982000 -2.851900 -0.567200
C 1.994400 -2.813700 0.364900
C 3.984000 -2.779800 1.422200
C 5.027600 -2.772500 2.343500
H 4.847300 -2.758100 3.413600
C 6.320200 -2.796100 1.829600
H 7.162300 -2.794600 2.514800
C 6.560500 -2.829100 0.448500
H 7.584400 -2.847400 0.088300
C 5.517600 -2.844200 -0.472000
H 5.708900 -2.869100 -1.539800
C 4.224300 -2.824800 0.042300
C 2.771100 -2.881600 -1.999300
H 1.774900 -3.280000 -2.199300
H 3.511500 -3.541400 -2.458700
H 2.843500 -1.878600 -2.429100
C 1.928000 -2.755300 2.854200
H 0.933400 -2.323200 2.734000
H 2.483700 -2.130800 3.557700
H 1.851000 -3.771700 3.254300
N -2.607200 -2.757100 -1.575600
N -2.984100 -2.845700 0.567700
C -1.995000 -2.811300 -0.363000
C -3.983100 -2.778200 -1.423400
C -5.025400 -2.772200 -2.346100
H -4.843700 -2.760700 -3.416000
C -6.318700 -2.793400 -1.833800
H -7.159900 -2.792900 -2.520200
C -6.560900 -2.822700 -0.452900
H -7.585400 -2.839300 -0.094200
C -5.519300 -2.836500 0.468900
H -5.711900 -2.858700 1.536600
C -4.225400 -2.819400 -0.043700
C -2.776600 -2.872100 2.000300
H -1.774200 -3.253000 2.203000
H -2.867000 -1.870300 2.429600
H -3.507100 -3.544200 2.458200
C -1.924800 -2.759100 -2.852300
H -0.930400 -2.326700 -2.731800
H -1.847200 -3.776700 -3.249400
H -2.479400 -2.136700 -3.558400
Au 0.000200 0.004100 0.000200

II-S1

97
Molecule Name
Au -0.104200 2.772000 -0.119700
N -2.998600 2.921800 -1.036900
N -2.873400 2.957300 1.136600
C -2.125300 2.895200 0.003900
C -4.217700 3.064800 0.823300
C -2.624800 2.887900 -2.435000
H -1.673800 2.363200 -2.542700
H -3.381700 2.334700 -2.995700
C -2.344900 2.968600 2.483700
H -1.369500 2.479800 2.491100
H -2.253600 3.993800 2.857500
N -0.171500 0.016400 -3.280400
C -0.162100 0.014000 -2.111400
H -2.542500 3.901900 -2.840300
H -3.012900 2.402400 3.137200
N 2.822600 2.726400 0.743500
N 2.636100 2.818000 -1.424400
C 1.921900 2.802900 -0.268400
C 3.991900 2.748700 -1.154500
C 2.507000 2.732600 2.157400
H 1.422400 2.757300 2.268600
H 2.888100 1.830700 2.648300
C 2.067300 2.885300 -2.753900

H	1.058900	3.297100	-2.683400
H	2.675400	3.550400	-3.372700
N	0.039100	-0.008700	3.036300
C	-0.056100	-0.003200	1.870800
H	2.935700	3.624300	2.626600
H	2.011400	1.893300	-3.210500
Au	-0.068300	-2.756000	-0.151300
N	-2.932300	-2.956400	-1.159700
N	-2.869900	-2.985100	1.016500
C	-2.090300	-2.911700	-0.094200
C	-4.202300	-3.118300	0.664700
C	-2.519800	-2.920200	-2.546900
H	-1.579400	-2.373100	-2.630900
H	-2.402900	-3.933800	-2.944500
C	-2.381600	-2.981100	2.378700
H	-1.418200	-2.469900	2.414400
H	-3.082100	-2.429200	3.010100
H	-3.273300	-2.387000	-3.131200
H	-2.278100	-4.002900	2.758600
N	2.814400	-2.761200	0.838100
N	2.732900	-2.712600	-1.337200
C	1.963300	-2.766900	-0.218400
C	4.074200	-2.678900	-0.998500
C	2.433600	-2.839700	2.233900
H	1.344800	-2.847900	2.296300
H	2.825000	-3.764100	2.671600
C	2.240000	-2.686500	-2.697000
H	1.168000	-2.884600	-2.687700
H	2.406200	-1.705400	-3.150400
H	2.809700	-1.971200	2.785700
H	2.743000	-3.459500	-3.285500
Au	-0.126400	0.007400	-0.120700
C	-4.242600	-3.100000	-0.735600
C	-5.442700	-3.217800	-1.430900
C	-6.599700	-3.350000	-0.670300
H	-5.479500	-3.217100	-2.515500
C	-5.359500	-3.255000	1.426000
C	-6.559100	-3.368200	0.731300
H	-7.555900	-3.449200	-1.174600
H	-5.332200	-3.282200	2.510500
H	-7.484500	-3.481000	1.287400
C	4.126000	-2.711900	0.400300
C	5.336500	-2.704100	1.086900
C	6.492900	-2.660200	0.315800
H	5.377500	-2.727100	2.171000
C	5.231200	-2.634500	-1.771100
C	6.441400	-2.626300	-1.085900
H	7.459200	-2.653500	0.810400
H	5.195200	-2.608300	-2.855500
H	7.368700	-2.594400	-1.649600
C	-4.298400	3.042200	-0.575200
C	-5.520300	3.136200	-1.235400
C	-5.354600	3.182100	1.617700
C	-6.656900	3.249000	-0.441600
H	-5.588600	3.131900	-2.318400
C	-6.575900	3.271600	0.958100
H	-5.296000	3.212700	2.700900
H	-7.629100	3.329200	-0.918000
H	-7.486600	3.368900	1.540800
C	4.110700	2.694800	0.240100
C	5.352200	2.636600	0.866500
C	5.111600	2.740900	-1.981500
C	6.470800	2.630000	0.040500
H	5.444000	2.594200	1.946900
C	6.352700	2.681400	-1.356400
H	5.026200	2.778000	-3.062800
H	7.459100	2.585000	0.487400
H	7.251700	2.675300	-1.965000
H	1.805600	-0.027400	3.831000
O	2.769200	-0.041200	3.988500
C	2.991200	0.013100	5.381400
H	2.594800	0.934500	5.831100
H	2.550900	-0.845700	5.907700
H	4.072600	-0.006500	5.541800

II-2S1

103
Molecule Name

Au	0.000100	-2.858500	-0.000100
N	2.922000	-3.061700	-0.859100
N	2.713400	-3.307200	1.290500
C	2.017100	-3.071000	0.149000
C	4.060300	-3.454900	1.014800
C	2.643300	-2.758300	-2.246300
H	1.587400	-2.497300	-2.337900
H	3.256400	-1.903800	-2.554200
C	2.151400	-3.361700	2.619200
H	1.063000	-3.363100	2.539300
H	2.470300	-4.279000	3.123200
H	2.856800	-3.628000	-2.876300
H	2.470900	-2.492700	3.203100
N	-2.921900	-3.061300	0.858900
N	-2.713100	-3.307300	-1.290700
C	-2.016900	-3.070900	-0.149100
C	-4.060100	-3.454800	-1.015000
C	-2.643100	-2.757600	2.246000
H	-1.587000	-2.497500	2.337800
H	-3.255700	-1.902700	2.553600
C	-2.151100	-3.362400	-2.619300
H	-1.062700	-3.361800	-2.539500
H	-2.468500	-4.280800	-3.122300
H	-2.857400	-3.627100	2.876200
H	-2.472100	-2.494500	-3.204200
C	4.192600	-3.303500	-0.370200
C	5.425100	-3.431200	-1.003300
C	5.156300	-3.724900	1.828200
C	6.521800	-3.703400	-0.191800
H	5.527400	-3.322900	-2.078100
C	6.390300	-3.844800	1.197700
H	5.058700	-3.837500	2.903200
H	7.502300	-3.813100	-0.645000
H	7.271700	-4.057000	1.794800
C	-4.192500	-3.303000	0.370000
C	-5.425000	-3.430200	1.003000
C	-5.156100	-3.724800	-1.828400
C	-6.521700	-3.702300	0.191500
H	-5.527400	-3.321600	2.077700
C	-6.390100	-3.844200	-1.198000
H	-5.058400	-3.837800	-2.903300
H	-7.502300	-3.811600	0.644600
H	-7.271600	-4.056400	-1.795100
N	-3.137800	-0.000100	-0.376300
C	-1.972700	-0.000100	-0.273200
N	3.137500	0.000000	0.377500
C	1.972500	0.000000	0.273500
Au	-0.000100	0.000000	-0.000000
Au	-0.000000	2.858500	-0.000100
N	2.922000	3.061800	-0.858800
N	2.713100	3.307200	1.290800

C	2.017000	3.071000	0.149100
C	4.060100	3.454900	1.015100
C	2.643300	2.758700	-2.246200
H	1.587500	2.497500	-2.337800
H	2.856500	3.628800	-2.875800
C	2.151000	3.361500	2.619400
H	1.062600	3.363000	2.539400
H	2.470500	2.492500	3.203300
H	3.256700	1.904600	-2.554400
H	2.469900	4.278900	3.123400
N	-2.922100	3.061300	0.858600
N	-2.713200	3.307100	-1.291000
C	-2.017000	3.070900	-0.149300
C	-4.060200	3.454600	-1.015300
C	-2.643400	2.757900	2.245900
H	-1.587300	2.497900	2.337700
H	-2.857900	3.627400	2.875900
C	-2.151100	3.361900	-2.619500
H	-1.062700	3.361500	-2.539600
H	-2.472000	2.494000	-3.204300
H	-3.255900	1.902900	2.553500
H	-2.468500	4.280200	-3.122700
C	4.192500	3.303600	-0.369800
C	5.425000	3.431300	-1.002800
C	6.521600	3.703500	-0.191200
H	5.527500	3.323100	-2.077500
C	5.156000	3.724900	1.828700
C	6.390000	3.844800	1.198300
H	7.502200	3.813300	-0.644300
H	5.058300	3.837400	2.903700
H	7.271400	4.057100	1.795500
C	-4.192700	3.302900	0.369600
C	-5.425200	3.430200	1.002500
C	-6.521900	3.702200	0.190900
H	-5.527700	3.321800	2.077200
C	-5.156100	3.724400	-1.828900
C	-6.390200	3.843900	-1.198600
H	-7.502500	3.811500	0.643900
H	-5.058300	3.837300	-2.903800
H	-7.271600	4.056000	-1.795800
O	-4.566800	-0.000200	2.123800
H	-4.261000	-0.000300	1.195400
C	-5.978400	-0.000000	2.130900
H	-6.396200	-0.890000	1.640700
H	-6.299600	0.000300	3.176800
H	-6.396100	0.889800	1.640200
O	4.566700	0.000300	-2.122300
H	4.259900	0.000200	-1.194200
C	5.978300	0.000200	-2.127800
H	6.300700	0.000200	-3.173300
H	6.395600	0.890000	-1.636800
H	6.395400	-0.889800	-1.636800

II-3S1

109
Molecule Name

Au	-0.074100	0.123000	2.853100
N	-1.038300	-2.758200	3.111100
N	1.133900	-2.652500	3.147800
C	0.010400	-1.901400	3.032700
C	0.807800	-3.985600	3.311500
C	-2.432000	-2.409300	2.938300
H	-2.498800	-1.336700	2.748300
H	-2.836600	-2.953600	2.077500
C	2.492900	-2.168700	3.029000
H	2.468700	-1.080000	2.957500
H	3.069400	-2.452600	3.914900
H	-2.999000	-2.650800	3.843100
H	2.951900	-2.586900	2.125900
N	0.908100	3.003900	3.057200
N	-1.241900	2.885200	3.353500
C	-0.137100	2.142900	3.085500
C	-0.905100	4.218100	3.503400
C	2.275500	2.668700	2.721100
H	2.315700	1.613800	2.444300
H	2.592400	3.280100	1.868800
C	-2.592500	2.381600	3.433500
H	-2.562000	1.291300	3.404000
H	-3.055800	2.698000	4.373000
H	2.931400	2.842000	3.580400
H	-3.185200	2.749700	2.590100
C	-0.590200	-4.053400	3.289800
C	-1.264800	-5.258900	3.459300
C	1.591100	-5.120500	3.500600
C	-0.483000	-6.395000	3.644000
H	-2.348700	-5.312100	3.452700
C	0.918200	-6.327200	3.664100
H	2.674800	-5.068400	3.524100
H	-0.969000	-7.355700	3.784100
H	1.490600	-7.236800	3.818300
C	0.480000	4.292600	3.318300
C	1.167500	5.496600	3.436200
C	-1.664800	5.345400	3.799700
C	0.409900	6.624500	3.734300
H	2.242500	5.554400	3.300700
C	-0.980100	6.550800	3.910300
H	-2.739800	5.292300	3.939000
H	0.906700	7.584400	3.837500
H	-1.533800	7.455300	4.142100
N	-0.347600	3.298900	0.000000
C	-0.318700	2.129900	0.000000
N	0.061200	-3.005700	0.000000
C	-0.022200	-1.837500	0.000000
Au	-0.176000	0.145500	0.000000
Au	-0.074100	0.123000	-2.853100
N	-1.038300	-2.758200	-3.111100
N	1.133900	-2.652500	-3.147800
C	0.010400	-1.901400	-3.032700
C	0.807800	-3.985600	-3.311500
C	-2.432000	-2.409300	-2.938300
H	-2.498800	-1.336700	-2.748300
H	-2.999000	-2.650800	-3.843100
C	2.492900	-2.168700	-3.029000
H	2.468700	-1.080000	-2.957500
H	2.951900	-2.586900	-2.125900
H	-2.836600	-2.953600	-2.077500
H	3.069400	-2.452600	-3.914900
N	0.908100	3.003900	-3.057200
N	-1.241900	2.885200	-3.353500
C	-0.137100	2.142900	-3.085500
C	-0.905100	4.218100	-3.503400
C	2.275500	2.668700	-2.721100
H	2.315700	1.613800	-2.444300
H	2.931400	2.842000	-3.580400
C	-2.592500	2.381600	-3.433500
H	-2.562000	1.291300	-3.404000
H	-3.185200	2.749700	-2.590100
H	2.592400	3.280100	-1.868800
H	-3.055800	2.698000	-4.373000

C	-0.590200	-4.053400	-3.289800
C	-1.264800	-5.258900	-3.459300
C	-0.483000	-6.395000	-3.644000
H	-2.348700	-5.312100	-3.452700
C	1.591100	-5.120500	-3.500600
C	0.918200	-6.327200	-3.664100
H	-0.969000	-7.355700	-3.784100
H	2.674800	-5.068400	-3.524100
H	1.490600	-7.236800	-3.818300
C	0.480000	4.292600	-3.318300
C	1.167500	5.496600	-3.436200
C	0.409900	6.624500	-3.734300
H	2.242500	5.554400	-3.300700
C	-1.664800	5.345400	-3.799700
C	-0.980100	6.550800	-3.910300
H	0.906700	7.584400	-3.837500
H	-2.739800	5.292300	-3.939000
H	-1.533800	7.455300	-4.142100
O	2.202600	4.637800	0.000000
H	1.263300	4.367800	0.000000
C	2.263800	6.048000	0.000000
H	1.789900	6.484200	0.890100
H	3.321300	6.328700	0.000000
H	1.789900	6.484200	-0.890100
O	-2.629600	-4.142900	0.000000
H	-1.676800	-3.935200	0.000000
C	-2.778100	-5.546900	0.000000
H	-3.850600	-5.761000	0.000000
H	-2.332000	-6.012100	-0.890200
H	-2.332000	-6.012100	0.890200
H	1.922000	-3.635300	0.000000
O	2.894100	-3.713100	0.000000
C	3.226800	-5.085000	0.000000
H	4.318000	-5.156100	0.000000
H	2.845800	-5.605500	0.890100
H	2.845800	-5.605500	-0.890100

II-4S1

115

Molecule Name

Au	0.000000	2.848600	0.000000
N	2.830000	3.135000	1.087500
N	2.830000	3.135000	-1.087500
C	2.025400	3.038800	0.000000
C	4.146000	3.302800	-0.699900
C	2.413100	2.992300	2.466100
H	1.328700	2.873900	2.489900
H	2.886800	2.104200	2.899700
C	2.413100	2.992300	-2.466100
H	1.328700	2.873900	-2.489900
H	2.686500	3.886000	-3.036000
H	2.686500	3.886000	3.036000
H	2.886800	2.104200	-2.899700
N	-2.830000	3.135000	-1.087500
N	-2.830000	3.135000	1.087500
C	-2.025400	3.038800	0.000000
C	-4.146000	3.302800	0.699900
C	-2.413100	2.992300	-2.466100
H	-1.328700	2.873900	-2.489900
H	-2.886800	2.104200	-2.899700
C	-2.413100	2.992300	2.466100
H	-1.328700	2.873900	2.489900
H	-2.686500	3.886000	3.036000
H	-2.686500	3.886000	-3.036000
H	-2.886800	2.104200	2.899700
C	4.146000	3.302800	0.699900
C	5.318100	3.477600	1.429900
C	5.318100	3.477600	-1.429900
C	6.491300	3.647100	0.701600
H	5.318700	3.485500	2.515100
C	6.491300	3.647100	-0.701600
H	5.318700	3.485500	-2.515100
H	7.428000	3.790500	1.231300
H	7.428000	3.790500	-1.231300
C	-4.146000	3.302800	-0.699900
C	-5.318100	3.477600	-1.429900
C	-5.318100	3.477600	1.429900
C	-6.491300	3.647100	-0.701600
H	-5.318700	3.485500	-2.515100
C	-6.491300	3.647100	0.701600
H	-5.318700	3.485500	2.515100
H	-7.428000	3.790500	-1.231300
H	-7.428000	3.790500	1.231300
N	-3.158200	0.000000	0.000000
C	-1.987200	0.000000	0.000000
N	3.158200	0.000000	0.000000
C	1.987200	0.000000	0.000000
Au	0.000000	0.000000	0.000000
Au	0.000000	-2.848600	0.000000
N	2.830000	-3.135000	1.087500
N	2.830000	-3.135000	-1.087500
C	2.025400	-3.038800	0.000000
C	4.146000	-3.302800	-0.699900
C	2.413100	-2.992300	2.466100
H	1.328700	-2.873900	2.489900
H	2.686500	-3.886000	3.036000
C	2.413100	-2.992300	-2.466100
H	1.328700	-2.873900	-2.489900
H	2.886800	-2.104200	-2.899700
H	2.886800	-2.104200	2.899700
H	2.686500	-3.886000	-3.036000
N	-2.830000	-3.135000	-1.087500
N	-2.830000	-3.135000	1.087500
C	-2.025400	-3.038800	0.000000
C	-4.146000	-3.302800	0.699900
C	-2.413100	-2.992300	-2.466100
H	-1.328700	-2.873900	-2.489900
H	-2.686500	-3.886000	-3.036000
C	-2.413100	-2.992300	2.466100
H	-1.328700	-2.873900	2.489900
H	-2.886800	-2.104200	2.899700
H	-2.886800	-2.104200	-2.899700
H	-2.686500	-3.886000	3.036000
C	4.146000	-3.302800	0.699900
C	5.318100	-3.477600	1.429900
C	6.491300	-3.647100	0.701600
H	5.318700	-3.485500	2.515100
C	5.318100	-3.477600	-1.429900
C	6.491300	-3.647100	-0.701600
H	7.428000	-3.790500	1.231300
H	5.318700	-3.485500	-2.515100
H	7.428000	-3.790500	-1.231300
C	-4.146000	-3.302800	-0.699900
C	-5.318100	-3.477600	-1.429900
C	-6.491300	-3.647100	-0.701600
H	-5.318700	-3.485500	-2.515100
C	-5.318100	-3.477600	1.429900
C	-6.491300	-3.647100	0.701600
H	-7.428000	-3.790500	-1.231300

H	-5.318700	-3.485500	2.515100
H	-7.428000	-3.790500	1.231300
O	-4.053400	0.000000	-2.785000
H	-3.926900	0.000000	-1.818500
C	-5.440000	0.000000	-3.050200
H	-5.941100	0.890100	-2.644200
H	-5.564000	0.000000	-4.136700
H	-5.941100	-0.890100	-2.644200
O	4.053400	0.000000	2.785000
H	3.926900	0.000000	1.818500
C	5.440000	0.000000	3.050200
H	5.564000	0.000000	4.136700
H	5.941100	-0.890100	2.644200
H	5.941100	0.890100	2.644200
H	3.926900	0.000000	-1.818500
O	4.053400	0.000000	-2.785000
C	5.440000	0.000000	-3.050200
H	5.564000	0.000000	-4.136700
H	5.941100	0.890100	-2.644200
H	5.941100	-0.890100	-2.644200
H	-3.926900	0.000000	1.818500
O	-4.053400	0.000000	2.785000
C	-5.440000	0.000000	3.050200
H	-5.941100	-0.890100	2.644200
H	-5.564000	0.000000	4.136700
H	-5.941100	0.890100	2.644200

II-S2

100

Molecule Name

Au	-0.388500	2.739900	-0.446600
N	-3.430000	2.658400	-0.635100
N	-2.777400	3.199400	1.370400
C	-2.329300	2.905600	0.122500
C	-4.159800	3.141800	1.414900
C	-3.415000	2.328100	-2.043600
H	-2.431100	1.937800	-2.310100
H	-4.157500	1.550700	-2.241700
C	-1.930500	3.498200	2.506100
H	-0.973400	3.873600	2.137900
H	-2.401500	4.276500	3.111700
N	-0.626200	-0.363900	-3.298600
C	-0.519300	-0.230200	-2.142200
H	-3.641900	3.209900	-2.651800
H	-1.755600	2.603900	3.111100
N	2.643700	2.475600	-0.229200
N	2.000600	2.568400	-2.309300
C	1.556200	2.640500	-1.027200
C	3.366000	2.347200	-2.334600
C	2.643500	2.536900	1.218200
H	1.614100	2.638300	1.562800
H	3.062300	1.627300	1.657300
C	1.169500	2.695400	-3.489000
H	0.250200	3.215600	-3.212300
H	1.697300	3.292800	-4.237300
N	-0.007200	0.360800	2.952800
C	-0.151100	0.213800	1.801900
H	3.222500	3.403900	1.553200
H	0.914900	1.713200	-3.896700
Au	0.150600	-2.722300	0.005900
N	-2.482300	-3.488200	-1.295300
N	-2.769200	-3.075600	0.823100
C	-1.828700	-3.149300	-0.154900
C	-4.020000	-3.380200	0.313900
C	-1.860200	-3.655100	-2.593100
H	-0.801100	-3.876500	-2.445800
H	-2.326100	-4.498900	-3.108600
C	-2.508600	-2.768100	2.212200
H	-1.540000	-2.272200	2.292800
H	-3.276700	-2.084400	2.583400
H	-1.953700	-2.745300	-3.192400
H	-2.510100	-3.679900	2.818300
N	2.875000	-2.364100	1.308500
N	3.028500	-2.190000	-0.856000
C	2.156400	-2.424900	0.160500
C	4.307700	-2.005000	-0.360000
C	2.358400	-2.568100	2.647100
H	1.278100	-2.705700	2.584700
H	2.810100	-3.464300	3.085200
C	2.699400	-2.182500	-2.265000
H	1.619200	-2.088200	-2.383300
H	3.175200	-1.322700	-2.745000
H	2.577200	-1.687500	3.260000
H	3.045000	-3.103700	-2.745900
Au	-0.331900	-0.007900	-0.171400
C	-3.837400	-3.632100	-1.052100
C	-4.906100	-3.962100	-1.880700
C	-6.161200	-4.039600	-1.286100
H	-4.772200	-4.148800	-2.941200
C	-5.274700	-3.463800	0.910700
C	-6.342100	-3.796600	0.083400
H	-7.020900	-4.296900	-1.897000
H	-5.418100	-3.283300	1.971200
H	-7.337900	-3.873300	0.508600
C	4.205700	-2.106100	1.032600
C	5.315000	-1.959200	1.860200
C	6.533600	-1.717700	1.235300
H	5.232200	-2.023100	2.939900
C	5.527300	-1.765900	-0.986600
C	6.637800	-1.625500	-0.161000
H	7.426300	-1.602000	1.842300
H	5.612500	-1.690800	-2.065700
H	7.610100	-1.443900	-0.608900
C	-4.580300	2.807600	0.120800
C	-5.929500	2.689000	-0.200100
C	-5.071800	3.354700	2.444700
C	-6.841000	2.904900	0.828200
H	-6.259900	2.444700	-1.204500
C	-6.420000	3.230200	2.125800
H	-4.752200	3.604900	3.451100
H	-7.903200	2.823100	0.620100
H	-7.163800	3.391300	2.899900
C	3.780400	2.295900	-0.996700
C	5.114600	2.102400	-0.651700
C	4.269400	2.197100	-3.383300
C	6.017700	1.954800	-1.699500
H	5.436100	2.057800	0.383700
C	5.603600	2.001200	-3.038800
H	3.953500	2.232200	-4.421000
H	7.068400	1.801100	-1.474000
H	6.341600	1.886100	-3.827000
H	1.784200	0.486100	3.703000
O	2.751400	0.458700	3.833400
C	3.041500	0.881700	5.154100
H	2.708500	1.919400	5.311600
H	2.512000	0.256300	5.889200
C	4.535100	0.783200	5.373200
H	4.802200	1.117800	6.380700
H	4.875000	-0.251300	5.254000

H 5.071400 1.405100 4.648700

II-2S2

109
Molecule Name
Au -0.000100 -2.861000 -0.000200
N -2.969600 -3.035100 0.684100
N -2.627600 -3.361400 -1.437600
C -2.004100 -3.078500 -0.264800
C -3.988700 -3.505900 -1.240000
C -2.775700 -2.679000 2.073200
H -1.743500 -2.348400 2.203900
H -3.452100 -1.854900 2.325500
C -1.982900 -3.468100 -2.725100
H -0.902400 -3.503900 -2.574600
H -2.299500 -4.388500 -3.224700
H -2.966900 -3.541700 2.720000
H -2.234700 -2.606300 -3.351400
N 2.969500 -3.035100 -0.684400
N 2.627300 -3.362600 1.437100
C 2.003900 -3.078900 0.264400
C 3.988300 -3.507300 1.239400
C 2.775700 -2.677900 -2.073300
H 1.743800 -2.346400 -2.203700
H 3.452700 -1.854200 -2.325100
C 1.982400 -3.469900 2.724500
H 0.902000 -3.506400 2.573800
H 2.299700 -4.390000 3.224000
H 2.966200 -3.540400 -2.720700
H 2.233500 -2.607900 3.350800
C -4.206700 -3.303400 0.127600
C -5.475100 -3.415100 0.688400
C -5.031700 -3.812300 -2.108500
C -6.518600 -3.724400 -0.177800
H -5.643300 -3.266300 1.749900
C -6.301400 -3.916600 -1.550500
H -4.868300 -3.963800 -3.170600
H -7.524700 -3.823000 0.218200
H -7.143700 -4.156200 -2.192000
C 4.206400 -3.304000 -0.128000
C 5.474800 -3.415900 -0.688900
C 5.031300 -3.814500 2.107800
C 6.518200 -3.726100 0.177100
H 5.643000 -3.266700 -1.750400
C 6.300900 -3.919000 1.549700
H 4.867800 -3.966600 3.169800
H 7.524300 -3.825000 -0.218900
H 7.143100 -4.159300 2.191100
N 3.109600 -0.000100 0.564100
C 1.953000 -0.000000 0.392100
N -3.109900 0.000100 -0.563000
C -1.953200 0.000000 -0.391300
Au -0.000100 0.000000 0.000300
Au 0.000000 2.861100 -0.000100
N -2.970000 3.036200 0.681600
N -2.626200 3.360400 -1.440200
C -2.003700 3.078600 -0.266600
C -3.987500 3.505200 -1.243800
C -2.777400 2.681300 2.071200
H -1.745000 2.351800 2.203300
H -2.970000 3.544500 2.717000
C -1.980400 3.465800 -2.727200
H -0.900100 3.501600 -2.575900
H -2.231900 2.603500 -3.352900
H -3.453300 1.856900 2.323600
H -2.296500 4.385800 -3.227900
N 2.970100 3.035900 -0.681800
N 2.626300 3.361300 1.439700
C 2.003800 3.078800 0.266300
C 3.987500 3.506100 1.243200
C 2.777400 2.680300 -2.071200
H 1.745400 2.349500 -2.202900
H 2.968800 3.543400 -2.717600
C 1.980500 3.467200 2.726800
H 0.900200 3.503300 2.575400
H 2.231600 2.604900 3.352600
H 3.454200 1.856500 -2.323400
H 2.297000 4.387200 3.227300
C -4.206600 3.303900 0.123800
C -5.475500 3.416100 0.683500
C -6.518300 3.724700 -0.183900
H -5.644500 3.268500 1.745000
C -5.029800 3.810700 -2.113500
C -6.299900 3.915500 -1.556600
H -7.524700 3.823600 0.211100
H -4.865500 3.961200 -3.175600
H -7.141700 4.154600 -2.199100
C 4.206600 3.304300 -0.124300
C 5.475400 3.416800 -0.684100
C 6.518100 3.726100 0.183100
H 5.644400 3.268800 -1.745600
C 5.029800 3.812400 2.112700
C 6.299800 3.917400 1.555700
H 7.524500 3.825400 -0.212100
H 4.865600 3.963400 3.174800
H 7.141600 4.157100 2.198000
O 4.787900 0.000500 -1.775900
H 4.402900 0.000300 -0.876900
C 6.200800 0.000700 -1.670300
H 6.547700 -0.883700 -1.115800
H 6.547500 0.885300 -1.115900
C 6.784900 0.000600 -3.066200
H 7.879300 0.000800 -3.029500
H 6.458600 -0.884500 -3.623200
H 6.458300 0.885500 -3.623400
O -4.786600 0.000400 1.777700
H -4.402200 0.000200 0.878500
C -6.199600 0.000800 1.673000
H -6.546500 0.885500 1.118800
H -6.547000 -0.883600 1.118700
C -6.782900 0.000900 3.069300
H -7.877300 0.001200 3.033200
H -6.456300 -0.884300 3.626200
H -6.455800 0.885800 3.626300

II-3S2

118
Molecule Name
Au -0.175900 -2.852600 -0.131300
N 2.733900 -3.108700 -1.004400
N 2.561100 -3.152400 1.163300
C 1.845100 -3.033300 0.017400
C 3.903500 -3.315700 0.878200
C 2.428300 -2.928400 -2.407200
H 1.360400 -2.725300 -2.504400
H 2.994000 -2.072400 -2.792600
C 2.035900 -3.034800 2.506900
H 0.948500 -2.962200 2.449600

H 2.301200 -3.921700 3.090900
H 2.676000 -3.834100 -2.970100
H 2.440600 -2.132400 2.979200
N -3.088400 -3.055500 0.753600
N -2.897300 -3.350800 -1.391400
C -2.192600 -3.083700 -0.261900
C -4.240900 -3.499900 -1.100000
C -2.798500 -2.721400 2.131700
H -1.746800 -2.439800 2.205900
H -3.423500 -1.872800 2.431200
C -2.348200 -3.431200 -2.724100
H -1.259300 -3.412900 -2.656300
H -2.657500 -4.366200 -3.201000
H -2.988300 -3.583200 2.780000
H -2.687500 -2.582600 -3.326600
C 4.014500 -3.289900 -0.517000
C 5.240100 -3.457900 -1.154600
C 5.013800 -3.507700 1.695400
C 6.351500 -3.646000 -0.338900
H 5.326800 -3.447500 -2.236300
C 6.240600 -3.670200 1.059500
H 4.928700 -3.534100 2.777000
H 7.326700 -3.784700 -0.795600
H 7.131900 -3.826300 1.659300
C -4.361900 -3.315800 0.282000
C -5.588400 -3.433300 0.928500
C -5.342400 -3.794600 -1.897300
C -6.690500 -3.730100 0.133300
H -5.682400 -3.298600 2.001100
C -6.570200 -3.904900 -1.253500
H -5.253300 -3.932800 -2.970100
H -7.666700 -3.832500 0.597400
H -7.455800 -4.135400 -1.837600
N -3.345100 -0.000100 -0.499800
C -2.177700 -0.000000 -0.433700
N 2.944900 -0.000000 0.092400
C 1.779600 -0.000000 -0.023900
Au -0.198200 -0.000000 -0.233000
Au -0.175900 2.852600 -0.131400
N 2.733900 3.108700 -1.004300
N 2.561000 3.152400 1.163400
C 1.845100 3.033400 0.017400
C 3.903500 3.315800 0.878200
C 2.428300 2.928300 -2.407100
H 1.360400 2.725300 -2.504300
H 2.676100 3.834000 -2.970100
C 2.035800 3.034800 2.506900
H 0.948500 2.962200 2.449600
H 2.440600 2.132400 2.979300
H 2.994000 2.072400 -2.792500
H 2.301000 3.921700 3.090900
N -3.088400 3.055500 0.753500
N -2.897300 3.350700 -1.391500
C -2.192600 3.083700 -0.262000
C -4.240900 3.499900 -1.100100
C -2.798600 2.721600 2.131700
H -1.746800 2.440000 2.205900
H -2.988400 3.583300 2.779800
C -2.348200 3.431000 -2.724200
H -1.259300 3.412700 -2.656400
H -2.687500 2.582400 -3.326600
H -3.423600 1.872900 2.431200
H -2.657500 4.366000 -3.201200
C 4.014400 3.289900 -0.516900
C 5.240100 3.458000 -1.154500
C 6.351500 3.646100 -0.338800
H 5.326800 3.447600 -2.236200
C 5.013700 3.507800 1.695500
C 6.240500 3.670300 1.059600
H 7.326700 3.784800 -0.795400
H 4.928600 3.534200 2.777100
H 7.131800 3.826500 1.659400
C -4.361900 3.315800 0.281900
C -5.588500 3.433300 0.928400
C -6.690500 3.730100 0.133200
H -5.682500 3.298600 2.001000
C -5.342400 3.794500 -1.897500
C -6.570200 3.904800 -1.253700
H -7.666700 3.832500 0.597200
H -5.253300 3.932600 -2.970200
H -7.455800 4.135300 -1.837800
O -4.776800 0.000000 2.001400
H -4.476200 -0.000000 1.071100
C -6.193600 0.000000 2.022600
H -6.588300 -0.884500 1.501100
H -6.588300 0.884600 1.501000
C -6.651200 0.000100 3.465000
H -6.276400 0.885100 3.990700
H -7.744400 0.000100 3.525900
H -6.276400 -0.884900 3.990800
O 4.193500 -0.000000 -2.548200
H 3.950900 -0.000000 -1.603200
C 5.607200 -0.000000 -2.651100
H 6.031500 0.884800 -2.152800
H 6.031600 -0.884700 -2.152700
C 5.981000 -0.000100 -4.117200
H 5.576800 0.885200 -4.619700
H 7.069000 -0.000100 -4.239900
H 5.576900 -0.885500 -4.619600
H 3.525500 0.000000 1.975800
O 3.571400 0.000000 2.950200
C 4.935600 -0.000000 3.334800
H 5.451700 -0.884700 2.931500
H 5.451700 0.884700 2.931500
C 5.007600 -0.000000 4.846000
H 6.048800 -0.000000 5.184800
H 4.510800 0.885400 5.256800
H 4.510700 -0.885400 5.256800

II-4S2

127
Molecule Name
Au 0.000000 0.000000 -2.848500
N -1.087500 2.830000 -3.133900
N 1.087500 2.830000 -3.133900
C 0.000000 2.025300 -3.038100
C 0.699900 4.146100 -3.301200
C -2.465900 2.412800 -2.991600
H -2.489400 1.328400 -2.872600
H -2.900000 2.886800 -2.103900
C 2.465900 2.412800 -2.991600
H 2.489400 1.328400 -2.872600
H 3.035500 2.685400 -3.885700
H -3.035500 2.685400 -3.885700
H 2.900000 2.886800 -2.103900
N 1.087500 -2.830000 -3.133900
N -1.087500 -2.830000 -3.133900
C 0.000000 -2.025300 -3.038100
C -0.699900 -4.146100 -3.301200

C	2.465900	-2.412800	-2.991600
H	2.489400	-1.328400	-2.872600
H	2.900000	-2.886800	-2.103900
C	-2.465900	-2.412800	-2.991600
H	-2.489400	-1.328400	-2.872600
H	-3.035500	-2.685400	-3.885700
H	3.035500	-2.685400	-3.885700
H	-2.900000	-2.886800	-2.103900
C	-0.699900	4.146100	-3.301200
C	-1.429900	5.318300	-3.474800
C	1.429900	5.318300	-3.474800
C	-0.701500	6.491600	-3.643700
H	-2.515100	5.319100	-3.482100
C	0.701500	6.491600	-3.643700
H	2.515100	5.319100	-3.482100
H	-1.231300	7.428500	-3.785900
H	1.231300	7.428500	-3.785900
C	0.699900	-4.146100	-3.301200
C	1.429900	-5.318300	-3.474800
C	-1.429900	-5.318300	-3.474800
C	0.701500	-6.491600	-3.643700
H	2.515100	-5.319100	-3.482100
C	-0.701500	-6.491600	-3.643700
H	-2.515100	-5.319100	-3.482100
H	1.231300	-7.428500	-3.785900
H	-1.231300	-7.428500	-3.785900
N	0.000000	-3.158400	-0.000000
C	0.000000	-1.987500	-0.000100
N	0.000000	3.158400	-0.000000
C	0.000000	1.987500	-0.000100
Au	0.000000	0.000000	-0.000100
Au	0.000000	0.000000	2.848400
N	-1.087500	2.830000	3.133900
N	1.087500	2.830000	3.133900
C	0.000000	2.025300	3.038100
C	0.699900	4.146100	3.301300
C	-2.465900	2.412800	2.991500
H	-2.489400	1.328400	2.872500
H	-3.035500	2.685400	3.885600
C	2.465900	2.412800	2.991500
H	2.489400	1.328400	2.872500
H	2.900000	2.886800	2.103800
H	-2.900000	2.886800	2.103800
H	3.035500	2.685400	3.885600
N	1.087500	-2.830000	3.133900
N	-1.087500	-2.830000	3.133900
C	0.000000	-2.025300	3.038100
C	-0.699900	-4.146100	3.301300
C	2.465900	-2.412800	2.991500
H	2.489400	-1.328400	2.872500
H	3.035500	-2.685400	3.885600
C	-2.465900	-2.412800	2.991500
H	-2.489400	-1.328400	2.872500
H	-2.900000	-2.886800	2.103800
H	2.900000	-2.886800	2.103800
H	-3.035500	-2.685400	3.885600
C	-0.699900	4.146100	3.301300
C	-1.429900	5.318300	3.475100
C	-0.701500	6.491500	3.644000
H	-2.515100	5.319000	3.482400
C	1.429900	5.318300	3.475100
C	0.701500	6.491500	3.644000
H	-1.231300	7.428400	3.786300
H	2.515100	5.319000	3.482400
H	1.231300	7.428400	3.786300

C	0.699900	-4.146100	3.301300
C	1.429900	-5.318300	3.475100
C	0.701500	-6.491500	3.644000
H	2.515100	-5.319000	3.482400
C	-1.429900	-5.318300	3.475100
C	-0.701500	-6.491500	3.644000
H	1.231300	-7.428400	3.786300
H	-2.515100	-5.319000	3.482400
H	-1.231300	-7.428400	3.786300
O	2.789100	-4.061200	-0.000000
H	1.821700	-3.939200	-0.000100
C	3.068200	-5.450900	-0.000100
H	2.626500	-5.934500	-0.884700
H	2.626500	-5.934600	0.884600
C	4.569400	-5.637600	-0.000100
H	5.016800	-5.173300	0.885400
H	4.828300	-6.701400	-0.000100
H	5.016800	-5.173300	-0.885500
O	-2.789100	4.061200	-0.000000
H	-1.821700	3.939200	-0.000100
C	-3.068200	5.450900	-0.000100
H	-2.626500	5.934600	0.884600
H	-2.626500	5.934500	-0.884700
C	-4.569400	5.637600	-0.000100
H	-5.016800	5.173300	0.885400
H	-4.828300	6.701400	-0.000100
H	-5.016800	5.173300	-0.885500
H	1.821700	3.939200	-0.000100
O	2.789100	4.061200	-0.000000
C	3.068200	5.450900	-0.000100
H	2.626500	5.934500	-0.884700
H	2.626500	5.934600	0.884600
C	4.569400	5.637600	-0.000100
H	4.828300	6.701400	-0.000100
H	5.016800	5.173300	0.885400
H	5.016800	5.173300	-0.885500
H	-1.821700	-3.939200	-0.000100
O	-2.789100	-4.061200	-0.000000
C	-3.068200	-5.450900	-0.000100
H	-2.626500	-5.934600	0.884600
H	-2.626500	-5.934500	-0.884700
C	-4.569400	-5.637600	-0.000100
H	-4.828300	-6.701400	-0.000100
H	-5.016800	-5.173300	0.885400
H	-5.016800	-5.173300	-0.885500

21. The T_1 .xyz files of III and its complexes

III

```
67
Molecule Name
Au     2.755900    0.000100    0.000100
N      2.824700    2.899200   -0.948800
N      2.891900    2.797200    1.202900
C      2.833700    2.023300    0.088800
C      2.937700    4.130400    0.865000
H      2.995600    4.911000    1.607900
C      2.896800    4.194900   -0.490000
H      2.915100    5.042300   -1.158000
C      2.813200    2.510300   -2.346700
H      2.023800    1.775800   -2.524200
H      2.620600    3.394800   -2.956200
C      2.945700    2.276100    2.556700
H      3.863400    1.696800    2.696800
H      2.944100    3.114600    3.255000
N      0.000300    0.000200   -3.192000
C      0.000200    0.000100   -2.021400
H      3.781100    2.084400   -2.629100
H      2.077800    1.641600    2.753400
N      2.824600   -2.899100    0.948900
N      2.892100   -2.797100   -1.202700
C      2.833800   -2.023100   -0.088700
C      2.937900   -4.130300   -0.864800
H      2.995800   -4.910900   -1.607700
C      2.896800   -4.194800    0.490100
H      2.914900   -5.042100    1.158100
C      2.812900   -2.510100    2.346800
H      2.023200   -1.776000    2.524300
H      2.620800   -3.394700    2.956300
C      2.946200   -2.276000   -2.556500
H      3.863900   -1.696700   -2.696400
H      2.944600   -3.114500   -3.254800
N     -0.000300   -0.000100    3.191700
C     -0.000200   -0.000100    2.021100
H      3.780600   -2.083800    2.629200
H      2.078300   -1.641500   -2.753300
Au    -2.755900   -0.000100    0.000000
N     -2.892200    2.797100   -1.202700
N     -2.824800    2.899000    0.948900
C     -2.833900    2.023100   -0.088800
C     -2.897100    4.194800    0.490100
H     -2.915300    5.042100    1.158200
C     -2.938000    4.130300   -0.864800
H     -2.995900    4.910900   -1.607700
C     -2.946000    2.276100   -2.556600
H     -2.078100    1.641600   -2.753300
H     -3.863700    1.696800   -2.696700
C     -2.813300    2.510000    2.346800
H     -3.781200    2.084100    2.629100
H     -2.023900    1.775600    2.524300
H     -2.944400    3.114600   -3.254900
H     -2.620800    3.394600    2.956300
N     -2.891800   -2.797400    1.202700
N     -2.824500   -2.899100   -0.949000
C     -2.833600   -2.023300    0.088800
C     -2.896600   -4.194900   -0.490400
H     -2.914700   -5.042100   -1.158500
C     -2.937500   -4.130600    0.864600
H     -2.995400   -4.911300    1.607400
C     -2.945900   -2.276500    2.556600
H     -2.078000   -1.642000    2.753500
H     -3.863500   -1.697200    2.696500
C     -2.812900   -2.509900   -2.346800
H     -3.780800   -2.084000   -2.629200
H     -2.023500   -1.775500   -2.524200
H     -2.944400   -3.115100    3.254800
H     -2.620400   -3.394400   -2.956500
Ag     0.000000    0.000000   -0.000200
```

III-S1

```
73
Molecule Name
Au     2.755900   -0.157300   -0.162700
N      2.943800   -2.655100    1.580400
N      2.971700   -3.178600   -0.509900
C      2.906100   -2.116600    0.333900
C      3.068400   -4.355200    0.197400
H      3.139700   -5.315600   -0.289400
C      3.051900   -4.026100    1.513700
H      3.107900   -4.643300    2.397200
C      2.940400   -1.878300    2.804900
H      2.123200   -1.152900    2.784100
H      2.796100   -2.553200    3.650300
C      2.992600   -3.069800   -1.956900
H      3.911700   -2.572200   -2.281600
H      2.960000   -4.073200   -2.384700
N      0.000500    0.762600    2.937400
C      0.000200    0.359600    1.838600
H      3.893500   -1.353500    2.924200
H      2.125500   -2.503000   -2.304900
N      2.740500    2.323800   -1.927800
N      2.760900    2.887100    0.151800
C      2.765300    1.809000   -0.671300
C      2.746800    4.053800   -0.577000
H      2.743600    5.024700   -0.105900
C      2.737500    3.699500   -1.886900
H      2.732000    4.302000   -2.782300
C      2.783000    1.526400   -3.139800
H      3.764000    1.053200   -3.246300
H      2.005800    0.758800   -3.113000
C      2.807400    2.836900    1.602400
H      3.745900    3.274700    1.956100
H      1.952200    3.368400    2.032400
N     -0.002800   -1.090500   -3.171000
C     -0.002000   -0.772600   -2.044200
H      2.608000    2.180200   -3.995900
H      2.758900    1.792500    1.912100
Au    -2.757300   -0.149100   -0.161800
N     -2.953400   -2.645200    1.582700
N     -2.982800   -3.170000   -0.507200
C     -2.914000   -2.107700    0.335800
C     -3.083100   -4.345900    0.201000
H     -3.157400   -5.306400   -0.285200
C     -3.065600   -4.015800    1.517000
H     -3.123500   -4.632200    2.400900
C     -2.947300   -1.867400    2.806700
H     -2.126500   -1.146000    2.786200
H     -3.897900   -1.337900    2.924700
C     -3.003600   -3.062400   -1.954300
```

H -3.921500 -2.563000 -2.279400
H -2.135200 -2.497700 -2.302700
H -2.807200 -2.542500 3.652700
H -2.973100 -4.066100 -2.381300
N -2.733400 2.330400 -1.928600
N -2.748700 2.895700 0.150600
C -2.759100 1.816900 -0.671500
C -2.729900 4.061600 -0.579200
H -2.721800 5.032900 -0.109000
C -2.723800 3.706100 -1.888800
H -2.716100 4.307800 -2.784800
C -2.780200 1.532100 -3.139800
H -3.762400 1.060800 -3.243500
H -2.606200 2.184900 -3.996900
C -2.794800 2.846800 1.601200
H -3.733300 3.284500 1.955000
H -2.745600 1.802800 1.912000
H -2.004500 0.763000 -3.114400
H -1.939700 3.378900 2.030500
Ag -0.000700 -0.218700 -0.101700
O 0.007600 3.644700 3.185000
H 0.004800 2.671400 3.270400
C 0.013700 4.202100 4.482400
H 0.906500 3.913900 5.055200
H 0.015700 5.289800 4.371700
H -0.875900 3.917800 5.062000

III-2S1

79
Molecule Name
Au -2.935300 -0.000100 -0.000000
N -2.977900 2.986700 0.819100
N -3.427700 2.737200 -1.280200
C -3.121900 2.038700 -0.149400
C -3.475900 4.094100 -1.018300
H -3.699600 4.819700 -1.784500
C -3.197200 4.250600 0.301900
H -3.137900 5.136900 0.912700
C -2.599500 2.740100 2.205700
H -2.311400 1.692500 2.305600
H -1.747200 3.379100 2.450300
C -3.578500 2.145200 -2.603400
H -4.152200 1.218400 -2.523600
H -4.119500 2.843700 -3.245000
H -3.439300 2.949700 2.875800
H -2.595900 1.938200 -3.037200
N -2.977800 -2.986800 -0.819100
N -3.427500 -2.737300 1.280200
C -3.121800 -2.038800 0.149400
C -3.475600 -4.094200 1.018300
H -3.699300 -4.819800 1.784600
C -3.197000 -4.250700 -0.301800
H -3.137700 -5.137100 -0.912600
C -2.599400 -2.740300 -2.205700
H -2.311300 -1.692700 -2.305600
H -1.747100 -3.379300 -2.450300
C -3.578400 -2.145300 2.603400
H -4.152200 -1.218500 2.523500
H -4.119300 -2.843700 3.245100
H -3.439300 -2.949800 -2.875800
H -2.595800 -1.938100 3.037200
N 0.000000 -2.911300 1.350200
C 0.000000 -1.818800 0.924300
N -0.000200 2.911300 -1.350200
C -0.000000 1.818800 -0.924100
Ag 0.000100 -0.000000 0.000100
Au 2.935500 0.000100 0.000000
N 2.977900 2.986900 0.818600
N 3.427700 2.737100 -1.280500
C 3.121900 2.038800 -0.149700
C 3.475800 4.094100 -1.018900
H 3.699900 4.819500 -1.785200
C 3.197100 4.250800 0.301200
H 3.137600 5.137200 0.912000
C 2.598900 2.740700 2.205200
H 2.310500 1.693200 2.305200
H 3.438500 2.950300 2.875600
C 3.578500 2.144900 -2.603700
H 4.152800 1.218500 -2.523900
H 2.595800 1.937200 -3.037100
H 1.746500 3.379900 2.449100
H 4.118700 2.843600 -3.245700
N 2.977900 -2.986700 -0.818800
N 3.427900 -2.737000 1.280400
C 3.121900 -2.038700 0.149600
C 3.476000 -4.094000 1.018600
H 3.700100 -4.819500 1.784900
C 3.197100 -4.250600 -0.301400
H 3.137600 -5.137000 -0.912200
C 2.598700 -2.740500 -2.205300
H 2.310200 -1.693000 -2.305200
H 3.438300 -2.949800 -2.875800
C 3.578600 -2.145000 2.603500
H 4.152900 -1.218500 2.523900
H 2.595900 -1.937200 3.036900
H 1.746500 -3.379800 -2.449200
H 4.118800 -2.843700 3.245600
O -0.000500 -4.631300 -1.130700
H -0.000600 -4.137800 -0.285800
C 0.000100 -6.020100 -0.831400
H -0.889400 -6.323000 -0.260400
H -0.000200 -6.559700 -1.783000
H 0.890200 -6.322500 -0.261100
O -0.000700 4.631200 1.130700
H -0.000600 4.137800 0.285900
C -0.000300 6.020000 0.831600
H -0.001000 6.559600 1.783200
H 0.890000 6.322600 0.261700
H -0.889500 6.322900 0.260300

III-3S1

85
Molecule Name
Au 0.072000 2.856400 -0.104700
N -2.844900 2.937000 -1.057000
N -2.714200 3.169400 1.077300
C -1.957000 2.999900 -0.034800
C -4.050200 3.210500 0.753500
H -4.819200 3.340700 1.499300
C -4.132600 3.067500 -0.592400
H -4.984600 3.054300 -1.254000
C -2.512500 2.712800 -2.449200
H -1.449900 2.471100 -2.516200
H -3.103300 1.869600 -2.818400
C -2.201200 3.187600 2.433500
H -1.180900 3.578100 2.417800

H -2.820000 3.851500 3.042000
H -2.715500 3.610200 -3.041500
H -2.209300 2.178500 2.860500
N 2.971000 2.905800 0.888000
N 2.855700 3.367500 -1.209000
C 2.093000 3.050500 -0.133800
C 4.186000 3.421100 -0.863200
H 4.958700 3.651300 -1.580300
C 4.258000 3.135300 0.460000
H 5.102400 3.072700 1.127600
C 2.628500 2.536500 2.245700
H 1.605300 2.155000 2.255200
H 3.312800 1.749100 2.571700
C 2.343500 3.509500 -2.554500
H 1.425100 4.102300 -2.534800
H 3.084900 4.026100 -3.166800
H 2.696600 3.402900 2.911000
H 2.139500 2.524000 -2.984500
N 3.246300 0.038800 -1.120100
C 2.101300 0.027000 -0.874800
N -2.909900 -0.055900 0.554000
C -1.774900 -0.032300 0.260400
Ag 0.164300 0.002100 -0.298600
Au 0.175700 -2.848400 -0.123200
N -2.695200 -2.947700 -1.206600
N -2.643500 -3.323500 0.910900
C -1.848000 -3.056900 -0.154100
C -3.963500 -3.380400 0.528400
H -4.757800 -3.582200 1.230400
C -3.995800 -3.147500 -0.807200
H -4.820200 -3.111000 -1.501800
C -2.310400 -2.615900 -2.562700
H -1.282800 -2.245600 -2.547600
H -2.368400 -3.497200 -3.208900
C -2.183800 -3.419800 2.283200
H -1.159300 -3.799800 2.284600
H -2.219600 -2.438600 2.770000
H -2.973700 -1.828000 -2.929500
H -2.819200 -4.124900 2.824600
N 3.034800 -2.768400 0.989200
N 3.016300 -3.306500 -1.092400
C 2.202400 -2.977500 -0.059200
C 4.333000 -3.304000 -0.694900
H 5.139900 -3.534900 -1.373100
C 4.344100 -2.969900 0.618900
H 5.159400 -2.856300 1.315300
C 2.630700 -2.351800 2.315900
H 1.583600 -2.044500 2.280200
H 2.740500 -3.174600 3.029100
C 2.564500 -3.511100 -2.451700
H 1.637000 -4.089600 -2.442700
H 2.395300 -2.546700 -2.940200
H 3.251600 -1.503500 2.615600
H 3.324500 -4.069500 -3.001500
O 4.749600 0.110200 1.415300
H 4.319600 0.092600 0.538000
C 6.144400 0.114600 1.213900
H 6.484100 0.997800 0.653200
H 6.492400 -0.778900 0.675500
H 6.621100 0.129500 2.198200
O -4.428600 -0.029300 -1.962000
H -4.021200 -0.041300 -1.075400
C -5.828400 -0.056000 -1.803100
H -6.175100 -0.963000 -1.286100
H -6.204700 0.813800 -1.244700
H -6.274700 -0.040200 -2.801200
C -4.027100 -0.158300 4.140300
O -2.800000 -0.154400 3.446600
H -4.644400 -1.036400 3.898300
H -4.624200 0.744800 3.943400
H -2.987900 -0.127500 2.490300
H -3.800200 -0.187800 5.209100

III-4S1

91
Molecule Name
Au -0.000000 -2.867300 -0.001000
N -2.852700 -3.115000 1.073200
N -2.851900 -3.112500 -1.077900
C -2.029900 -3.039800 -0.001900
C -4.162800 -3.235300 -0.680800
H -4.972600 -3.314300 -1.389800
C -4.163300 -3.236900 0.674900
H -4.973700 -3.317400 1.383100
C -2.442100 -2.996300 2.458600
H -1.354900 -2.910000 2.489500
H -2.893400 -2.099400 2.895900
C -2.440300 -2.991600 -2.462700
H -1.353200 -2.902400 -2.492600
H -2.739800 -3.882000 -3.023100
H -2.744400 -3.886500 3.017800
H -2.893500 -2.095500 -2.899500
N 2.853000 -3.112800 -1.075000
N 2.851800 -3.113100 1.076100
C 2.029900 -3.039300 0.000100
C 4.162800 -3.235000 0.679100
H 4.972500 -3.314600 1.388100
C 4.163500 -3.234800 -0.676600
H 4.974000 -3.314100 -1.384800
C 2.442500 -2.993100 -2.460300
H 1.355500 -2.904300 -2.491200
H 2.895800 -2.097200 -2.897600
C 2.439900 -2.993600 2.461000
H 1.352700 -2.905800 2.490800
H 2.740400 -3.884100 3.020800
H 2.742800 -3.883900 -3.019700
H 2.891900 -2.097300 2.898500
N 3.185800 0.000000 0.000100
C 2.013700 0.000100 -0.000200
N -3.186700 -0.000100 0.003800
C -2.014500 0.000000 0.002200
Ag -0.000400 -0.000200 0.000400
Au -0.000100 2.867500 -0.000900
N -2.852900 3.114900 1.073200
N -2.852000 3.112300 -1.077900
C -2.030000 3.039700 -0.002000
C -4.162900 3.235100 -0.680900
H -4.972700 3.313900 -1.389900
C -4.163500 3.236700 0.674800
H -4.973900 3.317100 1.383000
C -2.442300 2.996300 2.458600
H -1.355100 2.910100 2.489500
H -2.744700 3.886500 3.017800
C -2.440300 2.991300 -2.462800
H -1.353300 2.902200 -2.492500
H -2.893500 2.095100 -2.899500
H -2.893500 2.099400 2.895900

H	-2.739900	3.881600	-3.023300
N	2.852700	3.112700	-1.075300
N	2.851800	3.113300	1.075900
C	2.029800	3.039300	-0.000000
C	4.162700	3.235100	0.678700
H	4.972600	3.314700	1.387600
C	4.163300	3.234800	-0.677000
H	4.973800	3.314000	-1.385300
C	2.442200	2.992900	-2.460500
H	1.355100	2.904200	-2.491300
H	2.742400	3.883700	-3.020000
C	2.440000	2.993800	2.460800
H	1.352900	2.906200	2.490700
H	2.892000	2.097400	2.898300
H	2.895400	2.097000	-2.897700
H	2.740700	3.884300	3.020500
O	4.083500	0.000000	-2.751000
H	3.899400	0.000000	-1.792500
C	5.480800	0.000100	-2.931400
H	5.961000	-0.889700	-2.497200
H	5.961000	0.889900	-2.497200
H	5.674700	0.000100	-4.007300
O	-4.076200	-0.000000	2.758300
H	-3.895000	-0.000000	1.799200
C	-5.472900	-0.000100	2.943100
H	-5.954500	0.889700	2.510400
H	-5.954400	-0.890000	2.510400
H	-5.663300	-0.000100	4.019600
C	-5.480200	-0.000100	-2.929400
O	-4.083000	-0.000200	-2.748700
H	-5.960500	0.889700	-2.495300
H	-5.960600	-0.889900	-2.495200
H	-3.899100	-0.000200	-1.790100
H	-5.673900	-0.000100	-4.005300
C	5.475300	0.000100	2.936400
O	4.078400	0.000100	2.753700
H	5.956300	0.889900	2.503000
H	5.956300	-0.889800	2.503000
H	3.895800	0.000000	1.794900
H	5.667400	0.000100	4.012600

III-S2

76

Molecule Name

Au	2.754900	-0.283700	-0.206200
N	2.658700	1.873200	-2.356000
N	2.706000	2.769400	-0.396900
C	2.715000	1.571100	-1.033000
C	2.657800	3.800700	-1.306000
H	2.646800	4.835500	-0.999900
C	2.631600	3.236900	-2.540000
H	2.600300	3.684500	-3.521500
C	2.699700	0.889800	-3.422800
H	3.682600	0.409800	-3.455100
H	1.926800	0.133100	-3.268700
C	2.783200	2.959300	1.040800
H	3.701500	3.499800	1.290100
H	1.908100	3.506800	1.406200
N	0.000100	-1.715500	-2.990600
C	0.000100	-1.222800	-1.928500
H	2.519400	1.396000	-4.372700
H	2.806600	1.978400	1.516700
N	3.040900	-2.438600	1.931600
N	3.018800	-3.319700	-0.034700
C	2.957400	-2.127600	0.612100
C	3.157600	-4.352800	0.864100
H	3.233400	-5.382600	0.550300
C	3.171800	-3.798300	2.102500
H	3.263000	-4.250300	3.078100
C	3.050200	-1.457800	2.999800
H	2.198500	-0.781100	2.893100
H	2.975400	-1.977600	3.956500
C	3.000700	-3.466600	-1.478400
H	3.920100	-3.058500	-1.909600
H	2.932500	-4.528100	-1.722400
N	-0.003300	1.055200	2.759200
C	-0.001700	0.494100	1.732200
H	3.980800	-0.882100	2.976300
H	2.136000	-2.946700	-1.898000
Au	-2.755200	-0.282200	-0.206500
N	-2.655400	1.877200	-2.353500
N	-2.707100	2.771200	-0.393600
C	-2.714300	1.573700	-1.031100
C	-2.657600	3.803600	-1.301500
H	-2.647400	4.838000	-0.994100
C	-2.628700	3.241200	-2.536000
H	-2.595700	3.689900	-3.517000
C	-2.694400	0.895000	-3.421400
H	-3.679000	0.418900	-3.459900
H	-2.506500	1.401200	-4.369900
C	-2.787100	2.959700	1.044100
H	-3.706700	3.498600	1.292300
H	-2.809800	1.978400	1.519100
H	-1.925400	0.135100	-3.263600
H	-1.913400	3.508200	1.411400
N	-3.042400	-2.439300	1.928900
N	-3.020200	-3.318300	-0.038300
C	-2.958500	-2.126900	0.609800
C	-3.159500	-4.352300	0.859400
H	-3.235500	-5.381700	0.544600
C	-3.173700	-3.799100	2.098400
H	-3.265200	-4.252100	3.073600
C	-3.051900	-1.459600	2.998200
H	-2.200800	-0.782100	2.891700
H	-3.983000	-0.884600	2.975900
C	-3.001900	-3.463800	-1.482100
H	-3.920700	-3.053900	-1.913100
H	-2.136400	-2.944600	-1.900900
H	-2.976100	-1.980300	3.954200
H	-2.935200	-4.525100	-1.727100
Ag	-0.000100	-0.370900	-0.097600
C	0.004400	4.697700	3.758100
C	0.007100	6.167400	3.400600
H	0.891200	4.456200	4.363800
H	-0.880000	4.460700	4.369000
H	-0.881000	6.422700	2.812800
H	0.011100	6.785300	4.304200
H	0.893100	6.418400	2.807800
O	-0.001100	3.941000	2.558800
H	-0.001300	2.992000	2.792600

III-2S2

85

Molecule Name

Au	0.000700	-2.938600	0.000100
N	3.045200	-2.971300	-0.568300

N	2.623200	-3.432300	1.500600
C	2.020400	-3.123300	0.316800
C	3.997500	-3.473900	1.352800
H	4.657600	-3.698000	2.176000
C	4.262500	-3.188300	0.051700
H	5.196400	-3.121500	-0.482500
C	2.913200	-2.589400	-1.969600
H	1.881100	-2.284400	-2.150000
H	3.582900	-1.747500	-2.162400
C	1.922900	-3.594700	2.768200
H	1.013000	-4.178900	2.608900
H	2.570800	-4.129400	3.465600
H	3.160500	-3.432500	-2.622600
H	1.667400	-2.616500	3.186200
N	-3.043900	-2.972700	0.568300
N	-2.621500	-3.433400	-1.500600
C	-2.018900	-3.124200	-0.316800
C	-3.995800	-3.475600	-1.353000
H	-4.655700	-3.700000	-2.176200
C	-4.261000	-3.190200	-0.051800
H	-5.195000	-3.123700	0.482200
C	-2.912100	-2.590700	1.969500
H	-1.880200	-2.285400	2.150100
H	-3.582200	-1.749000	2.162300
C	-1.920900	-3.595600	-2.768200
H	-1.010900	-4.179400	-2.608800
H	-2.568600	-4.130500	-3.465700
H	-3.159300	-3.433900	2.622500
H	-1.665800	-2.617300	-3.186100
N	-2.828400	-0.000500	-1.515900
C	-1.762600	-0.000300	-1.027400
N	2.828400	0.000800	1.515900
C	1.762600	0.000500	1.027500
Ag	0.000000	-0.000000	0.000100
Au	-0.000700	2.938600	-0.000100
N	3.043500	2.972200	-0.570400
N	2.622500	3.433700	1.498600
C	2.019100	3.124200	0.315300
C	3.996700	3.475700	1.350100
H	4.657200	3.700200	2.172800
C	4.261000	3.189800	0.048900
H	5.194700	3.122900	-0.485800
C	2.910900	2.589700	-1.971400
H	1.878900	2.284100	-2.151200
H	3.157300	3.432800	-2.624900
C	1.922900	3.596300	2.766600
H	1.012500	4.179800	2.607500
H	1.668300	2.618100	3.185200
H	3.581000	1.748300	-2.164400
H	2.570900	4.131800	3.463400
N	-3.044900	2.970900	0.570500
N	-2.624200	3.432600	-1.498600
C	-2.020600	3.123300	-0.315300
C	-3.998400	3.474000	-1.349900
H	-4.659100	3.698300	-2.172600
C	-4.262500	3.188000	-0.048700
H	-5.196200	3.120700	0.486000
C	-2.911900	2.588400	1.971400
H	-1.879800	2.283100	2.151100
H	-3.158500	3.431400	2.625000
C	-1.924800	3.595500	-2.766600
H	-1.014600	4.179400	-2.607600
H	-1.669800	2.617500	-3.185300
H	-3.581700	1.746700	2.164500
H	-2.573100	4.130800	-3.463300
O	-4.764000	-0.001000	0.789300
H	-4.202800	-0.001000	-0.012800
C	-6.130900	-0.001600	0.380800
H	-6.348700	-0.886500	-0.236900
H	-6.348900	0.882000	-0.238800
C	-7.011400	-0.000500	1.620400
H	-8.070500	-0.001000	1.341400
H	-6.817200	-0.885500	2.236600
H	-6.817600	0.886000	2.234800
O	4.764000	0.000800	-0.789500
H	4.202800	0.000500	0.012700
C	6.130900	0.001000	-0.381100
H	6.348600	0.884800	0.238300
H	6.349200	-0.883700	0.236900
C	7.011300	0.002200	-1.620700
H	6.817100	0.888300	-2.235300
H	8.070500	0.002300	-1.341800
H	6.817600	-0.883100	-2.236600

III-3S2

94

Molecule Name

Au	0.176100	2.853300	-0.184600
N	-2.766000	2.966100	-1.050900
N	-2.569700	3.231000	1.074600
C	-1.848000	3.032000	-0.055800
C	-3.913900	3.287100	0.789000
H	-4.658800	3.440200	1.554700
C	-4.037500	3.123500	-0.551400
H	-4.908800	3.110900	-1.187400
C	-2.476700	2.709700	-2.447100
H	-1.424200	2.432900	-2.535800
H	-3.101800	1.879100	-2.787000
C	-2.019500	3.268600	2.415900
H	-0.975800	3.585700	2.356400
H	-2.573300	3.998400	3.011900
H	-2.666600	3.603300	-3.049600
H	-2.084900	2.279800	2.883700
N	3.110200	2.825300	0.708100
N	2.927200	3.348000	-1.369500
C	2.198400	3.013000	-0.276400
C	4.269800	3.370700	-1.071400
H	5.019900	3.608000	-1.809800
C	4.384700	3.046300	0.239800
H	5.251700	2.949300	0.873400
C	2.811500	2.420200	2.065700
H	1.784200	2.051200	2.100500
H	3.497200	1.615900	2.343600
C	2.368800	3.539000	-2.690500
H	1.463900	4.148500	-2.619700
H	3.097700	4.058700	-3.315000
H	2.914500	3.265400	2.753500
H	2.129200	2.570700	-3.140400
N	3.267500	-0.000500	-1.338600
C	2.132500	-0.000300	-1.051200
N	-2.823300	0.000500	0.559100
C	-1.699700	0.000300	0.224500
Ag	0.217100	-0.000100	-0.405200
Au	0.174800	-2.853400	-0.184600
N	-2.767700	-2.965600	-1.049800
N	-2.570600	-3.229800	1.075600
C	-1.849300	-3.031400	-0.055100

C	-3.914900	-3.285700	0.790700
H	-4.659500	-3.438400	1.556700
C	-4.039000	-3.122500	-0.549800
H	-4.910600	-3.110000	-1.185400
C	-2.479000	-2.709500	-2.446200
H	-1.425800	-2.435400	-2.535800
H	-2.671600	-3.602600	-3.048700
C	-2.019900	-3.267400	2.416800
H	-0.975900	-3.583600	2.356800
H	-2.085900	-2.278700	2.884900
H	-3.102300	-1.877300	-2.785500
H	-2.572900	-3.997800	3.012700
N	3.109100	-2.826600	0.707400
N	2.925600	-3.348800	-1.370300
C	2.197100	-3.013900	-0.277000
C	4.268300	-3.371900	-1.072400
H	5.018100	-3.609300	-1.811000
C	4.383400	-3.047800	0.238900
H	5.250600	-2.951100	0.872300
C	2.810700	-2.421500	2.065100
H	1.783600	-2.052200	2.100100
H	2.913600	-3.266900	2.752700
C	2.366900	-3.539400	-2.691200
H	1.461400	-4.147800	-2.620200
H	2.128400	-2.570900	-3.141300
H	3.496800	-1.617400	2.343000
H	3.095200	-4.060000	-3.315600
O	4.913800	-0.000900	1.106700
H	4.444100	-0.000800	0.249300
C	6.304700	-0.001100	0.847100
H	6.590100	0.882000	0.253800
H	6.590000	-0.884400	0.254200
C	7.045600	-0.000800	2.166500
H	6.786400	-0.885900	2.757600
H	8.128100	-0.001000	2.003800
H	6.786700	0.884600	2.757200
O	-4.441400	0.001400	-1.895500
H	-4.015100	0.001200	-1.017400
C	-5.844500	0.000900	-1.715100
H	-6.164100	-0.882800	-1.139800
H	-6.164700	0.884300	-1.139700
C	-6.507900	0.000800	-3.074600
H	-7.598100	0.000400	-2.974700
H	-6.214300	0.886400	-3.648000
H	-6.213700	-0.884700	-3.648100
C	-4.027400	0.000500	4.107800
O	-2.775200	0.000500	3.448300
H	-4.619000	-0.885100	3.823700
H	-4.619400	0.885600	3.822900
H	-2.933300	0.000300	2.485400
C	-3.786500	0.001200	5.601100
H	-3.215900	0.887400	5.897200
H	-4.735200	0.001200	6.147300
H	-3.215400	-0.884400	5.898000

III-4S2

103
Molecule Name

Au	0.000300	2.868000	-0.001200
N	2.853300	3.111300	1.073000
N	2.852500	3.108900	-1.078100
C	2.030200	3.038500	-0.002100
C	4.163700	3.227400	-0.681000
H	4.974100	3.302200	-1.389800
C	4.164200	3.228900	0.674700
H	4.975100	3.305300	1.382700
C	2.441900	2.994600	2.458200
H	1.354700	2.907200	2.488500
H	2.893600	2.098700	2.897100
C	2.440100	2.989500	-2.462700
H	1.353100	2.899700	-2.492000
H	2.738600	3.880900	-3.022200
H	2.742900	3.886000	3.016200
H	2.893500	2.094100	-2.900700
N	-2.852900	3.109300	-1.075600
N	-2.851900	3.109800	1.075500
C	-2.029700	3.038100	-0.000400
C	-4.163200	3.227500	0.678300
H	-4.973600	3.303100	1.387100
C	-4.163800	3.227100	-0.677400
H	-4.974800	3.302300	-1.385500
C	-2.441600	2.991000	-2.460700
H	-1.354600	2.901200	-2.490900
H	-2.895300	2.096000	-2.899200
C	-2.439500	2.992100	2.460300
H	-1.352300	2.904000	2.489700
H	-2.739400	3.883500	3.019000
H	-2.740500	3.882900	-3.019200
H	-2.891500	2.096400	2.899100
N	-3.185900	-0.000000	0.000000
C	-2.013900	-0.000000	-0.000300
N	3.186900	-0.000000	0.002800
C	2.014900	-0.000000	0.001400
Ag	0.000500	-0.000000	0.000000
Au	0.000200	-2.868000	-0.001200
N	2.853200	-3.111400	1.073100
N	2.852400	-3.108900	-1.078000
C	2.030100	-3.038500	-0.002000
C	4.163700	-3.227400	-0.680900
H	4.974000	-3.302300	-1.389700
C	4.164200	-3.229000	0.674800
H	4.975000	-3.305400	1.382900
C	2.441800	-2.994600	2.458300
H	1.354600	-2.907200	2.488600
H	2.742700	-3.886100	3.016300
C	2.440200	-2.989500	-2.462600
H	1.353100	-2.899700	-2.491900
H	2.893500	-2.094100	-2.900600
H	2.893500	-2.098700	2.897200
H	2.738700	-3.880900	-3.022100
N	-2.852900	-3.109300	-1.075700
N	-2.852000	-3.109800	1.075400
C	-2.029700	-3.038100	-0.000400
C	-4.163300	-3.227400	0.678200
H	-4.973700	-3.303000	1.387000
C	-4.163900	-3.227100	-0.677500
H	-4.974800	-3.302400	-1.385600
C	-2.441600	-2.991100	-2.460700
H	-1.354600	-2.901300	-2.490900
H	-2.740500	-3.883000	-3.019200
C	-2.439600	-2.992000	2.460200
H	-1.352500	-2.904000	2.489700
H	-2.891600	-2.096200	2.898900
H	-2.895200	-2.096000	-2.899200
H	-2.739600	-3.883300	3.019000
O	-4.085600	-0.000100	-2.759000
H	-3.907300	-0.000100	-1.799100

C	-5.486700	-0.000000	-2.954800
H	-5.947100	0.884500	-2.485100
H	-5.947200	-0.884500	-2.485100
C	-5.768300	0.000000	-4.441000
H	-5.333000	-0.885800	-4.915100
H	-6.846300	0.000000	-4.631900
H	-5.333000	0.885800	-4.915100
O	4.076300	-0.000100	2.766000
H	3.901000	-0.000000	1.805600
C	5.476900	-0.000000	2.966000
H	5.938700	-0.884500	2.497600
H	5.938700	0.884500	2.497600
C	5.754000	-0.000100	4.453000
H	6.831400	-0.000000	4.647100
H	5.317300	0.885700	4.925800
H	5.317300	-0.885900	4.925700
C	5.486400	-0.000000	-2.953700
O	4.085400	0.000000	-2.757500
H	5.946900	-0.884500	-2.484100
H	5.947000	0.884400	-2.484100
H	3.907300	0.000000	-1.797600
C	5.767600	0.000000	-4.440000
H	5.332200	0.885800	-4.913900
H	6.845500	-0.000000	-4.631200
H	5.332100	-0.885800	-4.914000
C	-5.480600	0.000100	2.960400
O	-4.079800	0.000100	2.761700
H	-5.942000	-0.884400	2.491700
H	-5.941900	0.884600	2.491600
H	-3.903500	0.000100	1.801500
C	-5.759000	0.000200	4.447200
H	-6.836600	0.000200	4.640400
H	-5.322700	-0.885600	4.920400
H	-5.322700	0.886000	4.920300
